# New antiproliferative 3-substituted oxindoles inhibiting EGFR/VEGFR-2 and tubulin polymerization

**DOI:** 10.1007/s11030-023-10603-z

**Published:** 2023-02-15

**Authors:** Hend A. A. Ezelarab, Taha F. S. Ali, Samar H. Abbas, Ahmed M. Sayed, Eman A. M. Beshr, Heba A. Hassan

**Affiliations:** 1 Department of Medicinal Chemistry, Faculty of Pharmacy, Minia University, 61519-Mini, Minia, Egypt; 2 Department of Pharmacognosy, Faculty of Pharmacy, Nahda University, Beni-Suef, 62513 Egypt

**Keywords:** Antiproliferative activity, Receptor tyrosine kinases (RTKs), Tubulin polymerization inhibitors, 3-Substituted oxindole, MCF-7, Molecular-docking studies

## Abstract

New 3-substituted oxindole derivatives were designed and synthesized as antiproliferative agents. The antiproliferative activity of compounds **6a–j** was evaluated against 60 NCI cell lines. Among these tested compounds, compounds **6f** and **6g** showed remarkable antiproliferative activity, specifically against leukemia and breast cancer cell lines. Compound **6f** was the most promising antiproliferative agent against MCF-7 (human breast cancer) with an IC_50_ value of 14.77 µM compared to 5-fluorouracil **(5FU)** (IC_50_ = 2.02 µM). Notably, compound **6f** hampered receptor tyrosine EGFR fundamentally with an IC_50_ value of 1.38 µM, compared to the reference sunitinib with an IC_50_ value of 0.08 µM. Moreover, compound **6f** afforded anti-tubulin polymerization activity with an IC_50_ value of 7.99 µM as an outstanding observable activity compared with the reference combretastatin A4 with an IC_50_ value of 2.64 µM. In silico molecular-docking results of compound **6f** in the ATP-binding site of EGFR agreed with the in vitro results. Besides, the investigation of the physicochemical properties of compound **6f** via the egg-boiled method clarified good lipophilicity, GIT absorption, and blood–brain barrier penetration properties.

**Supplementary Information:**

The online version contains supplementary material available at 10.1007/s11030-023-10603-z.

## Introduction

Cancer represents the utmost challenging disease in authority for the highest mortality rate worldwide, subsequently cardiovascular diseases [1–4]. Breast cancer is a principle cause of higher cancer mortality among women globally. It was accounted that about 14% of 23% of the total breast cancer cases died [5]. Numerous research studies claimed that the upregulation of various protein kinases (PKs) results in the development of metastatic breast cancers such as epidermal growth factor receptor (EGFR), vascular endothelial growth factor receptor (VEGFR), and platelet-derived growth factor receptor (PDGFR) [6–8].

**Fig. 1:**
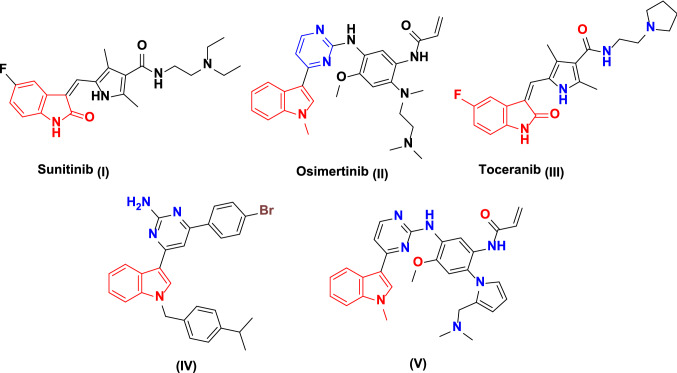
FDA-approved and other indole-based EGFR-TK inhibitors (I–V)

On the other hand, indole-based scaffolds have been shown to have significant potential to be used in numerous biological applications such as anticancer, antimicrobial, antiviral, antileishmanial, antitubercular, anti-oxidative, and analgesic effects [9, 10]. Moreover, the indole core is involved in many natural alkaloids that used as potent anticancer agents like vinblastine, vincristine, and vinorelbine [11–15]. Additionally, among the generic RTK (receptor tyrosine kinase) inhibitors in the market, sunitinib (**I**) and osimertinib (**II**) are indole-based anticancer drugs (Fig. 1) [16, 17].

Additionally, EGFR represents the furthermost critical member among the serine/threonine protein kinases. It promotes the propagation of signal transduction cascades responsible for cellular proliferation, migration, adhesion, angiogenesis, and apoptosis [18–20]. Therefore, genetic mutations affecting EGFR activity stand among the main causes of the progression of numerous human cancers [21–23]. In the last few years, there have been many EGFR TKIs (tyrosine kinase inhibitors) approved for the treatment of NSCLC (non-small cell lung cancer), including indole-based FDA-approved EGFR inhibitors such as torceranib (**III**) (Fig. 1) [24]. Notably, it was found that EGFR and/or its ligand TGF-α showed higher countenance levels that were predominantly assessed in most of the preclinical breast cancer cases and were characterized by bleak prognosis, higher resistance to various approved cytotoxic drugs (e.g., doxorubicin, 5-fluorouracil, vinblastine, and cisplatin), and being unresponsive to hormonal therapy [25]. For instance, indole-pyrimidine-based derivative **IV** displayed circumvention activities with IC_50_ values of 0.094, 0.099, and 0.595 µM against EGFR (T790M), EGFR (L858R), and c-MET, respectively (Fig. 1) [26]. Moreover, indol-3-acrylamide derivative **V** showed highly promising antiproliferative activities. It afforded a potent hampering activity with about 22-folds selectivity versus EGFR^L858R/T790M^ over EGFR^WT^ kinase with IC_50_ values of 1.7 and 37 nM, respectively (Fig. 1) [27].

**Fig. 2:**
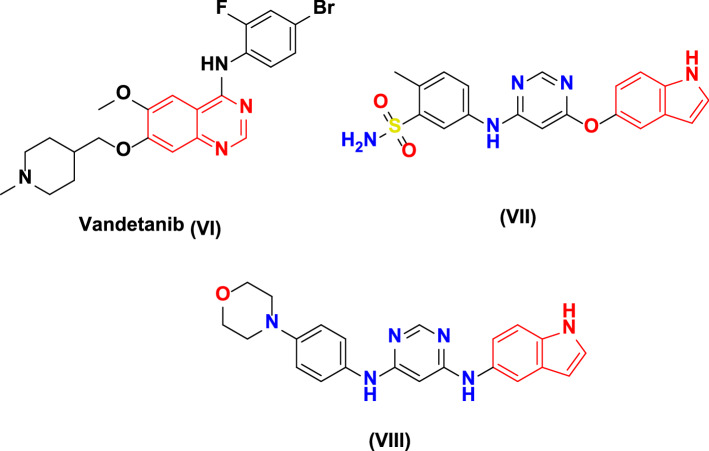
FDA-approved and other indole-based VEGFR-2-TK inhibitors (VI–VIII)

Moreover VEGF (vascular endothelial growth factor) is another RTK, including three isoforms of VEGF receptors, namely VEGFR-1 (Flt-1), VEGFR-2 (KDR), and VEGFR-3 (Flt-4) [28]. Midst these three main types of VEGF receptors, VEGFR-2 represents a reliable target for affording novel anticancer scaffolds due to its vital role in normal physiological and tumor pathophysiological angiogenesis [29, 30]. VEGFR-2 overexpression was already detected in versatile cancer types such as breast cancer and others [31–34]. Herein, many VEGFR-2 TKIs are clinically FDA-approved medicines [e.g., sunitinib (**I**)] (Fig. 1) [16, 17, 35]. Consequently, dual-VEGFR-2/EGFR circumvention in clinical studies is an ideal strategy for optimum anticancer activity. For example, vandetanib (**VI**) is a promising anticancer agent that acts as a dual-EGFR/VEGFR-2 inhibitor [36–38]. For instance, anilino-indole-based compound **VII** exhibited dual-hampering activities versus EGFR and VEGFR-2 with IC_50_ values of 18 and 45 nM, respectively (Fig. 2) [39]. Also, morpholino-indole-based compound **VIII** showed inhibitory activities against both EGFR and VEGFR-2 with IC_50_ values of 0.007 and 1.2 µM, respectively (Fig. 2) [39]. Briefly, an efficient dual-EGFR/VEGFR-2 antagonist should fulfill four fundamental pharmacophoric properties, including a hydrophobic head to fit into the first hydrophobic region in each receptor type, hydrogen bond-donating group as a spacer moiety, a heteroaromatic ring within the EFGR inhibitor’s structure to fit into the adenine-binding region, and a terminal hydrophobic tail to be inserted into the second hydrophobic region for EGFR antagonists' hinge region [40–42].

**Fig. 3:**
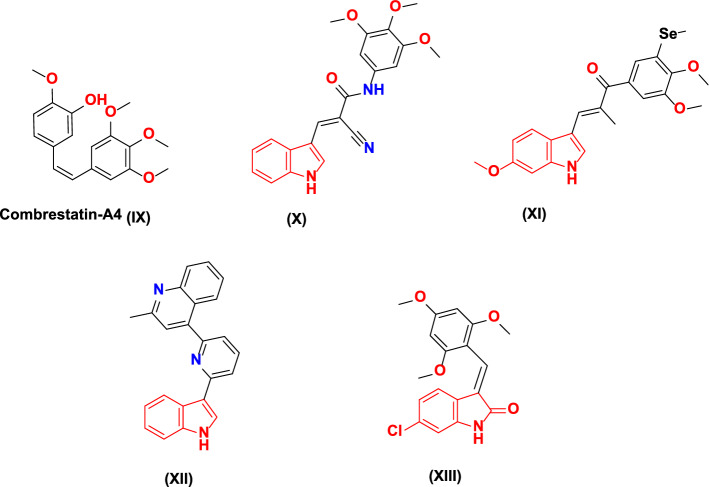
Tubulin polymerization inhibitors (IX-XII), other dual EGFR, and tubulin assembly antagonist biomolecule (XIII)

**Fig. 4:**
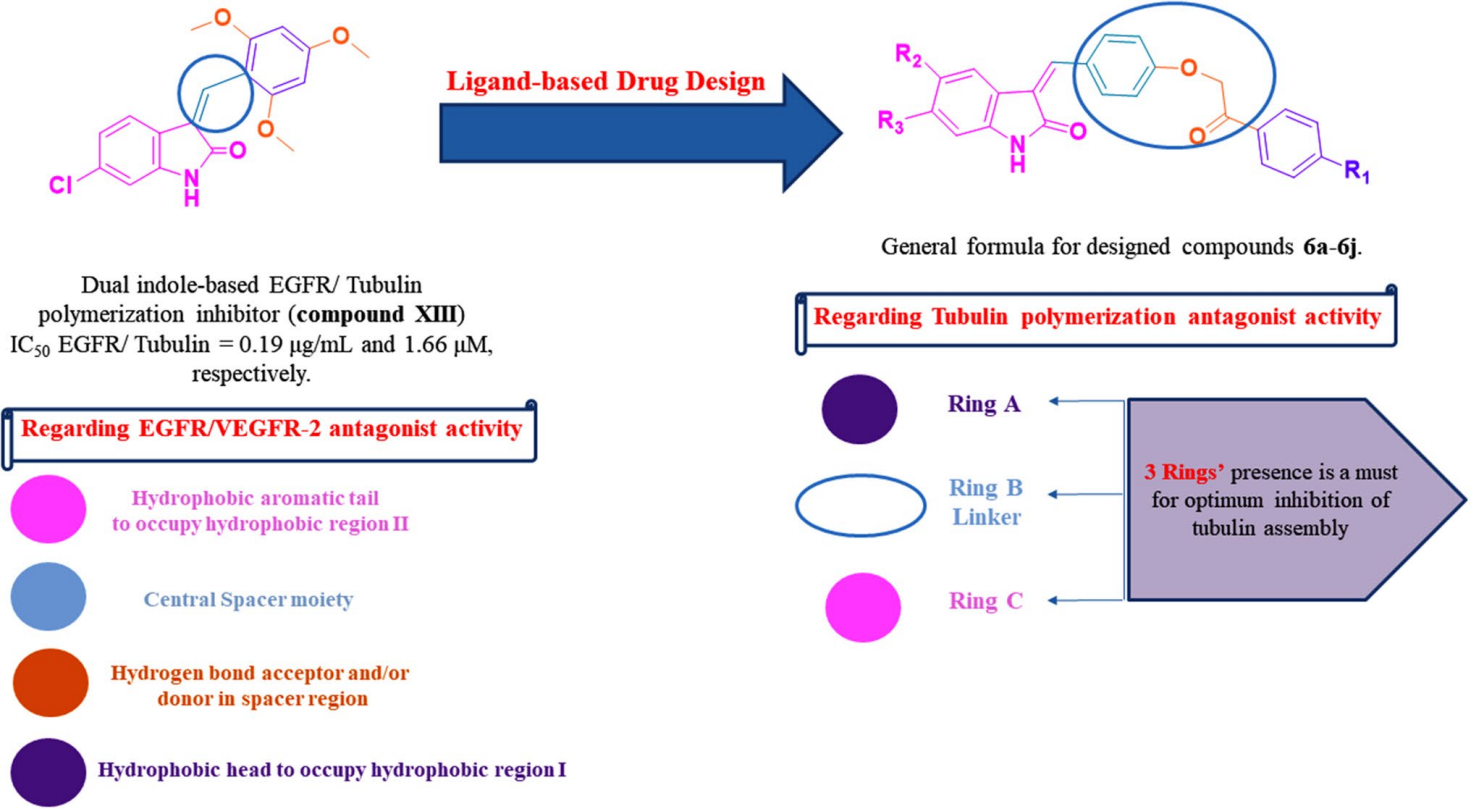
Rationale design of dual-EGFR/VEGFR-2 and tubulin polymerization inhibitors **6a–6j** as antiproliferative agents

On the other side, microtubules serve as a significant cytoskeleton component. It contributes many vital cellular functions, specifically the functionalization of the mitotic spindle during mitotic division and preserving the cell shape, intracellular transport, and movement [43, 44]. Their significance for cellular consistency and function reported in cancer chemotherapy makes microtubules a key milestone for affording reliable anticancer agents [45]. Moreover, microtubule targeting agents (MTAs) are classified into two kinds depending on their interference ability with microtubule polymerization and depolymerization, such as taxol and vincristine, respectively [46, 47]. The tubulin homology structure includes three diverse ligand binding sites: the paclitaxel (Taxol), vinblastine (vinca alkaloid), and colchicine-binding sites (CBS, e.g., combretastatin A4 (CA-4)) [48]. CA-4 (**IX)** (Fig. 3) is known to be an outstanding anticancer agent that has a selective targeting for cancer angiogenesis [11, 49–56]. Moreover, CA-4 and colchicine, as representative therapeutic agents of CBSIs (colchicine-binding site inhibitors), should contain three major parts; ring A, ring B (linker), and ring C [57, 58]. According to various previous literature data, these three rings of CBSIs must comprise seven key pharmacophoric properties, including three hydrogen-bond-accepting groups (A1, A2, and A3), one hydrogen-bond-donating group (D1), two hydrophobic cores (H1 and H2), and one flat group (R1) (Fig. 4) [59–61]. In relation, compound **X**, indole-based CA-4 analog, showed good antiproliferative activities against various cancer cell lines; also, it displayed acceptable anti-tubulin activity compared with positive control CA-4 with IC_50_ values of 18 and 0.54 µM, respectively (Fig. 3) [62]. Moreover, 3-substituted indole derivative **XI** exhibited a potent competitive inhibition at the colchicine-binding site, which is better than the positive control colchicine with IC_50_ of 1.30 and 2.93 µM, respectively [63]. Additionally, 3-substituted indole-quinoline scaffold **XII** displayed remarkable antiproliferative activities, hampered tubulin polymerization in a dose-dependent manner, and its IC_50_ was 5 µM which was twofolds higher than that of CA-4 (2.5 µM) [64].

Dual-EGFR/tubulin polymerization inhibition represents a unique chemotherapeutic technique to restrict the unlimited proliferation of versatile cancer cell types [65, 66]. Therefore, designing dual-EGFR/tubulin polymerization inhibitors as antiproliferative agents is an obvious and attractive approach. For instance, 2-oxindole-based scaffold **XIII** acts as an efficient dual-EGFR and tubulin assembly inhibitor with IC_50_ values of 0.19 μg/ml and 1.66 μM; compared with positive references, gefitinib and CA-4 with IC_50_ of 0.057 μg/ml and 0.42 µM, respectively (Fig. 3) [67].

## Rationale

Notably, designed compounds (**6a–j**) within this research work fulfills these previously mentioned four key pharmacophoric features to act as promising dual-EGFR/VEGFR-2 antagonists (oxindole ring acting as hydrophobic aromatic tail, benzylidene group acting as central spacer moiety, acetyl-oxy moiety acting as hydrogen bond acceptor and/or donor group, and substituted phenyl ring as hydrophobic aromatic head) (Fig. 4). Also, these scaffolds achieve the presence of three rings (**A**, **B**, and **C**) with the seven previously mentioned pharmacophoric requirements to act as efficient tubulin polymerization antagonists (substituted phenyl ring acting as ring A, phenoxy acetyl moiety as ring B linker, and oxindole ring as ring C) (Fig. 4). Therefore, based on these previously mentioned research findings, our new 3-substituted indole derivatives (**6a–j**) were designed and synthesized as dual-EGFR/VEGFR-2 and tubulin polymerization inhibitors to get optimum antiproliferative activity.

## Results and discussion

### Chemistry

**Scheme 1:**
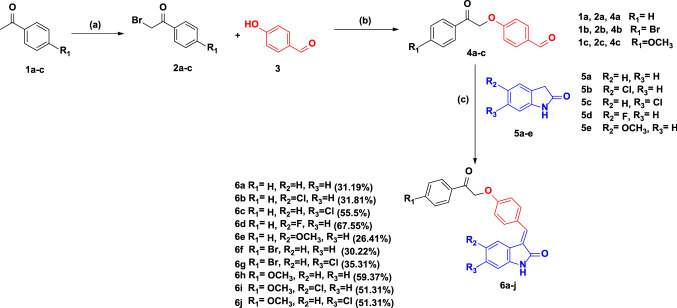
Synthesis of target oxindole derivatives **6a-j**. Reagents: a; *N*-bromosuccinamide, *p*-toluene sulfonic acid, Acetonitrile (ACN), reflux 24 h; b; K_2_CO_3_, ACN, reflux 24 h, c; Piperidine, EtOH, reflux 24 h

The outline for synthesizing target compounds **6a–j** is illustrated in Scheme 1. Alkylated *p*-hydroxy benzaldehyde derivatives **4a–c** were obtained via S_N_2 substitution reaction on various phenacyl bromide derivatives **2a–c** using *p*-hydroxy benzaldehyde's nucleophilic hydroxyl group. The alkylated *p*-hydroxy benzaldehyde derivatives **4ac** underwent a base-catalyzed Knoevenagel condensation reaction with the oxindole derivatives **5a-e** to afford compounds **6a–j** in a good yield [68]. NMR data supported the success of the used route. ^1^H NMR of all target compounds showed characteristic singlet signals at δ: 7.58–7.92 ppm for the benzylic protons; besides, NH protons were noticed as singlet signals at δ: 10.36–10.74 ppm. Additional singlet signals were also observed at δ: 5.63–5.77 ppm for the CH_2_ protons found in compounds **6a–j**.

Similarly, ^13^C NMR assured the formation of the target scaffolds showing characteristic carbonyl carbons of COCH_2_O groups at δ: 191.3–194.6 ppm, carbonyl carbons of CONH groups at δ: 167.1–169.3 ppm, and the aliphatic carbons (including COCH_2_O groups) at δ: 70.0–70.7 ppm. All the aromatic protons and carbons belonging to the oxindole, phenacyl, and phenyl scaffolds were reliable with the proposed structures of the compounds. The ESI/MS data of compounds **6a–j** appeared at their expected values via LC/MS.

Among the synthesized compounds **6a–j**, compounds** 6d** and** 6j** were obtained as mixtures of the *E* and *Z*-diastereomers in different ratios, as identified with their vinylic-proton and 2′ and 6′-benzylidene Hs chemical shifts (ppm) of *E* and *Z* in H NMR. The assignment of the *E*/*Z*-diastereomers was confirmed by ^1^H NMR, where the vinylic protons resonated slightly deshielded in the *Z*-diastereomer compared to the *E*-diastereomer due to the influence of the carbonyl group at position 2 of the oxindole ring. Additionally, 2′ and 6′ ortho-benzylidene protons were found to be more upfield shifted in the *E*-diastereomers for the same reason. For example, through the ^1^H NMR spectra of compound **6d**, the chemical shifts of the major *Z*-diastereomer revealed that the signals of vinylicH at 7.77 ppm, H_4_ of oxindole ring at 8.47 ppm, and H2′,6′ at 7.63–7.60 ppm, while those of the minor *E*-diastereomer resonated at 7.66, 8.41, and 7.40–7.36 ppm, respectively. We calculated the ratio of between E and Z-diastereomers for each compound depending on the total value of the integration of the vinylic-H in both for each compound. For instance, we suppose that the integration of the vinylic-H in both E and Z-diastereomers that is equal to 1 represents 100% of the existence of both E and Z-diastereomers, therefore, if the more upfield-shifted vinylic- H integration is equal to 0.3 that means the ratio of the E-diastereomer is equal to 30%, and also the more downfield-shifted vinylic-H integration is equal to 0.7 that means the ratio of the Z-diastereomer is equal to 70% [69].

### Biological investigation

#### Cytotoxic assays

##### One-dose assay of compounds 6a-j against sixty NCI cancer cell lines

All novel scaffolds **6a–j** were carefully selected by the National Cancer Institute (NCI), Bethesda, USA, to elucidate their cytotoxic activity versus 60 human cancer cell lines at 10 µM as a single-dose concentration after a 48 h incubation period. In relation, compounds **6c** and** 6h** showed moderate antiproliferative activity with cell growth inhibition % (GI %) of 41.17 and 43.66 against SNB-75 cells (CNS cancer cell line) and NCI/ADR-RES (ovarian cancer cell line), respectively. Moreover, compound **6i** exhibited remarkable antiproliferative activity with a GI% of 62.14 against HOP-92 (non-small cell lung cancer cell line). Regarding compound **6f**, it displayed highly promising antiproliferative activities versus UO-31, K-562, MOLT-4, SR, A549/ATCC, NCI-H23, SK-MEL-5, LOX IMVI, MCF-7, and T-47D cancer cell lines with GI% values of 51.52, 72.2, 91.46, 63.68, 41.65, 49.07, 71.12, 71.10, 72.61, and 53.05%, respectively. Furthermore, compound **6f** showed moderate to highly potent antiproliferative activities against all NCI leukemia cell lines with GI% values ranging from 41.78 to 91.46; besides, compound **6f** exhibited moderate to highly promising antiproliferative activities against all NCI breast cancer cell panels except MDA-MB-231/ATCC, and HS 578 T with GI%s ranging from 43.44 to 72.61. Additionally, compound **6g** also afforded remarkable antiproliferative activity against the same cell lines affected by compound **6f,** including UO-31, K-562, MOLT-4, SR, A549/ATCC, NCI-H23, SK-MEL-5, LOX IMVI, MCF-7, and T-47D cell lines with GI%s of 59.44, 58.26, 61.59, 59.72, 49.91, 46.84, 61.24, 45.75, 70.3, and 55.17, respectively. Also, compound **6g** showed selective moderate antiproliferative activities against all NCI leukemia cell lines with GI% values ranging from 34.26 to 61.59, and versus all NCI breast cancer cell panels except MDA-MB-231/ATCC and HS 578 T with GI%s ranging from 42.28 to 70.03. Besides, compound **6g** showed selective antiproliferative activity against ovarian NCI/ADR-RES cancer cell line with a GI% of 52.24. Hence, the NCI results indicate that among the tested compounds, compounds **6f** and **6g** are the most remarkable antiproliferative agents versus most of the tested NCI cancer cell panels (as shown in the supporting information).

**Fig. 5:**
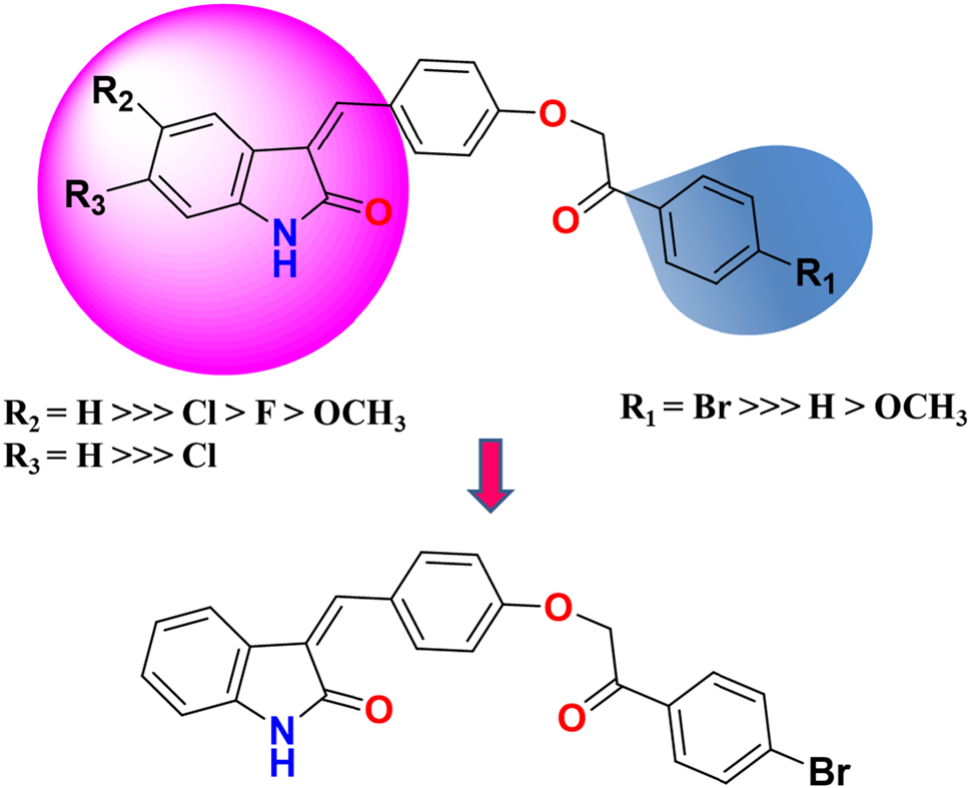
Structure–activity relationship of the designed compounds **6a–j** for more potent antiproliferative activity

Based on the results mentioned earlier about the NCI antiproliferative activities of compounds **6a–j**, we could conclude that (Fig. 5).

1. The presence of Br substitution at the para position of phenacyl moiety of the synthesized 3-substituted oxindole derivatives was favorable over the unsubstituted ring, which in turn was preferred over the methoxy group in the same position for affording potent antiproliferative activity against various NCI cancer cell lines, including MCF-7 (e.g., **6f**, and **6g**).
2. The unsubstituted oxindole ring was preferred over substituted ones.
3. Regarding the order of substitution on position, five of the oxindole ring was H > Cl > F > OCH_3_.
4. In relation, position six of the oxindole ring being unsubstituted was more preferred rather than being occupied by chlorine atom for optimum antiproliferative activity versus various NCI cancer cell lines, including MCF-7

##### Cytotoxicity versus MCF-7 cell line

Compounds **6f** and **6g** showed the uppermost observed antiproliferative activity versus the human breast cancer MCF-7 cell lines, so they were selected to detect the concentration required to hamper 50% of cellular growth (IC_50_) versus the MCF-7 cell lines compared to 5-fluorouracil **(**5FU**)** using the MTT assay [70]. Compound **6f** showed the highest considerable antiproliferative activity, specifically against MCF-7 cell lines, with a growth inhibition percentage (GI%) of 72.61%, affording an IC_50_ value of 14.77 μM; however, compound **6g** displayed a GI% value of 70.03%, exhibiting an IC_50_ value of 25.35 μM, compared to the reference standard 5FU with an IC_50_ of 2.02 μM (as shown in Table 1).

**Table 1:** The cytotoxicity of compounds **6f**, **6g**, and 5- fluorouracil (5FU) against Human Skin Fibroblasts (HSF) cells and MCF-7 cells using SRB-MTT assay

Compound	MCF-7	HSF	SI*
	(IC_50_ ± SD, uM)		
**6f**	14.77 ± 0.47	19.43 ± 1.17	1.31
**6g**	25.35 ± 1.23	nd	–
5FU	2.02 ± 0.17	5.45 ± 0.02	2.70

SI* = IC_50_ for HSF /IC_50_ for MCF-7 cells

– nd, not detected

##### Margin of safety assessment upon detection of cytotoxicity on human skin fibroblasts (HSF) cells

Depending on the outcome pointed out, compound **6f** displayed higher cytotoxicity than compound **6g** on MCF-7 cells. The safety profile of compound **6f** and the reference drug 5FU were investigated against MCF-7 and HSF cells (Human Skin Fibroblasts). This compound **6f** exhibited remarkable cancer selectivity (IC_50_ = 14.77 µM for MCF-7, IC_50_ = 19.45 µM for HSF; selectivity index (SI) = 1.31) comparable to the reference drug 5FU with selectivity index (SI) value of 1.70 (as shown in Table 1). These results showed that compound **6f** had a relatively good safety profile on the HSF normal cells than 5FU.

#### Cell cycle analysis

**Fig. 6:**
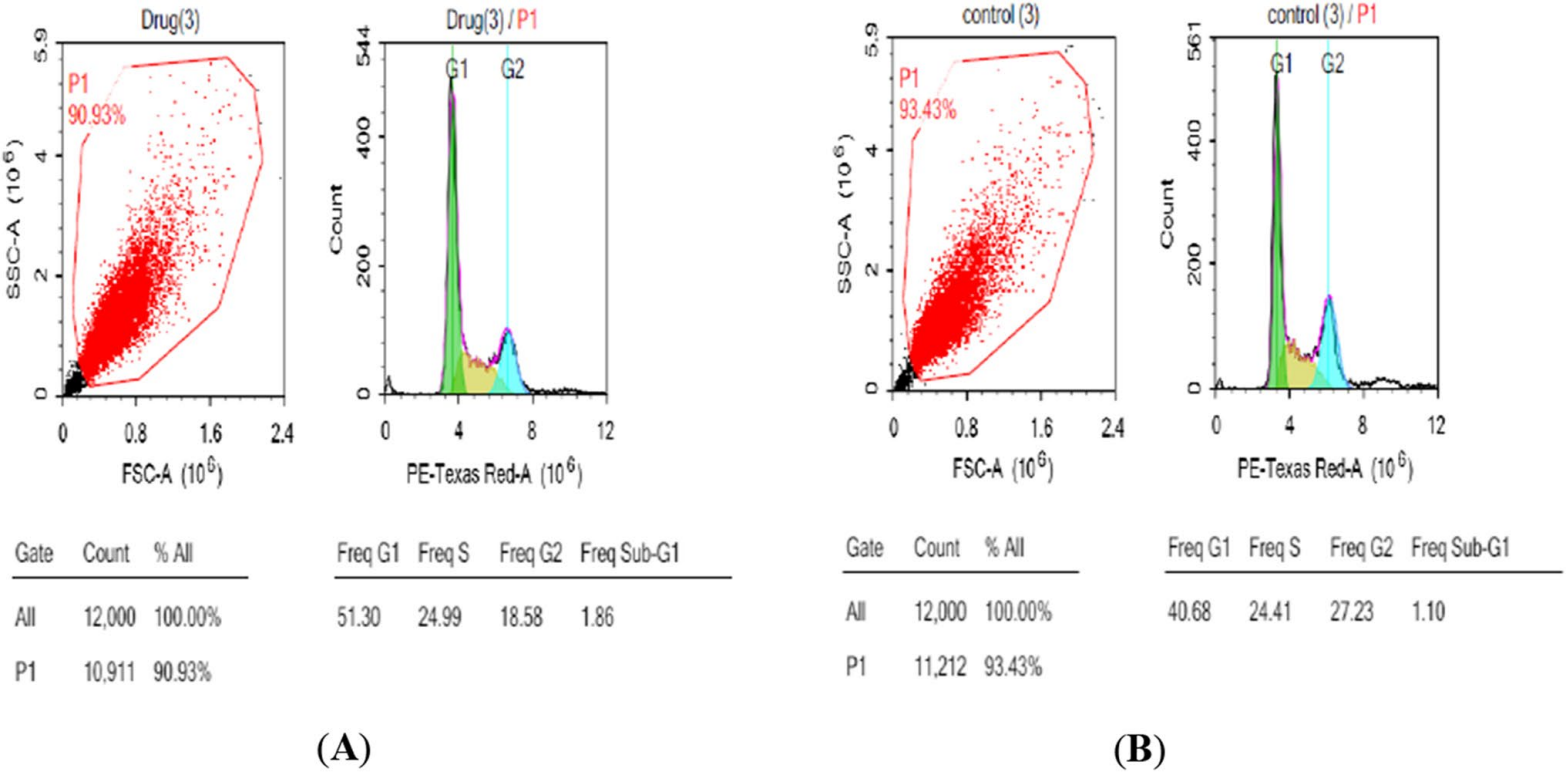
Cell cycle arrest analysis of **A** compound **6f** (cells treated with IC_50_ of compound **6f**), and **B** DMSO as control

To detect the possible mechanism displayed by compound **6f** to afford its antiproliferative activity, the effect of **6f** on cell cycle progression in MCF-7 cell lines was performed via DNA flow cytometric analysis. The obtained data for compound **6f** are shown in Fig. 6, displaying great cell cycle profile disruption compared with untreated MCF-7 cells. Treatment of MCF-7 cells with compound **6f** increased the percentage of accumulation of cells at Freq G1 (from 40.68 to 51.30%) combined with an increase in the proportion at the Freq S phase and a decrease in Freq G2/M phase, indicating that compound **6f** arrested cell cycle fundamentally at G0–G1phase.

#### RTK kinases’ circumvention

Due to the fundamental role of RTKs in cancer pathogenesis, we investigated the inhibitory efficacy of compounds** 6f** on three types of RTKs (EGFR, VEGFR-2 (KDR), and PDGFR_ẞ receptors using the kinase kit assay protocol [71]. The outcomes illustrated that compound **6f** fundamentally inhibited EGFR, VEGFR-2, and PDGFR_ẞ with an IC_50_ of 1.38 ± 0.008, 5.75 ± 0.011, and 3.18 ± 0.007 µM, respectively, compared to sunitinib as a reference (IC_50_ of 0.08 ± 0.005, 0.33 ± 0.006, and 0.18 ± 0.003 µM, respectively). These results, as mentioned above, demonstrated that compound **6f** principally circumvented EGFR with the lowest IC_50_ value detected for this scaffold; therefore, EGFR represents the most crucial target among the tested kinases’ series (as shown in Table 2).

**Table 2:** The inhibitory activities of compound **6f** versus EGFR, VEGFR-2, PDGFR_ẞ, and tubulin polymerization (IC_50_, uM)

Compound	Tubulin polymerization inhibition	EGFR inhibition	VEGFR-2 inhibition	PDGFR_ẞ inhibition
	(IC_50_ ± SD, uM)			
**6f**	7.99 ± 0.49	1.38 ± 0.008	5.75 ± 0.011	3.18 ± 0.007
Sunitinib	nd	0.08 ± 0.005	0.33 ± 0.006	0.18 ± 0.003
CA-4	2.64 ± 0.16	nd	nd	nd

– nd, not detected

#### Tubulin polymerization inhibition assays

To examine the tubulin polymerization hampering activity for the desired scaffolds and legitimize the design, the most potent compound, **6f**, was selected to investigate the circumvention of the tubulin polymerization process using combretastatin A4 (CA-4) as a positive control. The attained outcomes (as shown in Table 2) reflected that compound **6f** conserved tubulin polymerization inhibitory activity with an IC_50_ value of 7.99 ± 0.49 µM, compared to the reference CA-4 (IC_50_ = 2.64 ± 0.16 µM). Consequently, one of the proposed mechanisms for affording the antiproliferative activity of compound **6f** is the circumvention of tubulin Polymerization.

## In silico studies

The in silico docking study was carried out to explain the bindings modes of all synthesized compounds (**6a–j**) inside the binding sites of human VEGFR-2, EGFR, and tubulin (**PDB IDs: 1YWN**, **4HJO**, and **5LYJ,** respectively).

**Fig. 7:**
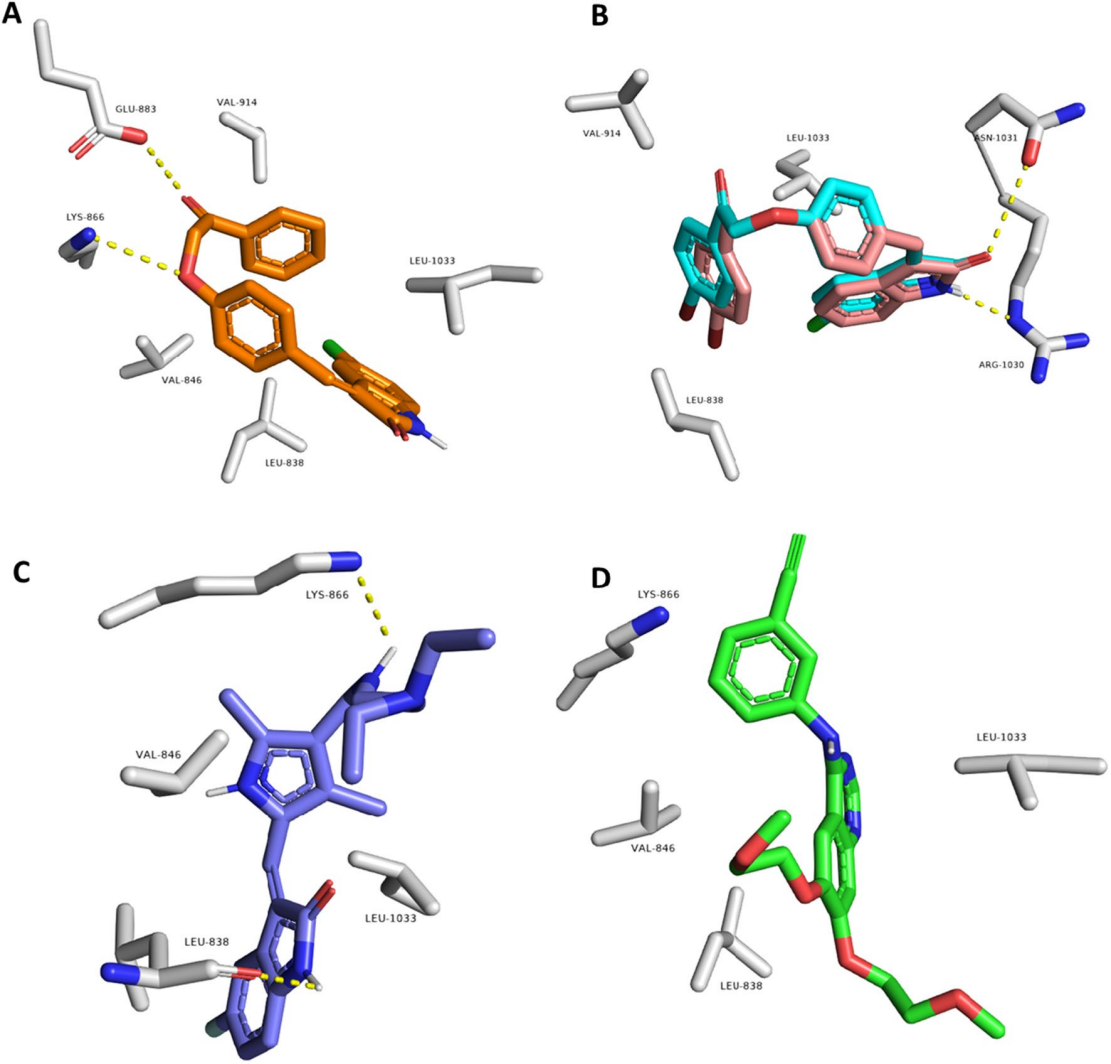
Binding modes of compounds **6b** (**A**), **6f**, and **6g** (**B**; brick red- and cyan-colored structures) together with two reference inhibitors, Sunitinib and Erlotinib (**C** and **D**, respectively) inside the binding site of the kinase domain of human VEGFR (**PDB ID: 1YWN**)

As shown in Table 3, compounds **6a–j** showed convergent binding modes with scores ranging from −7.6 to −8.9 kcal/mol upon fitting into the VEGFR-2’s binding site. Compounds **6a–e** showed the same binding interactions inside the VEGFR-2’s binding site, forming a common H-bond with Glu883 and Lys-866 in addition to two common hydrophobic interactions with Val-914 and Val-846. Compound **6b** was the best-scoring compound in this series and the whole set of compounds with a binding score of −8.9 kcal/mol (Fig. 7A). Compounds **6f–j** showed a different common binding mode, where they established two common H-bonds with Asn-1031 and Lys-866, along with three hydrophobic interactions with Leu-1033, Val-914, and Leu-838. Both **6f** and **6g** were the top-scoring compounds in this series with scores of -8.6 and -8.5 kcal/mol, respectively (Fig. 7B). Compound **6f** was the most active compound in the in vitro enzyme assay, and hence, its binding mode inside the VEGFR-2’s binding site might be the best binding mode in terms of stability and inhibitory activity. All the best-scoring compounds shared two hydrophobic interactions with the reference inhibitors (i.e., with Leu-1033 and Leu-838).

**Table 3:** Molecular modeling results for the synthesized compounds **6a-j**,** sunitinib,** and **erlotinib** at the active binding site of VEGFR-2 protein kinase, EGFR protein kinase, and tubulin enzyme (**PDB IDs: 1YWN**, **4HJO**, and **5LYJ**, respectively)

Compound	Docking Score ΔG values kcal/mol versus VEGFR-2 kinase	Docking Score ΔG values kcal/mol versus EGFR kinase	Docking Score ΔG values kcal/mol versus tubulin enzyme
**6a**	−7.6	−8.2	−7.6
**6b**	−8.9	−8.8	−7.9
**6c**	−8.3	−8.5	−7.1
**6d**	−8.2	−8.0	−7.3
**6e**	−8.1	−8.1	−7.6
**6f**	−8.6	−10.6	−10.2
**6g**	−8.5	−10.1	−8.9
**6h**	−8.0	−9.3	−9.5
**6i**	−8.1	−8.6	−9.1
**6j**	−7.8	−8.8	−9.6
Sunitinib	−8.5	−8.5	–
Erlotinib	−7.5	−7.5	–
Combretastatin A4	–	–	−11.3

**Fig. 8:**
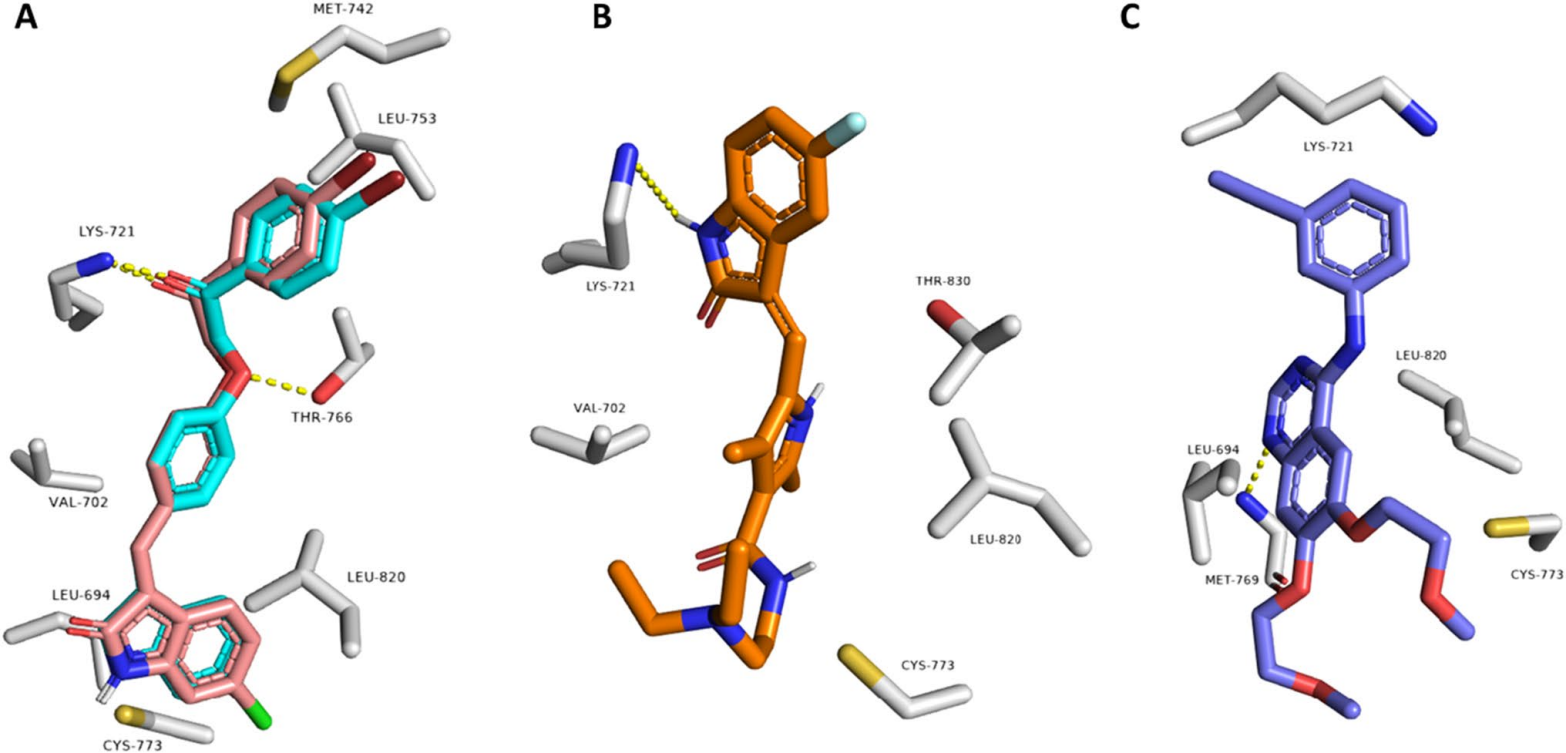
Binding modes of compounds **6f** and **6g** (**A**; brick red- and cyan-colored structures, respectively) together with two reference inhibitors, Sunitinib and Erlotinib (**B** and **C**, respectively) inside the binding site of the kinase domain of human EGFR (**PDB ID: 4HJO**)

Regarding EGFR, compounds **6a–j** showed comparable binding modes inside the enzyme’s binding site, with docking scores ranging from −8.0 to −10.6 kcal/mol (Table 3). Compounds **6a–e** exhibited almost the same binding modes and interactions, where they established several hydrophobic interactions with Leu-834, Leu-820, Lys-721, and Val-702. The remaining compounds, **6f–j**, showed a slightly different orientation making most of them able to form two H-bonds with Thr-766 and Lys-721, particularly compounds **6f** and **6g** (Fig. 8A). In addition, they established five common hydrophobic interactions with Leu-820, Cys-773, Leu-753, Met-742, and Val-702. The co-crystalized inhibitor (i.e., Erlotinib) together with another reference inhibitor (i.e., sunitinib) was able to establish the same hydrophobic interactions along with single H-bond with either Met-769 or Lys-721 (Fig. 8B–C). It was reported that lapatinib interacted with the inactive kinase due to the bulkiness of [(3-fluorobenzyl)oxy] moiety on its aniline side ring. This bulky moiety could not interact with the small-sized ATP-binding pocket, so it became able to interact with the allosteric-binding site, which opened through the displacement of the αC helix in the inactive conformation [72]. Similarly, the bulkiness of the phenacyl-phenoxy moiety of compound **6f** was unable to fit inside a narrow small-sized ATP-binding site; therefore, compound **6f** interacted selectively with the inactive EGFR conformation but not with the active conformation. As a result, compound **6f** was the most active derivative against EGFR among the synthesized series of compounds, indicating that its binding mode, which was convergent to the reference inhibitors, is the most enzyme inhibition-relevant one [73].

**Fig. 9:**
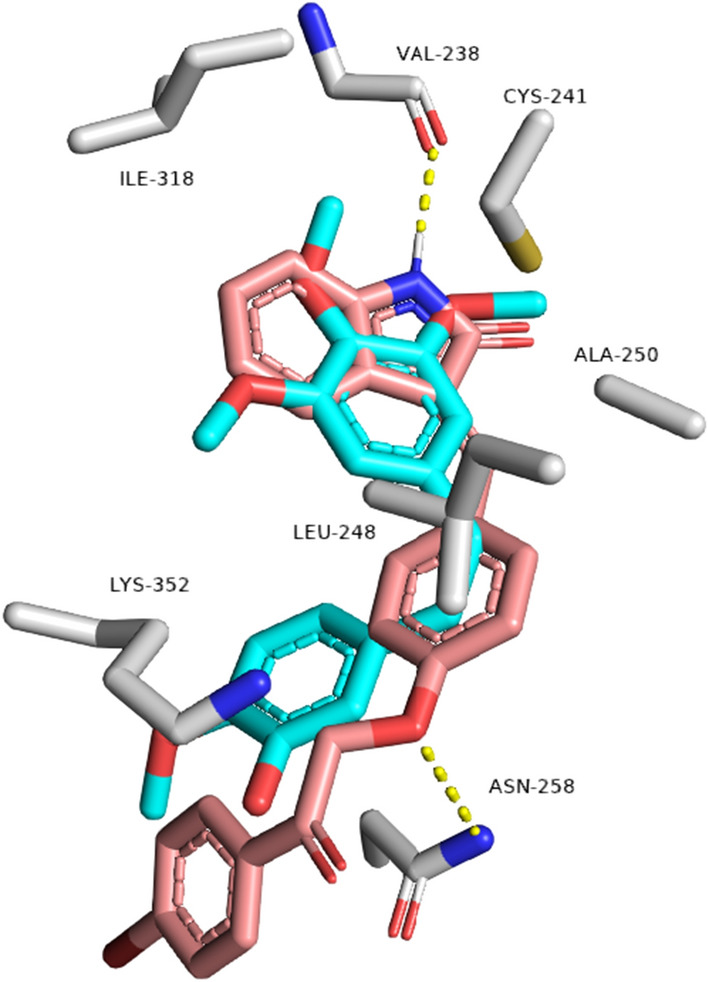
Binding modes of compounds **6f** (brick red-colored structure) together with the reference inhibitor, Combretastatin A4 (cyan-colored structure) inside the binding site of human tubulin (**PDB ID: 5LYJ**)

Finally, docking of compounds **6a–j** inside the tubulin’s colchicine-binding site showed that compound **6f** was the only derivative that was able to perfectly align with the co-crystalized inhibitor Combretastatin A4 with the highest docking score (-10.2 kcal/mol; Table 3) sharing the same hydrophobic interactions, in addition, two extra H-bonds with Asn-258 and Val-238 (Fig. 9). The binding mode of compound **6f** explains its superior in vitro tubulin polymerization inhibition in comparison with the other derivatives.

### Physicochemical, ADME, and pharmacokinetic characteristics forecast

**Fig. 10:**
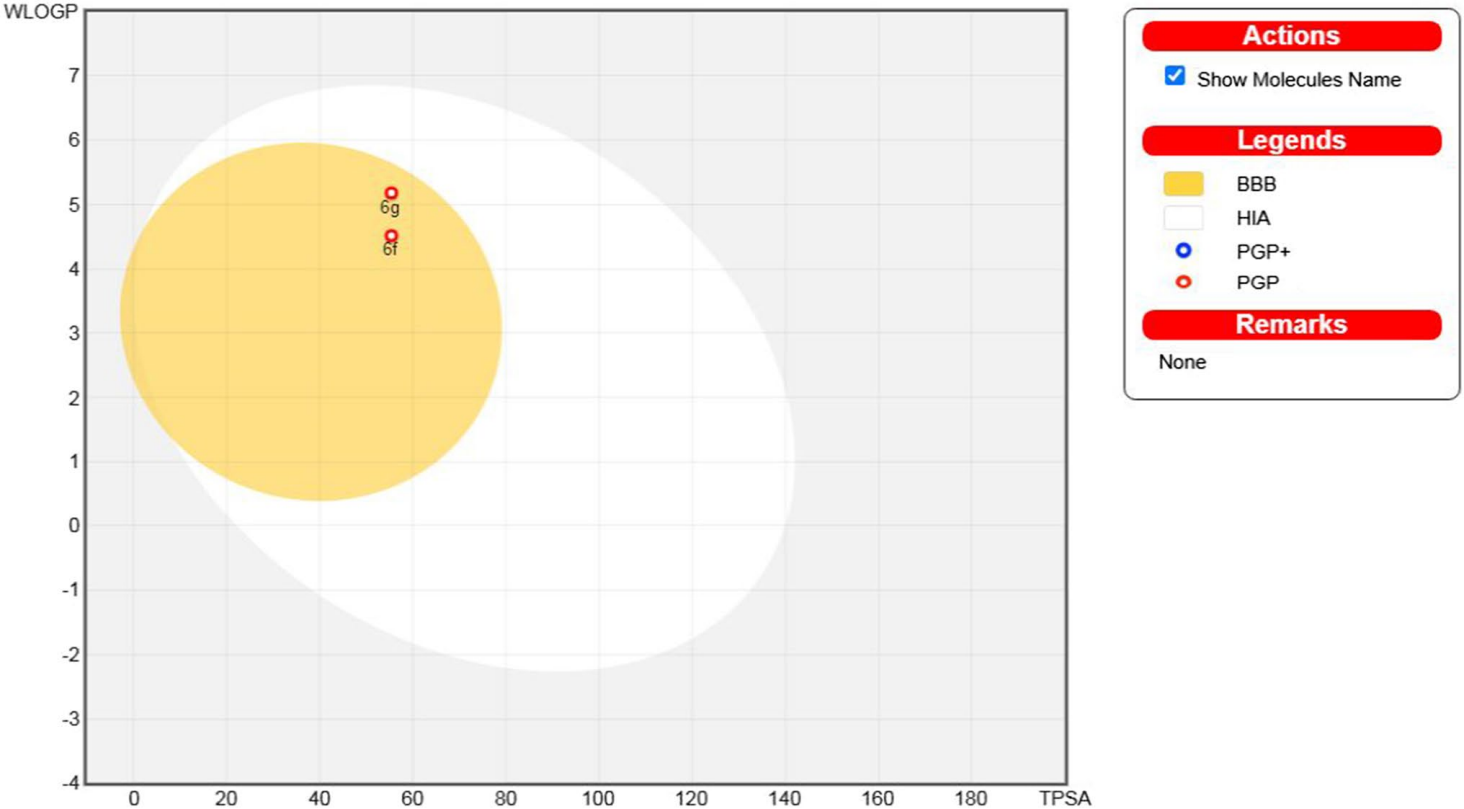
Forecast Boiled-Egg plotting from Swiss ADME online website for scaffolds (**6f** and **6g**)

The purchasing free easily entered Swiss ADME website supplied by the Swiss Institute of Bioinformatics (SIB) stands among the furthermost practical computational approaches to offer a worldwide assessment of the pharmacokinetics characterization and prospects of the drug-likeness property of small scaffolds [74]. This was carried out to ensure that the novel synthesized scaffolds are hopeful members of physiological potency and pharmacokinetic properties. The most active antiproliferative 3-substituted oxindole-based compounds (**6f** and **6g**) revealed a predicted logP_o/w_ value of 4.52 and 5.18, respectively, with good hydrophilicity and good GIT absorption and blood–brain barrier (BBB) penetration. Figure 10 demonstrates a BOILED-Egg graph of the WLOGP vs. TPSA (Topological Polar Surface Area) for the submitted scaffolds **6f** and **6g** [75]. The submitted scaffolds are placed with BBB permeability in the human intestinal absorption (HIA) sector. Furthermore, this diagram illustrates that the installed scaffolds were not P-glycoprotein substrates (PGP-) and not susceptible to the efflux framework influenced by like this kind of transporters, which is applied by many carcinogenic cell lines as an impedance technique.

**Fig. 11:**
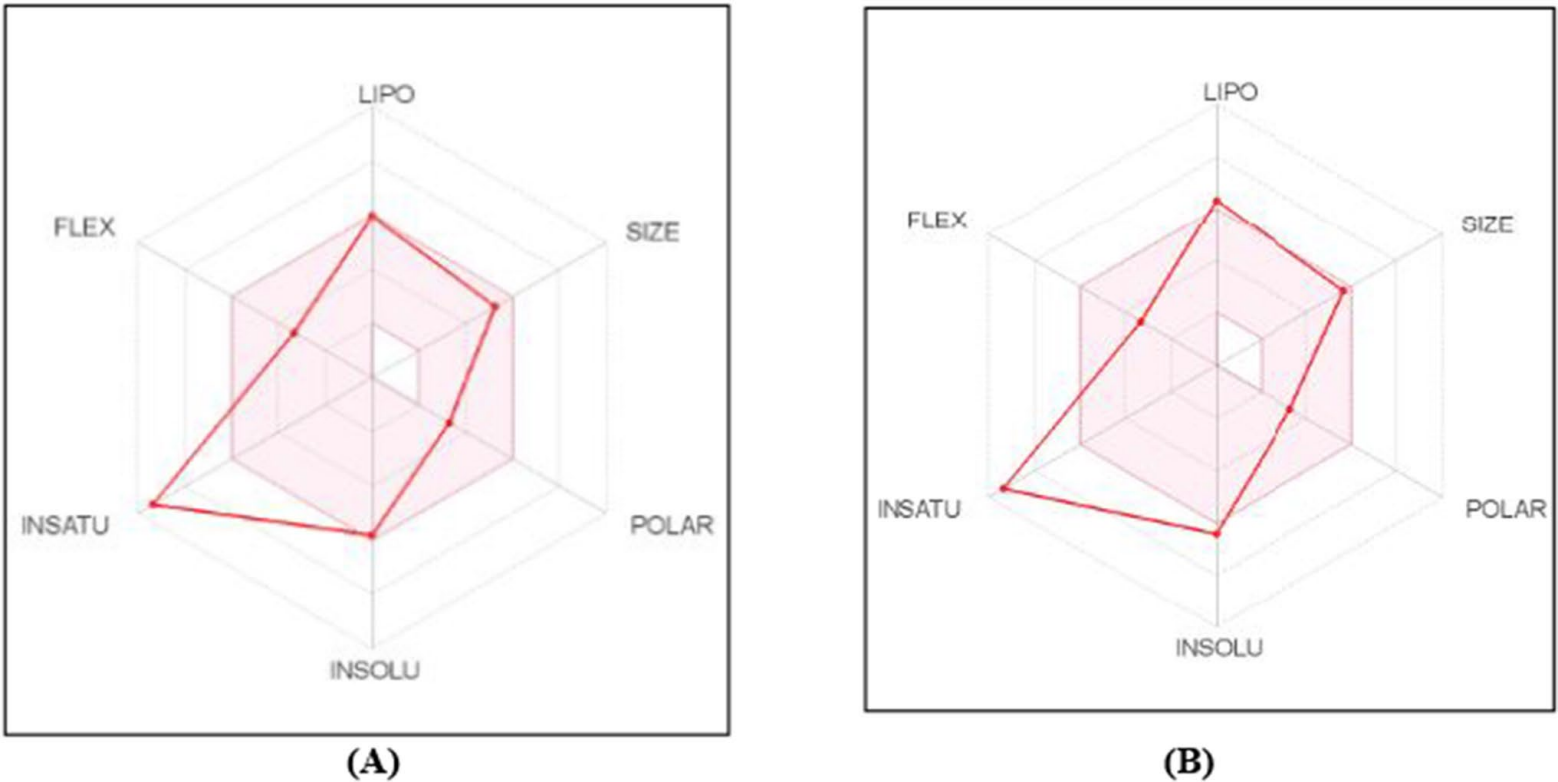
Radar bioavailability diagram from Swiss ADME website for scaffolds **6f** (**A**) and **6g** (**B**). The pink area characterizes the optimum property values range for the oral bioavailability, and the red lines represent the forecasted characters of **6f** and **6g**

The prophesied good GIT absorption of the installed scaffolds **6f** and **6g** is owing to their idyllic physicochemical characteristics positioned within the appropriate physicochemical characters area for oral bioavailability. Figure 11 displays the bioavailability radar chart for scaffolds **6f** and** 6g.** The radar plotting consists of six axes for six vital properties of oral bioavailability: saturation (INSATU), flexibility (FLEX), lipophilicity (LIPO), size (SIZE), polarity (POLAR), and solubility (INSOLU) [75]. The pink area is considered the range of the ideal property values. The red lines represent scaffolds **6f** (Fig. 11A) and **6g** (Fig. 11B), whose forecasted properties are almost entirely comprised in the pink area, demonstrating their good prophesied oral bioavailability. The Swiss ADME online website exhibited that the 3-substituted oxindole-based scaffolds **6f** and **6g** fulfill drug-resemblance properties well-defined by the top pharmaceutical companies: Lipinski's (Pfizer) [76], Ghose’s (Amgen) [77], and Veber’s (GSK) filters [78]. This signposts that the novel synthesized scaffolds own auspicious pharmacokinetic characters and respectable drug-resemblance characteristics.

## Conclusions

The current research work was employed to synthesize a novel series of 3-substituted oxindole derivatives **6a–j** as antiproliferative agents aiming to target multiple cellular targets concurrently. The newly synthesized hybrids **6a–j** exhibited promising antiproliferative activities against a panel of sixty NCI cancer cell lines. Compounds; **6f** and **6g** were the most potent antiproliferative agents against most of the NCI-tested cancer cell panels. Moreover, **6f** and **6g** showed remarkable antiproliferative activities against MCF-7 cell lines with GI% equal to 70.30% and 71.12%, respectively. Critically, it was found that compound **6f** more potent than **6g** in MTT assay against MCF-7. **6f** can trigger cytotoxicity versus the MCF-7 cell line with an IC_50_ value of 14.77 µM. This cytotoxic activity is accompanied by its ability to potently circumvent various tyrosine kinases with good IC_50_ values, including EGFR, VEGFR-2, and PDGFR-ẞ. Additionally, compound **6f** can circumvent tubulin polymerization with an IC_50_ value of 7.99 µM compared with CA-4 with an IC_50_ value of 2.63 µM, resulting in a mitotic division disturbance. Moreover, via the cell cycle arrest analysis, it was detected that compound **6f** could trigger cell cycle arrest at the G0–G1phase. The molecular-docking simulation into the active site of VEGFR-2 revealed that the newly designed hybrids **6a–j** showed higher binding free energies toward VEGFR-2 enzyme than the native ligand, erlotinib, and the reference sunitinib. In relation, fitting into the EGFR-binding site revealed that the newly designed hybrids **6a–j** displayed higher binding free energies toward EGFR enzyme than the native ligand, erlotinib, and the reference sunitinib, depicting the most active compounds, **6f**, and **6g**. Eventually, fitting into the tubulin-binding site elucidated that compound **6f** had the highest binding energy score among the synthesized compounds toward tubulin enzyme compared to the reference combretastatin **A4**, which was in good agreement with the in vitro results. Based on the biological data, the acceptable physicochemical and pharmacokinetic characteristics, and the good agreement with the molecular-docking study results propose that compound **6f** can be utilized as an efficient lead compound in designing and developing novel therapeutic agents for fighting MCF-7 breast cancer.

## Materials and methods

### Chemistry

All chemicals, including solid compounds and organic solvents utilized to produce the desired scaffolds, were of the commercial mark and bought from Sigma Aldrich, Alfa Aesar, and Pio-chem companies for pharmaceutical and chemical materials and handled without additional purification. Reaction monitoring was carried out using precoated TLC plates (Kiesslgel 60 F254, Merck), and spot detection was performed via UV lamb at 254 nm as a wavelength value. Measurement of melting points was carried out on Stuart Electro-Thermal apparatus and recorded without further correction. ^1^HNMR was done on a JOEL JNM-ECA400 (^1^H: 400 MHz) spectrophotometer at Zagazig University, Egypt. ^13^CNMR has performed on a JOEL JNMECA400 (^13^C: 100 MHz) spectrophotometer at Zagazig University, Egypt. Chemical shifts were measured in parts per million (ppm) relative to standard TMS using DMSO (δ: 2.5) as a deuterated solvent, and the coupling constant (J) was measured in Hertz (Hz). Multiplicity was nominated as s singlet; d doublet; t triplet; q quartet; p pentet; dd doublet of doublet; and m for multiplet. Elemental micro-analysis was performed at the regional center for mycology and biotechnology, Al-Azhar University, Cairo, Egypt. LC/MS data were carried out on the LC/MS/MS (Agilent 1260 Infinity II (USA) with 6420 Triple Quad LC/MS detector) at Nawah, Mokatam, Egypt.

#### General procedures for the synthesis of target compounds 2a–c

A mixture of acetophenone or acetophenone derivatives (1 mmol), *N*-bromo succinimide (1 mmol), and *p*-toluene sulfonic acid (1 mmol) in acetonitrile (50 ml) were refluxed for 24 h. The solvent was removed under reduced pressure. The obtained residue was recrystallized from acetonitrile to afford the target compounds **2a–c**.

- **2-bromo-1-phenylethan-1-one (2a)**
- White crystals; 0.15 g, 75.53% yield; mp: 49–51 °C; (Reported 50 °C) [79].
- **2-bromo-1-(4-bromophenyl) ethan-1-one (2b)**
- White powder; 0.2 g, 72.20% yield; m.p:111 °C; (Reported 108–110 °C) [80].
- **2-bromo-1-(4-methoxyphenyl) ethan-1-one (2c)**
- Yellow crystals; 0.23 g, 76.66% yield; mp: 68–71 °C; (Reported 69–72 °C) [81].

#### General procedures for the synthesis of target compounds 4a–c

A mixture of phenacyl bromide (1 mmol), *p*-hydroxy benzaldehyde (122 mg, 1 mmol), and potassium carbonate (276 mg, 2 mmol) in acetonitrile (50 ml) was refluxed for 24 h. The reaction mixture is cooled to room temperature, diluted with water, and then extracted three times with ethyl acetate. After drying the organic layer with anhydrous MgSO_4_, the product was obtained as yellow powder via evaporation of the ethyl acetate layer. The obtained residue was recrystallized from acetonitrile to afford the target compounds **4a–c** [82, 83].

- **4-(2-Oxo-2-phenylethoxy) benzaldehyde (4a)**
- Yellow crystals; 0.11 g, 22.64% yield; mp: 118 °C; (Reported 120 °C) [82, 83].
- **4-(2-(4-Bromophenyl)-2-oxoethoxy) benzaldehyde (4b)**
- Yellowish white crystals; 0.11 g, 62.3% yield; mp: 97–99 °C; (Reported 98 °C) [84].
- **4-(2-(4-Methoxyphenyl)-2-oxoethoxy) benzaldehyde (4c)**
- White crystals; 0.11 g, 22.64% yield; mp: 83–85 °C; (Reported 80–85 °C) [82, 83].

#### General procedures for the synthesis of target compounds 6a–j

A mixture of compounds **4a–c** (1 mmol) and 2-oxindole or oxindole derivatives **5a–e** (1 mmol) in absolute ethanol (50 ml) and piperidine (85 mg, 1 mmol) was refluxed for 24 h. Compounds **6a–j** were formed on hot, filtrated, washed several times with hot ethanol, and dried in a vacuum oven to afford compounds **6a–j** [68].

##### 3-{4-[2-Oxo-2-phenylethoxy] benzylidene}indolin-2-one (6a)

Yellow powder; 0.14 g, 31.19% yield; mp: 219–221 °C; *R*_f_ = 0.33 (Pet.ether/ethyl acetate, 6:2.5); ^1^H NMR (400 MHz, DMSO-*d*_6_) δ 10.55 (s, 1H, N*H*), 8.04 (d, *J* = 8.5 Hz, 2H, Ar*H*), 7.70 (d, *J* = 8.5 Hz, 3H, Ar*H*), 7.65 (d, *J* = 7.8 Hz, 1H, Ar*H*), 7.60 (s, 1H, Ar*H*), 7.58 (d, *J* = 4.5 Hz, 2H, Ar*H*), 7.21 (t, *J* = 8.1 Hz, 1H, Ar*H*), 7.12 (d, *J* = 8.8 Hz, 2H, Ar*H*), 6.89–6.85 (m, 2H, Ar*H*), 5.71 (s, 2H, CO-C*H*_*2*_*-*O); ^13^C NMR (100 MHz, DMSO-*d*_6_) δ 194.24, 168.91, 159.27, 142.70, 135.92, 134.31, 133.91, 131.43, 129.76, 128.88, 127.92, 126.99, 125.73, 122.10, 121.14, 114.98, 114.47, 110.07, 70.26; Anal. Calcd. for C_23_H_17_NO_3_ (355.12): C, 77.73; H, 4.82; N, 3.94, Found: C, 77.72; H, 4.84; N, 3.95; ESI/MS: m/z Calcd. For [M + Na] ^+^: 378.12, Found: 377.9.

##### 5-Chloro-3-{4-[2-oxo-2 phenylethoxy]benzylidene}indolin-2-one (6b)

Yellow powder; 0.13 g, 31.81% yield; mp: 189–191 °C; *R*_f_ = 0.32 (Pet.ether/ethyl acetate, 6:2.5); ^1^H NMR (400 MHz, DMSO-*d*_6_) δ 10.71 (s, 1H, N*H*), 8.05 (d, *J* = 7.3 Hz, 2H, Ar*H*), 7.91–7.84 (m, 1H, Ar*H*), 7.72–7.66 (m, 3H, Ar*H*), 7.64–7.53 (m, 3H, Ar*H*), 7.28 (dd, *J* = 8.3, 2.0 Hz, 1H, Ar*H*), 7.17 (d, *J* = 8.7 Hz, 2H, Ar*H*), 6.89 (d, *J* = 8.3 Hz, 1H, Ar*H*), 5.73 (s, 2H, CO-C*H*_*2*_*-*O); ^13^C NMR (100 MHz, DMSO-*d*_6_) δ 194.50, 167.56, 160.70, 141.86, 138.29, 135.06, 134.30, 131.94, 129.27, 128.32, 127.73, 126.97, 125.71, 123.50, 119.76, 115.50, 114.96, 110.96, 70.62; Anal. Calcd. for C_23_H_16_ClNO_3_ (389.08): C, 70.86; H, 4.14, N, 3.59, Found: C, 70.87; H, 4.15; N, 3.61; ESI/MS: m/z Calcd. For [M + Na] ^+^: 412.08, Found: 411.8.

##### 6-Chloro-3-{4-[2-oxo-2-phenylethoxy]benzylidene}indolin-2-one (6c)

Dark yellow powder; 0.23 g, 55.5% yield; mp: 199–201 °C; *R*_f_ = 0.30 (Pet.ether/ethyl acetate, 6:2.5); ^1^H NMR (400 MHz, DMSO-*d*_6_) δ 10.72 (s, 1H, N*H*), 8.05 (d, *J* = 8.5 Hz, 2H, Ar*H*), 7.70 (d, *J* = 8.5 Hz, 3H, Ar*H*), 7.65 (s, 1H, Ar*H*), 7.63–7.59 (m, 3H, Ar*H*), 7.16–7.09 (m, 2H, Ar*H*), 6.94–6.88 (m, 2H, Ar*H*), 5.72 (s, 2H, CO-C*H*_*2*_*-*O); ^13^C NMR (100 MHz, DMSO-*d*_6_) δ 194.20, 168.87, 159.49, 144.01, 141.36, 137.88, 136.86, 134.46, 133.92, 131.59, 127.92, 126.72, 124.54, 123.32, 120.81, 120.10, 115.08, 109.99, 70.27; Anal. Calcd. for C_23_H_16_ClNO_3_ (389.08): C, 70.86; H, 4.14, N, 3.59, Found: C, 70.88; H, 4.15; N, 3.62; ESI/MS: m/z Calcd. For [M-H]^−^: 388.08, Found: 388.09.

##### (*E*/*Z*)-5-Fluoro-3-{4-[2-oxo-2-phenylethoxy]benzylidene}indolin-2-one (6d). *E*:* Z* ratio = 40: 60

Yellow powder; 0.33 g, 67.55% yield; mp: 223–226 °C; *R*_f_ = 0.28 (Petr.ether/ethyl acetate, 6:2.5);

*Z*-Diastereomer: ^1^H NMR (400 MHz, DMSO-*d*_6_) δ 10.59 (s, 1H, N*H*), 8.47 (d, *J* = 9 Hz, 1H, Ar*H*), 8.05 (t, *J* = 7.1 Hz, 1H, Ar*H*), 7.77 (s, 1H, Ar*H*), 7.71 (d, *J* = 7.3 Hz, 1H, Ar*H*), 7.63 – 7.60 (m, 2H, Ar*H*), 7.58 (d, *J* = 8.0 Hz, 1H, Ar*H*), 7.22–7.14 (m, 2H, Ar*H*), 7.04 (dd, *J* = 8.3, 2.1 Hz, 1H, Ar*H*), 6.93 (d, *J* = 8.7 Hz, 1H, Ar*H*), 6.88 (d, *J* = 3.5 Hz, 1H, Ar*H*), 6.86 (d, *J* = 3.4 Hz, 1H, Ar*H*), 5.77 (s, 2H, CO-C*H*_*2*_*-*O) ppm; ^13^C NMR (100 MHz, DMSO-*d*_6_) δ 191.31, 167.41, 159.56, 138.46, 137.63, 136.47, 134.56, 134.30, 133.88, 131.52, 127.91, 127.10, 126.57, 123.84, 115.85, 115.10, 114.53, 109.84, 70.20 ppm.

*E*-Diastereomer: ^1^H NMR (400 MHz, DMSO-*d*_6_) δ 10.55 (s, 1H, N*H*), 8.41 (d, *J* = 9 Hz, 1H, Ar*H*), 7.85 (t, *J* = 7.1 Hz, 1H, Ar*H*), 7.66 (s, 1H, Ar*H*), 7.41 (d, *J* = 7.3 Hz, 1H, Ar*H*), 7.40 – 7.36 (m, 2H, Ar*H*), 7.35 (d, *J* = 8.0 Hz, 1H, Ar*H*), 7.10–7.04 (m, 2H, Ar*H*), 6.99 (dd, *J* = 8.3, 2.1 Hz, 1H, Ar*H*), 6.80 (d, *J* = 8.7 Hz, 1H, Ar*H*), 6.78 (d, *J* = 3.5 Hz, 1H, Ar*H*), 6.76 (d, *J* = 3.4 Hz, 1H, Ar*H*), 5.73 (s, 2H, CO-C*H*_*2*_*-*O) ppm; ^13^C NMR (100 MHz, DMSO-*d*_6_) δ 194.19, 168.87, 160.22, 139.01, 137.63, 136.47, 134.56, 133.88, 131.69, 130.02, 128.86, 127.10, 126.59, 123.87, 116.08, 115.19, 114.62, 109.92, 70.27 ppm.

Anal. Calcd. for C_23_H_16_FNO_3_ (373.11): C, 73.99; H, 4.32; N, 3.75, Found: C, 74.00; H, 4.34; N, 3.76; ESI/MS: m/z Calcd. For [M + Na] ^+^: 396.11, Found: 395.8.

##### 5-Methoxy-3-{4-[2-oxo-2-phenylethoxy]benzylidene}indolin-2-one (6e)

Yellow powder; 0.01 g, 26.41% yield; mp: 196–198 °C; *R*_f_ = 0.34 (Pet.ether/ethyl acetate, 6:2.5); ^1^H NMR (400 MHz, DMSO-*d*_6_) δ 10.36 (s, 1H, N*H*), 8.05 (d, *J* = 7.3 Hz, 2H, Ar*H*), 7.69 (d, *J* = 8.8 Hz, 3H, Ar*H*), 7.61 (s, 1H, Ar*H*), 7.58 (d, *J* = 5.7 Hz, 2H, Ar*H*), 7.25–7.18 (m, 1H, Ar*H*), 7.14 (d, *J* = 8.8 Hz, 2H, Ar*H*), 6.83 (dd, *J* = 8.5, 2.2 Hz, 1H, Ar*H*), 6.78 (d, *J* = 8.5 Hz, 1H, Ar*H*), 5.71 (s, 2H, CO-C*H*_*2*_*-*O), 3.64 (s, 3H, OC*H*_*3*_); ^13^C NMR (100 MHz, DMSO-*d*_6_) δ 194.26, 168.94, 159.31, 154.03, 136.97, 136.20, 134.33, 133.92, 131.34, 128.90, 126.88, 126.33, 124.75, 121.95, 114.95, 114.45, 109.73, 108.72, 70.25, 55.34; Anal. Calcd. for C_24_H_19_NO_4_ (385.13): C, 74.79; H, 4.97; N, 3.63, Found: C, 74.80; H, 4.99; N, 3.64; ESI/MS: m/z Calcd. For [M + Na] ^+^: 408.13, Found: 407.8.

##### 3-{4-[2-(4-Bromophenyl)-2-oxoethoxy]benzylidene}indolin-2-one (6f)

Yellow powder; 0.13 g, 30.22% yield; mp: 265–267 °C; *R*_f_ = 0.32 (Pet.ether/ethyl acetate, 6:2.5);^1^H NMR (400 MHz, DMSO-*d*_6_) δ 10.55 (s, 1H, N*H*), 8.13–7.88 (m, 2H, Ar*H*), 7.87–7.68 (m, 4H, Ar*H*), 7.67–7.60 (m, 1H, Ar*H*), 7.58 (s, 1H, Ar*H*), 7.31–7.02 (m, 3H, Ar*H*), 7- 6.68 (m, 2H, Ar*H*), 5.69 (s, 2H, CO-C*H*_*2*_*-*O); ^13^C NMR (100 MHz, DMSO-*d*_6_) δ 193.59, 168.90, 159.17, 142.70, 140.29, 135.89, 133.30, 131.93, 129.94, 127.98, 127.05, 125.76, 124.27, 122.09, 121.04, 119.30, 114.98, 110.07, 70.23; Anal. Calcd. for C_23_H_16_BrNO_3_ (433.03): C, 63.61; H, 3.71; N, 3.23, Found: C, 63.62; H, 3.72; N, 3.25; ESI/MS: m/z Calcd. For [M-H]^−^: 432.03, Found: 431.4.

##### 6-Chloro-3-{4-[2-(4-bromophenyl)-2-oxoethoxy]benzylidene}indolin-2-one (6g)

Dark orange powder; 0.16g, 35.31% yield; mp: 249–251 °C; *R*_f_ = 0.31 (Pet.ether/ethyl acetate, 6:2.5); ^1^H NMR (400 MHz, DMSO-*d*_6_) δ 10.73 (s, 1H, N*H*), 8.44 (d, *J* = 8.8 Hz, 2H, Ar*H*), 7.97 (d, *J* = 8.4 Hz, 2H, Ar*H*), 7.85–7.77 (m, 3H, Ar*H*), 7.70 (d, *J* = 8.1 Hz, 1H, Ar*H*), 7.09 (d, *J* = 8.8 Hz, 2H, Ar*H*), 7.05–7.00 (m, 1H, Ar*H*), 6.87–6.80 (m, 1H, Ar*H*), 5.69 (s, 2H, CO-C*H*_*2*_*-*O);); ^13^C NMR (100 MHz, DMSO-*d*_6_) δ 193.98, 169.30, 159.80, 144.47, 137.27, 134.91, 134.06, 132.38, 132.03, 130.40, 128.44, 127.24, 125.03, 123.75, 121.25, 120.54, 115.54, 110.45, 70.70; Anal. Calcd. for C_23_H_15_BrClNO_3_ (466.99): C, 58.94; H, 3.23; N, 2.99, Found: C, 58.96; H, 3.24; N, 3.00; ESI/MS: m/z Calcd. For [M-H]^−^: 465.99, Found: 465.6.

##### 3-{4-[2-(4-Methoxyphenyl)-2-oxoethoxy]benzylidene}indolin-2-one (6h)

Yellow powder; 0.22 g, 59.37% yield; mp: 192–194 °C;; *R*_f_ = 0.37 (Pet.ether/ethyl acetate, 6:2.5); ^1^H NMR (400 MHz, DMSO-*d*_6_) δ 10.74 (s, 1H, N*H*), 8.05 (d, *J* = 8.6 Hz, 2H, Ar*H*), 7.72 (d, *J* = 8.6 Hz, 2H, Ar*H*), 7.67 (s, 1H, Ar*H*), 7.65 (d, *J* = 3.4 Hz, 1H, Ar*H*), 7.13 (d, *J* = 8.8 Hz, 4H, Ar*H*), 7.10–7.03 (m, 1H, Ar*H*), 6.96 (dd, *J* = 8.3, 2.0 Hz, 1H, Ar*H*), 6.91 (d, *J* = 2.0 Hz, 1H, Ar*H*), 5.66 (s, 2H, CO-C*H*_*2*_*-*O), 3.90 (s, 3H, OC*H*_*3*_); ^13^C NMR (100 MHz, DMSO-*d*_6_) δ 192.41, 168.86, 163.66, 159.57, 143.99, 136.86, 134.45, 131.58, 130.29, 127.18, 124.32, 123.31, 120.80, 120.09, 119.82, 115.05, 114.10, 109.98, 69.97, 55.65; Anal. Calcd. for C_24_H_19_NO_4_ (385.13): C, 74.79; H, 4.97; N, 3.63, Found: C, 74.80; H, 4.99; N, 3.64; ESI/MS: m/z Calcd. For [M + Cl] ^−^: 420.03, Found: 419.2.

##### 5-Chloro-3-{4-[2-(4-methoxyphenyl)-2-oxoethoxy]benzylidene}indolin-2-one (6i)

Yellow powder; 0.22 g, 51.31% yield; mp: 183–185 °C; *R*_f_ = 0.29 (Pet.ether/ethyl acetate, 6:2.5); ^1^H NMR (400 MHz, DMSO-*d*_6_) δ 10.55 (s, 1H, N*H*), 8.03 (d, *J* = 8.9 Hz, 2H, Ar*H*), 7.74–7.63 (m, 2H, Ar*H*), 7.58 (s, 1H, Ar*H*), 7.22 (t, *J* = 7.8 Hz, 1H, Ar*H*), 7.11 (d, *J* = 8.9 Hz, 4H, Ar*H*), 6.92–6.83 (m, 2H, Ar*H*), 5.63 (s, 2H, CO-C*H*_*2*_*-*O), 3.87 (s, 3H, OC*H*_*3*_); ^13^C NMR (100 MHz, DMSO-*d*_6_) δ 192.96, 169.34, 164.09, 159.87, 143.11, 140.70, 136.36, 134.67, 130.72, 130.17, 127.63, 126.11, 124.61, 122.52, 121.57, 115.38, 114.53, 110.48, 70.39, 56.08; Anal. Calcd. for C_24_H_18_ClNO_4_ (419.09): C, 68.66; H, 4.32; N, 3.34, Found: C, 68.67; H, 4.33; N, 3.36; ESI/MS: m/z Calcd. For [M-H] ^−^: 418.08, Found: 417.5.

##### (*E*/*Z*)-6-Chloro-3-{4-[2-(4-methoxyphenyl)-2-oxoethoxy]benzylidene}indolin-2-one (6j). *E*: *Z* ratio = 38.5: 61.5

Yellow powder; 0.22 g, 51.31% yield; mp: 165–167 °C; *R*_f_ = 0.27 (Pet.ether/ethyl acetate, 6:2.5);

*Z*-Diastereomer: ^1^H NMR (400 MHz, DMSO-*d*_6_) δ 10.73 (s, 1H, N*H*), 8.49 (d, *J* = 9 Hz, 1H, Ar*H*), 8.06 (d, *J* = 2.9 Hz, 1H, Ar*H*), 8.04 (d, *J* = 2.9 Hz, 1H, Ar*H*), 7.92 (s, 1H, Ar*H*), 7.83 (dd, *J* = 9.7, 2.2 Hz, 1H, Ar*H*), 7.72 (d, *J* = 8.7 Hz, 1H, Ar*H*), 7.37–7.15 (m, 4H, Ar*H*), 6.91 (d, *J* = 8.4 Hz, 1H, Ar*H*), 6.84 (d, *J* = 8.4 Hz, 1H, Ar*H*), 5.68 (s, 2H, CO-C*H*_*2*_*-*O), 3.90 (s, 3H, OC*H*_*3*_) ppm; ^13^C NMR (100 MHz, DMSO-*d*_6_) δ 192.36, 167.13, 161.61, 159.70, 140.97, 138.74, 137.87, 133.30, 130.27, 129.19, 127.30, 127.19, 124.87, 122.80, 121.45, 114.51, 114.08, 110.52, 69.91, 55.60 ppm.

*E*-Diastereomer: ^1^H NMR (400 MHz, DMSO-*d*_6_) δ 10.69 (s, 1H, N*H*), 8.44 (d, *J* = 9 Hz, 1H, Ar*H*), 8.00 (d, *J* = 2.9 Hz, 1H, Ar*H*), 7.97 (d, *J* = 2.9 Hz, 1H, Ar*H*), 7.69 (s, 1H, Ar*H*), 7.63 (dd, *J* = 9.7, 2.2 Hz, 1H, Ar*H*), 7.59 (d, *J* = 8.7 Hz, 1H, Ar*H*), 7.14–7.07 (m, 4H, Ar*H*), 6.76 (d, *J* = 8.4 Hz, 1H, Ar*H*), 6.70 (d, *J* = 8.4 Hz, 1H, Ar*H*), 5.66 (s, 2H, CO-C*H*_*2*_*-*O), 3.86 (s, 3H, OC*H*_*3*_) ppm; ^13^C NMR (100 MHz, DMSO-*d*_6_) δ 192.43, 168.56, 163.63, 160.37, 141.42, 138.86, 138.02, 134.62, 131.51, 129.61, 127.6, 127.19, 125.27, 123.02, 121.65, 115.05, 114.08, 111.38, 69.97, 55.63 ppm.

Anal. Calcd. for C_24_H_18_ClNO_4_ (419.09): C, 68.66; H, 4.32; N, 3.34, Found: C, 68.69; H, 4.33; N, 3.35; ESI/MS: m/z Calcd. For [M + Na] ^+^: 442.09, Found: 441.8.

### Biological investigation

#### Cytotoxic assays

##### One-dose assay of compounds 6a–j against sixty NCI cancer cell lines

National Cancer Institution selected all the synthesized **6a–j** (NCI), Bethesda, USA, for in vitro* one*-dose antitumor assay. The methodology protocol for the NCI anticancer screening was reported in detail elsewhere (http://www.dtp.nci.nih.gov). The anticancer assay was performed in full NCI 60 cell lines extracted from nine tumor cell lines, including leukemia, melanoma, lung, colon, CNS, ovarian, renal, prostate, and breast cancer cell lines.

##### *Cytotoxicity versus MCF-7 cell line* [70]

Cells were preserved at Roswell Park Memorial Institute media (RPMI media). The test was achieved using 0.001–100 μM of compounds **6f** and **6g**. IC_50_ values were recorded using the Boltzman sigmoidal concentration–response curve equation using the nonlinear regression fitting models by GraphPad, Prism version 5 (GraphPad Software Inc., La Jolla, CA). MTT assay for MCF-7 cell lines. Cell line cells were obtained from American Type Culture Collection. Cells were cultured using DMEM media (Invitrogen/Life Technologies) supplemented with 10% FBS (Hyclone), 10 ug/ml of insulin (Sigma), and 1% penicillin–streptomycin. All the other chemicals and reagents were purchased from Sigma or Invitrogen. Cells were seeded in plates (cells density 1.2–1.8 × 10,000 cells/well) in a volume of 100 μl full-growth medium + 100 μl of the tested scaffold per well in a 96-well plate for 24 h before the application of the MTT assay protocol as reported, supplementary information.

##### Margin of safety assessment upon detection of cytotoxicity on human skin fibroblasts (HSF) cells

Cell viability was assessed via SRB assay. Aliquots of 100 µl cell suspension (5 × 10^3^ cells) were in 96-well plates and incubated in a complete media for 24 h. Cells were treated with another aliquot of 100 µl media containing compound **6f** at various concentrations (up to IC_50_ value against MCF-7 is equal to 19.43 µM). After 72 h of drug exposure, cells were fixed by replacing media with 150 µl of 10% TCA and incubated at 4 °C for 1 h. The TCA solution was removed, and the cells were washed 5 times with distilled water. Aliquots of 7 µl SRB solution (0.4 w/v) were added and incubated in a dark place at room temperature for 10 min. Plates were washed 3 times with 1% acetic acid and allowed to air-dry overnight. Then, 150 µl of TRIS (10 mM) was added to dissolve the protein-bound SRB stain; the absorbance was measured at 540 nm using a BMG LAB TECH®-FLUO star Omega microplate reader (Ortenberg, Germany) [85, 86].

#### Cell cycle arrest analysis

MCF-7 Cells were placed in a six-well plate at 1 × 10^5^ concentration of cells/well and then kept for incubation for 24 h. MCF-7 Cells were treated with compound **6f** (19.43 μM) for 24 h. Cells were then collected and fixed for 12 h using ice-cold 70% ethanol at 4 °C. Ethanol was then removed, and cells were washed with cold phosphate buffer saline (PBS, 0.5 ml) and preserved for 30 min at 37 °C. The cells were stained for 30 min with propidium iodide in the dark. A flow cytometer was used to detect DNA content [87].

#### RTK kinases’ circumvention

The receptor tyrosine kinases, namely EGFR, VEGFR-2, and PDGFR_ẞ Kinases Assay Kits, are designed to measure EGFR, VEGFR-2, and PDGFR_ẞ Kinases’ activities for screening and profiling applications using Kinase-Glo® MAX as a detection reagent [70]. The EGFR, VEGFR-2, and PDGFR_ẞ Kinases Assay Kits come in a convenient 96-well format, with enough purified recombinant EGFR enzyme, EGFR substrate, ATP, and kinase assay buffer for 100 enzyme reactions. The master mixture (25 µl per well) was equipped out of Kinase assay buffer (6 µl), ATP (500 µM, 1 µl), PTK substrate (50x, 1 µl), and water (17 µl) and transferred into each well. The inhibitor solution was made by dissolving compound **6f** in a “liquid labeled” “inhibitor buffer.” An inhibitor solution (5 µl) was added to each well, labeled “Test Inhibitor.” For “Positive Control” and “Blank,” add 5 µl inhibitor buffer). EGFR, VEGFR-2, and PDGFR_ẞ Kinases were softened on ice. Its tube was spun briefly to recover its full content of the tube. These enzymes were diluted with 1 × Kinase assay buffer to 1 ng/ µl. The reaction was initiated by adding 20 µl of diluted EGFR, VEGFR-2, and PDGFR_ẞ enzymes to the wells designated “Positive Control” and “Test Inhibitor Control.” The plate was incubated at 30 °C for 40 min. After the 40-min reaction, the Kinase-Glo Max (50 µl) reagent was added to each well. The plate was covered with aluminum foil and incubated at room temperature for 15 min, and the luminescence was measured using the microplate reader.

#### Tubulin polymerization inhibitory activity

The ability of compound **6f** to circumvent tubulin polymerization was distinguished using a fluorescent kit (cytoskeleton, Catalog number BK011P) and Tecan-spark reader according to the prescribed manufacturer protocol (Danvers, MA, USA) [88]. Solutions of compound **6f** in DMSO (10%, 5 µl) were added (in triplicate) to the wells of a 96-well plate. Then, 45 µl of each reaction mixture containing 2 mg/ml tubulin (> 99% pure), PEM buffer (pH 6.9), piperazine-N,N'-bis(2-ethane sulfonic acid) sequisodium salt (80 mM), EGTA (0.5 mM), MgCl_2_ (2 mM), GTP (1 mM), glycerol (10.2%), and 10 µM of fluorescent reporter, DAPI (4′,6-Diamidino-2 phenylindole) was warmed for 1 min at 37 °C before the addition of tubulin. Tubulin polymerization was detected by measuring the fluorescence emission at ƛ = 450 nm (excitation ƛ = 360 nm) for 60 min intervals. The resulting data IC_50_ for each compound were calculated using GraphPad Prism software.

### Docking studies

The new designed compounds (i.e., **6a–j**) were drawn and docked in the active site of VEGFR-2, EGFR, and tubulin enzymes (**PDB IDs: 1YWN**, **4HJO**, and **5LYJ**, respectively) using Auto Dock Vina software.4.2 program as reported in the literature.

### Supplementary Information

Below is the link to the electronic supplementary material.

Supplementary file1 (DOCX 7425 KB)
