# Supplementary material for: New antiproliferative 3-substituted oxindoles inhibiting EGFR/VEGFR-2 and tubulin polymerization
Source: Mol Divers. 2023 Feb 15;28(2):563–80. doi: 10.1007/s11030-023-10603-z (PMC11070402; doi:10.1007/s11030-023-10603-z)
Supplement: Supplementary file 1 — Supplementary file1 (DOCX 7425 KB) [file 11030_2023_10603_MOESM1_ESM.docx]

**Supporting information**

**New Antiproliferative 3-Substituted Oxindoles Inhibiting** **EGFR/VEGFR-2 and Tubulin Polymerization**

**Hend A. A.** [**Ezelarab**](https://www.researchgate.net/profile/Hend_Ezelarab?_sg%5B0%5D=mkPHDtm-YANKygOyzayqFrjB3TeL8vEHhPoI5d0hRegJDi18-96m7mB75kJExYHROIl0Qk0.FFpfqe6owJHXeo6u7rcAFPhhI2_xdllgqU8WgE-d_34UjHOBsc1Wu3WgrY2Gksn08gkHDUzt7bPn4TpO2PPV2Q&_sg%5B1%5D=VhRrT7PATBq1bQisAs0zLLUBQrQxkYSNJvp_QHkFnmtDTVACy61srBT9c5r0Sdp7PSICZR4pb4CQHWPs.UOmMD95eiSa04RnTM70npebwuV9Ml-hMcWezDC2uJLxfT47d2vVC_XmKF2RG4vNAG5Sd1IAg81JzwzxV46SM-g)**^1^, Taha F. S. Ali*^1^,** [**Samar H. Abbas**](https://www.researchgate.net/profile/Samar_Abbas6?_sg%5B0%5D=mkPHDtm-YANKygOyzayqFrjB3TeL8vEHhPoI5d0hRegJDi18-96m7mB75kJExYHROIl0Qk0.FFpfqe6owJHXeo6u7rcAFPhhI2_xdllgqU8WgE-d_34UjHOBsc1Wu3WgrY2Gksn08gkHDUzt7bPn4TpO2PPV2Q&_sg%5B1%5D=VhRrT7PATBq1bQisAs0zLLUBQrQxkYSNJvp_QHkFnmtDTVACy61srBT9c5r0Sdp7PSICZR4pb4CQHWPs.UOmMD95eiSa04RnTM70npebwuV9Ml-hMcWezDC2uJLxfT47d2vVC_XmKF2RG4vNAG5Sd1IAg81JzwzxV46SM-g)***^1^, Ahmed M. Sayed^2^,** **Eman A. M. Beshr*^1^,** [**Heba A. Hassan**](https://www.researchgate.net/profile/Heba_Hassan30?_sg%5B0%5D=mkPHDtm-YANKygOyzayqFrjB3TeL8vEHhPoI5d0hRegJDi18-96m7mB75kJExYHROIl0Qk0.FFpfqe6owJHXeo6u7rcAFPhhI2_xdllgqU8WgE-d_34UjHOBsc1Wu3WgrY2Gksn08gkHDUzt7bPn4TpO2PPV2Q&_sg%5B1%5D=VhRrT7PATBq1bQisAs0zLLUBQrQxkYSNJvp_QHkFnmtDTVACy61srBT9c5r0Sdp7PSICZR4pb4CQHWPs.UOmMD95eiSa04RnTM70npebwuV9Ml-hMcWezDC2uJLxfT47d2vVC_XmKF2RG4vNAG5Sd1IAg81JzwzxV46SM-g)**^1^**

^1^ Department of Medicinal Chemistry, Faculty of Pharmacy, Minia University, 61519-Mini, Egypt.

^2^ Department of Pharmacognosy, Faculty of Pharmacy, Nahda University, 62513 Beni-Suef, Egypt.

***** Corresponding Author

Samar H. Abbas; Phone: (+20) 100-542-4005. Fax: (+20) 086-236-9075. E-mail: [samar_hafez@mu.edu.eg](mailto:samar_hafez@mu.edu.eg).

Eman A. M. Beshr; eman_beshr@mu.edu.eg

Taha F. S. Ali; [taha.ali@mu.edu.eg](mailto:taha.ali@mu.edu.eg)

| **List of Figures** | | |
| --- | --- | --- |
| **Fig. No** |  | **Page No.** |
| **S1** | **^1^H NMR of compound 6a (400 MHz, DMSO- *d*_6_)** | **6** |
| **S2** | **^13^C NMR of compound 6a (100 MHz, DMSO- *d*_6_)** | **7** |
| **S3** | **MS (ESI^+^) spectrum of compound 6a**. | **8** |
| **S4** | **^1^H NMR of compound 6b (400 MHz, DMSO- *d*_6_)** | **9** |
| **S5** | **^13^C NMR of compound 6b (100 MHz, DMSO- *d*_6_)** | **10** |
| **S6** | **MS (ESI^+^) spectrum of compound 6b.** | **11** |
| **S7** | **MS (ESI^-^) spectrum of compound 6b**. | **12** |
| **S8** | **^1^H NMR of compound 6c (400 MHz, DMSO- *d*_6_)** | **13** |
| **S9** | **^13^C NMR of compound 6c (100 MHz, DMSO- *d*_6_)** | **14** |
| **S10** | **MS (ESI^-^) spectrum of compound 6c**. | **15** |
| **S11** | **^1^H NMR of compound 6d (400 MHz, DMSO- *d*_6_)** | **16** |
| **S12** | **^13^C NMR of compound 6d (100 MHz, DMSO- *d*_6_) (*E* isomer)** | **17** |
| **S13** | **^13^C NMR of compound 6d (100 MHz, DMSO- *d*_6_) (*Z* isomer)** | **18** |
| **S14** | **MS (ESI^+^) spectrum of compound 6d.** | **19** |
| **S15** | **MS (ESI^-^) spectrum of compound 6d**. | **20** |
| **S16** | **^1^H NMR of compound 6e (400 MHz, DMSO- *d*_6_)** | **21** |
| **S17** | **^13^C NMR of compound 6e (100 MHz, DMSO- *d*_6_)** | **22** |
| **S18** | **MS (ESI^+^) spectrum of compound 6e.** | **23** |
| **S19** | **MS (ESI^-^) spectrum of compound 6e**. | **24** |
| **S20** | **^1^H NMR of compound 6f (400 MHz, DMSO- *d*_6_)** | **25** |
| **S21** | **^13^C NMR of compound 6f (100 MHz, DMSO- *d*_6_)** | **26** |
| **S22** | **MS (ESI^-^) spectrum of compound 6f.** | **27** |
| **S23** | **^1^H NMR of compound 6g (400 MHz, DMSO- *d*_6_)** | **28** |
| **S24** | **^13^C NMR of compound 6g (100 MHz, DMSO- *d*_6_)**. | **29** |
| **S25** | **MS (ESI^-^) spectrum of compound 6g**. | **30** |
| **S26** | **^1^H NMR of compound 6h (400 MHz, DMSO- *d*_6_)** | **31** |
| **S27** | **^13^C NMR of compound 6h (100 MHz, DMSO- *d*_6_)**. | **32** |
| **S28** | **MS (ESI^-^) spectrum of compound 6h**. | **33** |
| **S29** | **^1^H NMR of compound 6i (400 MHz, DMSO- *d*_6_)** | **34** |
| **S30** | **^13^C NMR of compound 6i (100 MHz, DMSO- *d*_6_)**. | **35** |
| **S31** | **MS (ESI^+^) spectrum of compound 6i.** | **36** |
| **S32** | **^1^H NMR of compound 6j (400 MHz, DMSO- *d*_6_)** | **37** |
| **S33** | **^13^C NMR of compound 6j (100 MHz, DMSO- *d*_6_) (*E* isomer)** | **38** |
| **S34** | **^13^C NMR of compound 6j (100 MHz, DMSO- *d*_6_) (*Z* isomer)** | **39** |
| **S35** | **MS (ESI^+^) spectrum of compound 6j.** | **40** |
| **S36** | **MS (ESI^-^) spectrum of compound 6j.** | **41** |
| **S37** | **Epidermal growth factor receptor (EGFR) inhibition assay for compound 6f.** | **42 ,43** |
| **S38** | **Vascular endothelial growth factor (VEGFR-2) inhibition assay for compound 6f.** | **44,45** |
| **S39** | **Platelet-derived growth factor-ẞ (PDGF-ẞ) inhibition assay for compound 6f.** | **46,47** |
| **S40** | **Tubulin polymerization assay for compound 6f .** | **48,49** |
| **S41** | **IC_50_ of compounds 6f ,6g, and 5-FU against MCF-7 using SRB assay.** | **50** |
| **S42** | **IC_50_ of compounds 6f against HSF cells using SRB assay.** | **51** |
| **S43** | **IC_50_ of compounds 5-FU against HSF cells using SRB assay.** | **52** |
| **S44** | **One- dose NCI growth (%) and mean graph of compound 6a.** | **53** |
| **S45** | **One- dose NCI growth (%) and mean graph of compound 6b.** | **54** |
| **S46** | **One- dose NCI growth (%) and mean graph of compound 6c.** | **55** |
| **S47** | **One- dose NCI growth (%) and mean graph of compound 6d.** | **56** |
| **S48** | **One- dose NCI growth (%) and mean graph of compound 6e.** | **57** |
| **S49** | **One- dose NCI growth (%) and mean graph of compound 6f.** | **58** |
| **S50** | **One- dose NCI growth (%) and mean graph of compound 6g.** | **59** |
| **S51** | **One- dose NCI growth (%) and mean graph of compound 6h.** | **60** |
| **S52** | **One- dose NCI growth (%) and mean graph of compound 6i.** | **61** |
| **S53** | **One- dose NCI growth (%) and mean graph of compound 6j.** | **62** |
| **S54** | **2D diagram model showing compound 6a interactions into the VEGFR-2 kinase binding site (PDB code: 1YWN).** | **63** |
| **S55** | **2D diagram model showing compound 6b interactions into the VEGFR-2 kinase binding site (PDB code: 1YWN).** | **64** |
| **S56** | **2D diagram model showing compound 6c interactions into the VEGFR-2 kinase binding site (PDB code: 1YWN).** | **65** |
| **S57** | **2D diagram model showing compound 6d interactions into the VEGFR-2 kinase binding site (PDB code: 1YWN).** | **66** |
| **S58** | **2D diagram model showing compound 6e interactions into the VEGFR-2 kinase binding site (PDB code: 1YWN).** | **67** |
| **S59** | **2D diagram model showing compound 6f interactions into the VEGFR-2 kinase binding site (PDB code: 1YWN).** | **68** |
| **S60** | **2D diagram model showing compound 6g interactions into the VEGFR-2 kinase binding site (PDB code: 1YWN).** | **69** |
| **S61** | **2D diagram model showing compound 6h interactions into the VEGFR-2 kinase binding site (PDB code: 1YWN).** | **70** |
| **S62** | **2D diagram model showing compound 6i interactions into the VEGFR-2 kinase binding site (PDB code: 1YWN).** | **71** |
| **S63** | **2D diagram model showing compound 6j interactions into the VEGFR-2 kinase binding site (PDB code: 1YWN).** | **72** |
| **S64** | **2D diagram model showing erlotinib interactions into the VEGFR-2 kinase binding site (PDB code: 1YWN).** | **73** |
| **S65** | **2D diagram model showing sunitinib interactions into the VEGFR-2 kinase binding site (PDB code: 1YWN).** | **74** |
| **S66** | **2D diagram model showing compound 6a interactions into the EGFR kinase binding site (PDB code: 4HJO).** | **75** |
| **S67** | **2D diagram model showing compound 6b interactions into the EGFR kinase binding site (PDB code: 1YWN).** | **76** |
| **S68** | **2D diagram model showing compound 6c interactions into the EGFR kinase binding site (PDB code: 4HJO).** | **77** |
| **S69** | **2D diagram model showing compound 6d interactions into the EGFR kinase binding site (PDB code: 4HJO).** | **78** |
| **S70** | **2D diagram model showing compound 6e interactions into the EGFR kinase binding site (PDB code: 4HJO).** | **79** |
| **S71** | **2D diagram model showing compound 6f interactions into the EGFR kinase binding site (PDB code: 4HJO).** | **80** |
| **S72** | **2D diagram model showing compound 6g interactions into the EGFR kinase binding site (PDB code: 4HJO).** | **81** |
| **S73** | **2D diagram model showing compound 6h interactions into the EGFR kinase binding site (PDB code: 4HJO).** | **82** |
| **S74** | **2D diagram model showing compound 6i interactions into the EGFR kinase binding site (PDB code: 4HJO).** | **83** |
| **S75** | **2D diagram model showing compound 6j interactions into the EGFR kinase binding site (PDB code: 4HJO).** | **84** |
| **S76** | **2D diagram model showing erlotinib interactions into the EGFR kinase binding site (PDB code: 4HJO).** | **85** |
| **S77** | **2D diagram model showing sunitinib interactions into the EGFR kinase binding site (PDB code: 4HJO).** | **86** |
| **S78** | **2D diagram model showing compound 6a interactions into the CA-4 binding site of tubulin (PDB code: 5LYJ).** | **87** |
| **S79** | **2D diagram model showing compound 6b interactions into the CA-4 binding site of tubulin (PDB code: 5LYJ).** | **88** |
| **S80** | **2D diagram model showing compound 6c interactions into the CA-4 binding site of tubulin (PDB code: 5LYJ).** | **89** |
| **S81** | **2D diagram model showing compound 6d interactions into the CA-4 binding site of tubulin (PDB code: 5LYJ).** | **90** |
| **S82** | **2D diagram model showing compound 6e interactions into the CA-4 binding site of tubulin (PDB code: 5LYJ).** | **91** |
| **S83** | **2D diagram model showing compound 6f interactions into the CA-4 binding site of tubulin (PDB code: 5LYJ).** | **92** |
| **S84** | **2D diagram model showing compound 6g interactions into the CA-4 binding site of tubulin (PDB code: 5LYJ).** | **93** |
| **S85** | **2D diagram model showing compound 6h interactions into the CA-4 binding site of tubulin (PDB code: 5LYJ).** | **94** |
| **S86** | **2D diagram model showing compound 6i interactions into the CA-4 binding site of tubulin (PDB code: 5LYJ).** | **95** |
| **S87** | **2D diagram model showing compound 6j interactions into the CA-4 binding site of tubulin (PDB code: 5LYJ).** | **96** |
| **S88** | **2D diagram model showing combretastatin A-4 (CA-4) interactions into the CA-4 binding site of tubulin (PDB code: 5LYJ).** | **97** |

| **List of Tables** | | |
| --- | --- | --- |
| **Tab. No** |  | **Page No.** |
| **1** | **Growth inhibition % of compounds 6a-j versus 60 NCI cancer cell lines using 10 uM as a single dose assay.** | **98-99** |
| **2** | **Molecular modeling results for the synthesized compounds 6a-j, sunitinib, and erlotinib at the active binding site of VEGFR-2 protein kinase (PDB code: 1YWN).** | **100-101** |
| **3** | **Molecular modeling results for the synthesized compounds 6a-j, sunitinib, and erlotinib at the active binding site of EGFR protein kinase (PDB code: 4HJO).** | **102-103** |
| **4** | **Molecular modeling results for the synthesized compounds 6a-j, and CA-4 at the colchicine binding site of tubulin (PDB code: 5LYJ).** | **104-105** |


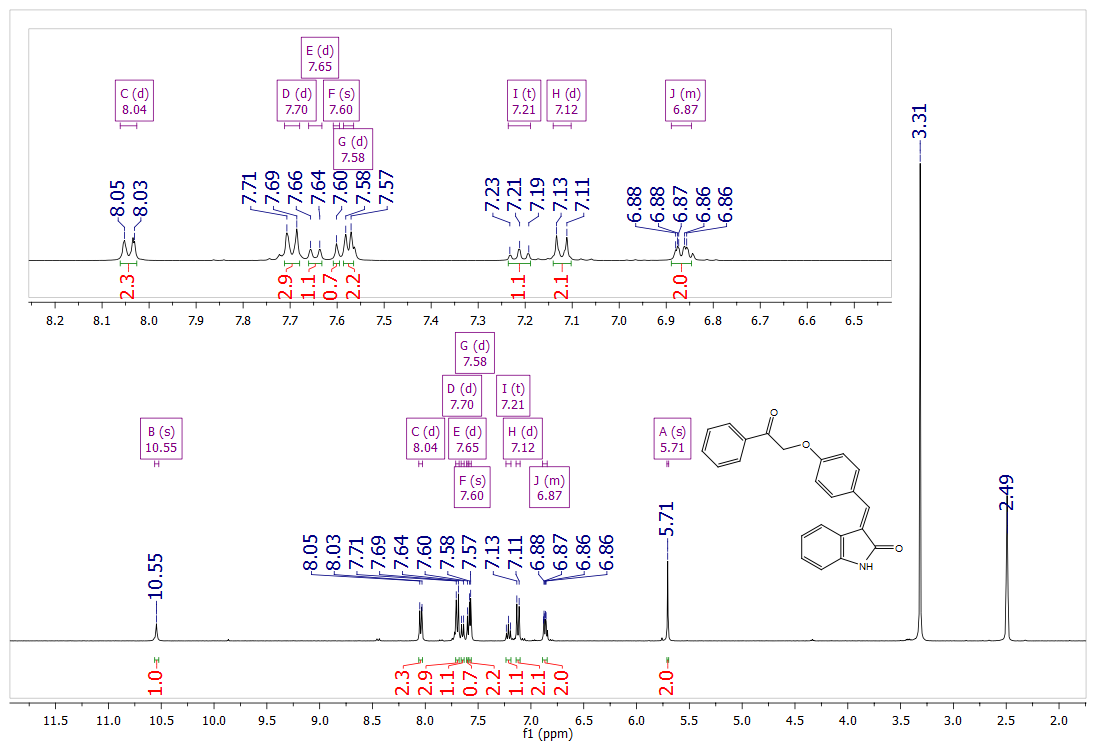


Figure S1. ^1^H NMR spectrum of compound **6a**.


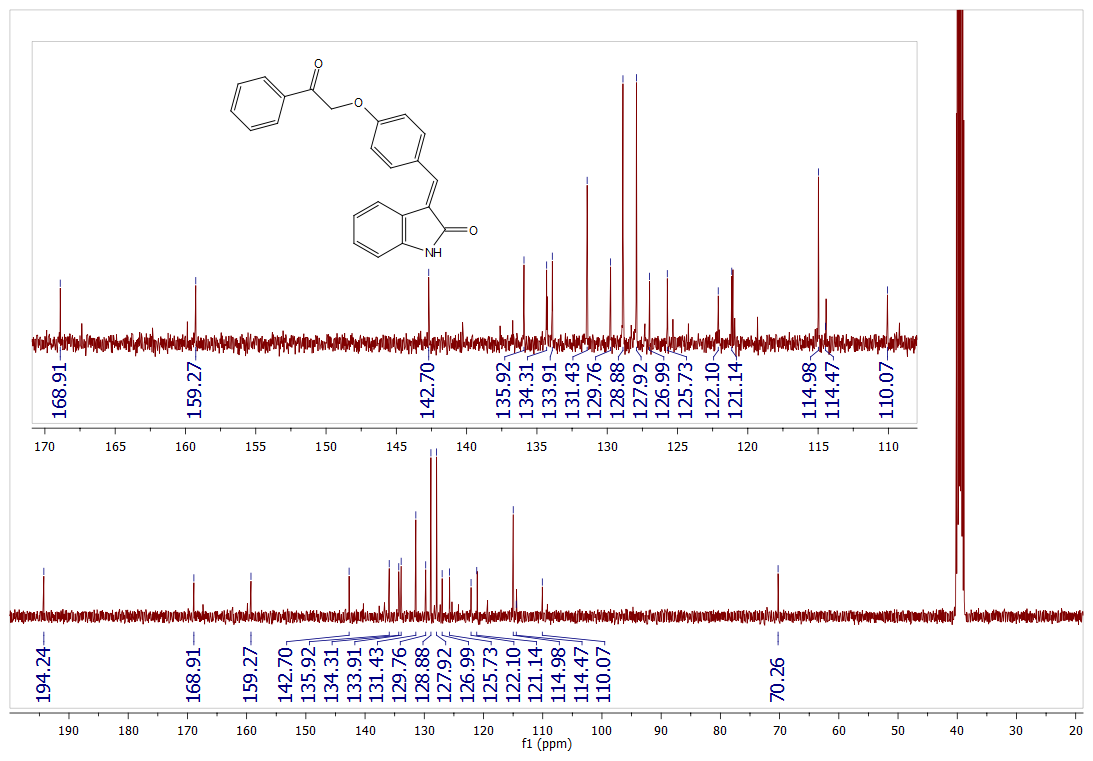


Figure S2. ^13^C NMR spectrum of compound **6a**.


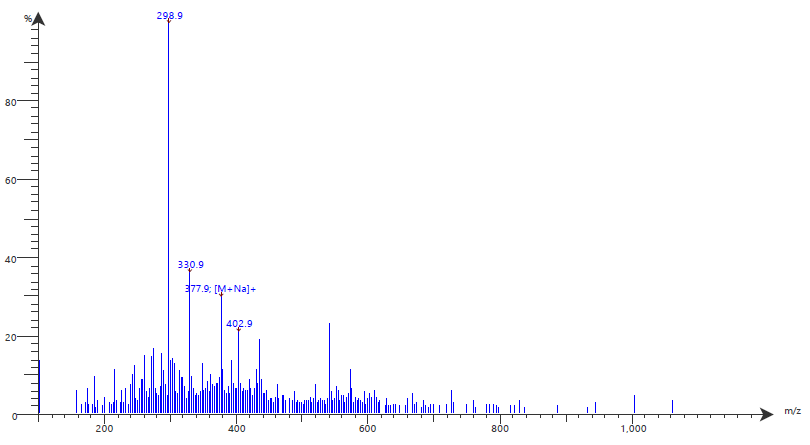


Figure S3. MS (ESI^+^) spectrum of compound **6a**.


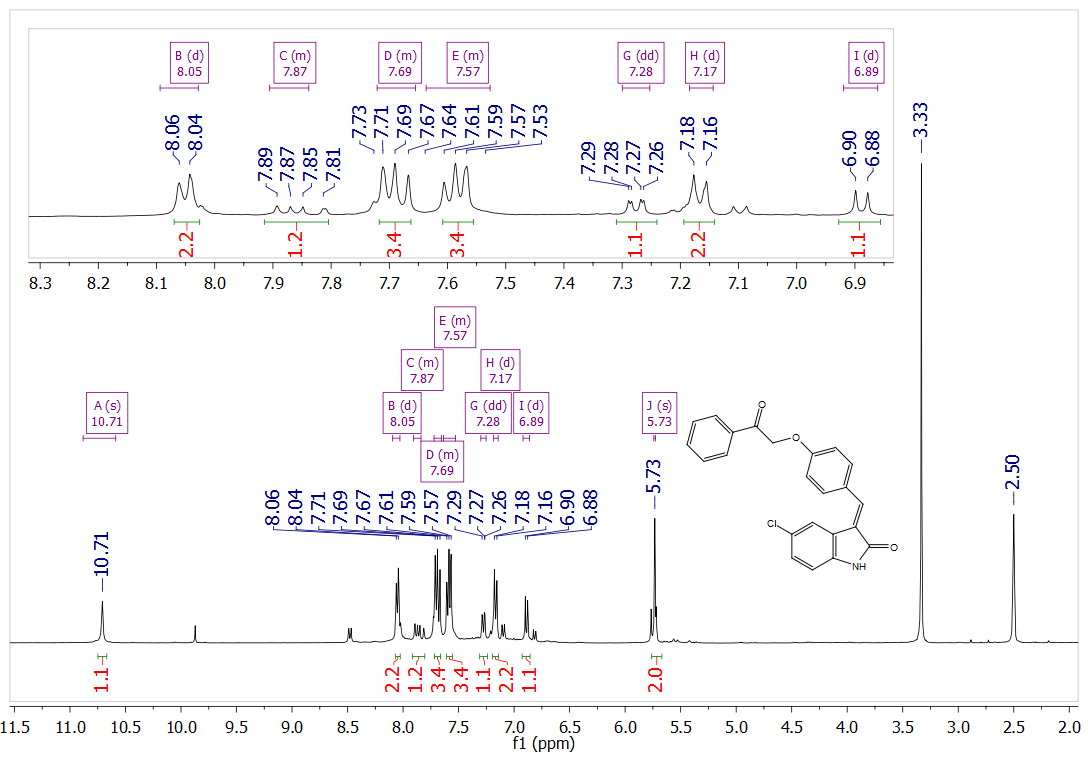


Figure S4. ^1^H NMR spectrum of compound **6b**.


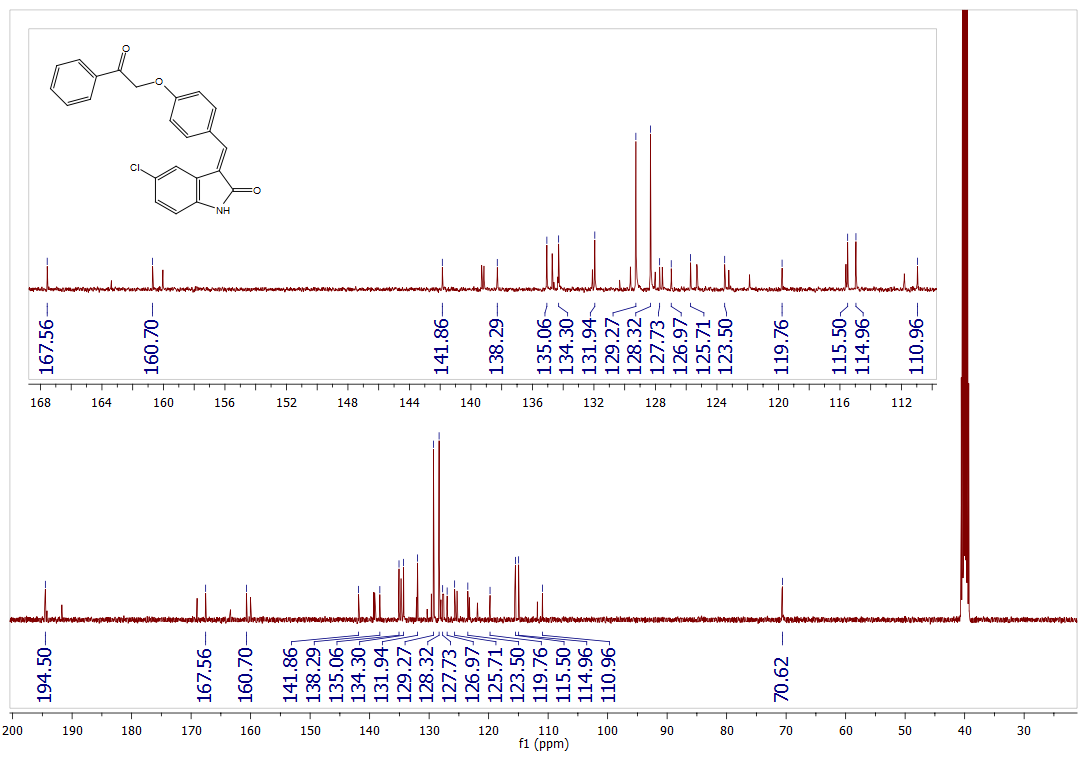


Figure S5. ^13^C NMR spectrum of compound **6b**.


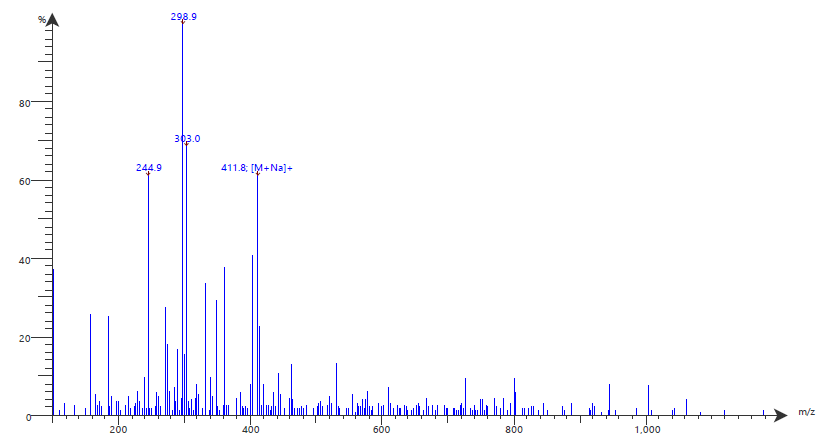

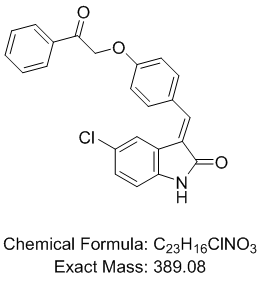


Figure S6. MS (ESI^+^) spectrum of compound **6b**.


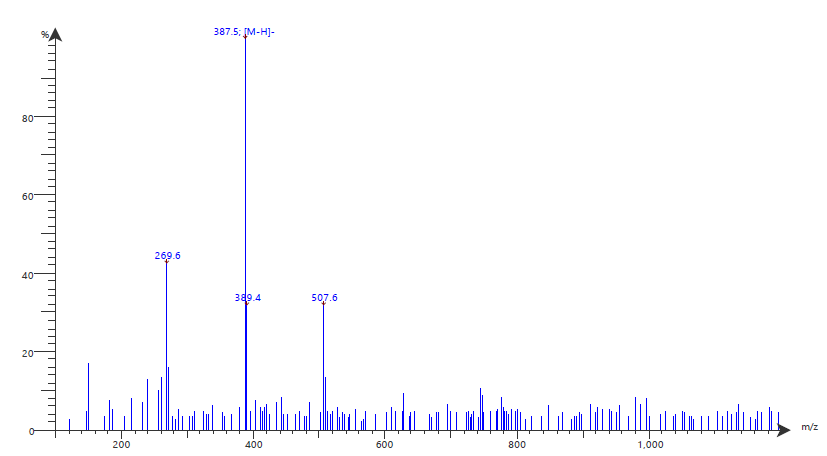

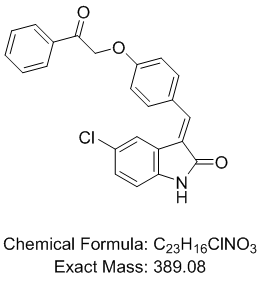


Figure S7. MS (ESI^-^) spectrum of compound **6b**.


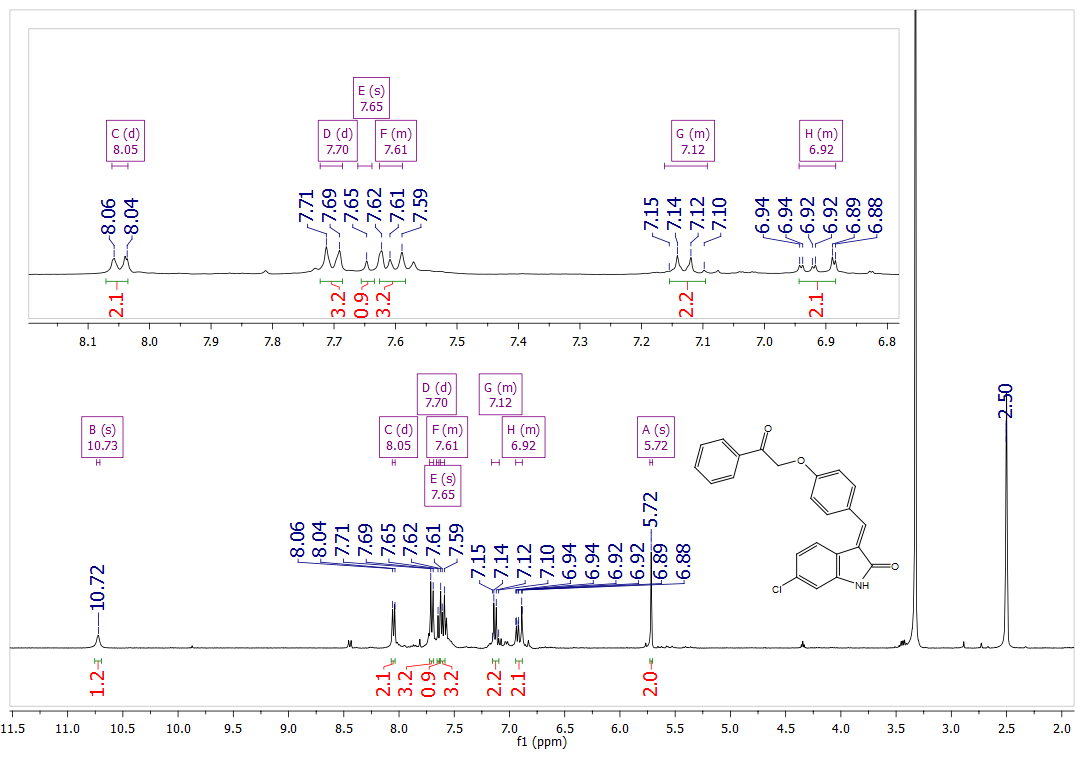


Figure S8. ^1^H NMR spectrum of compound **6c**.


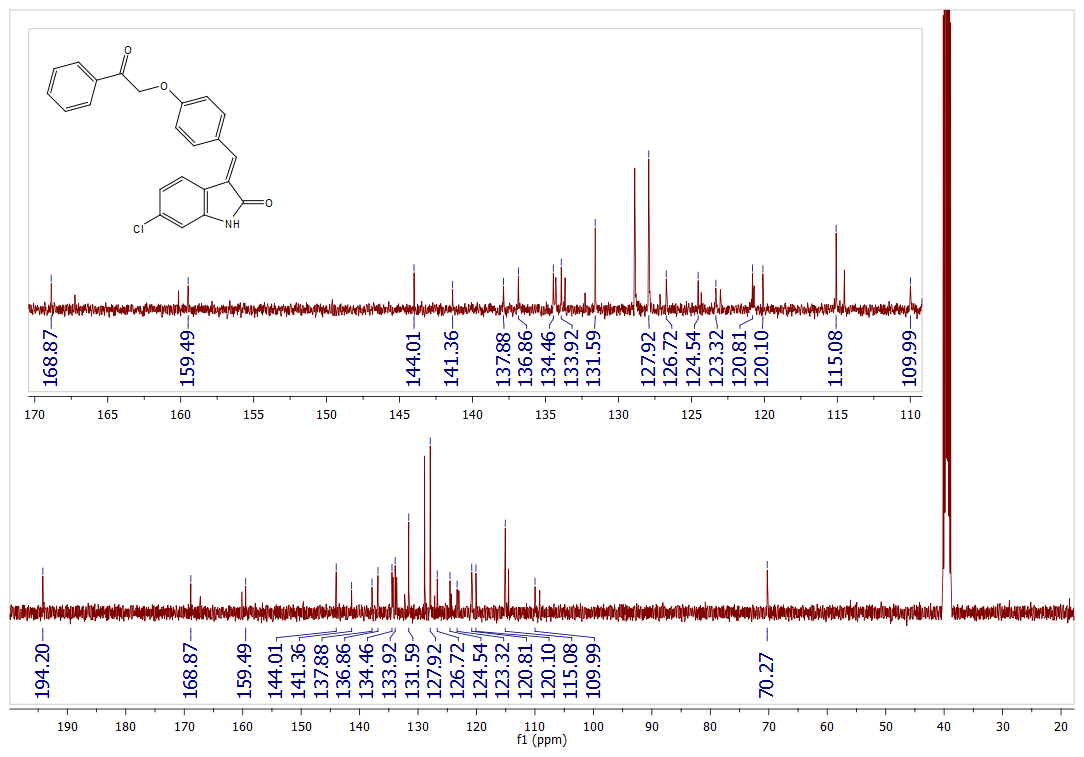


Figure S9. ^13^C NMR spectrum of compound **6c**.

Figure S10. MS (ESI^-^) spectrum of compound **6c**.


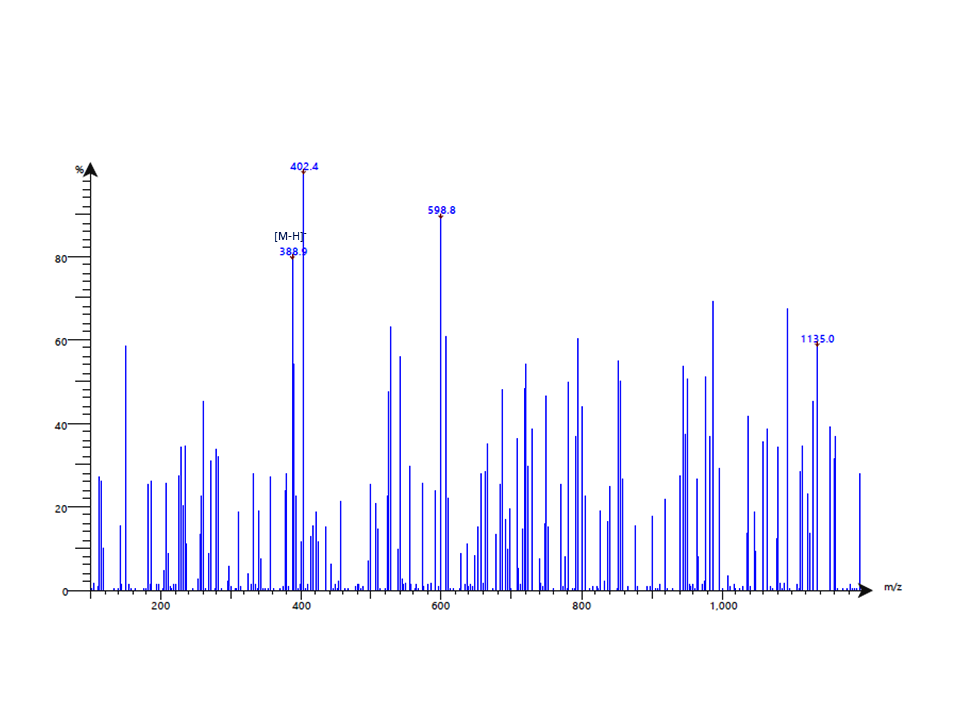


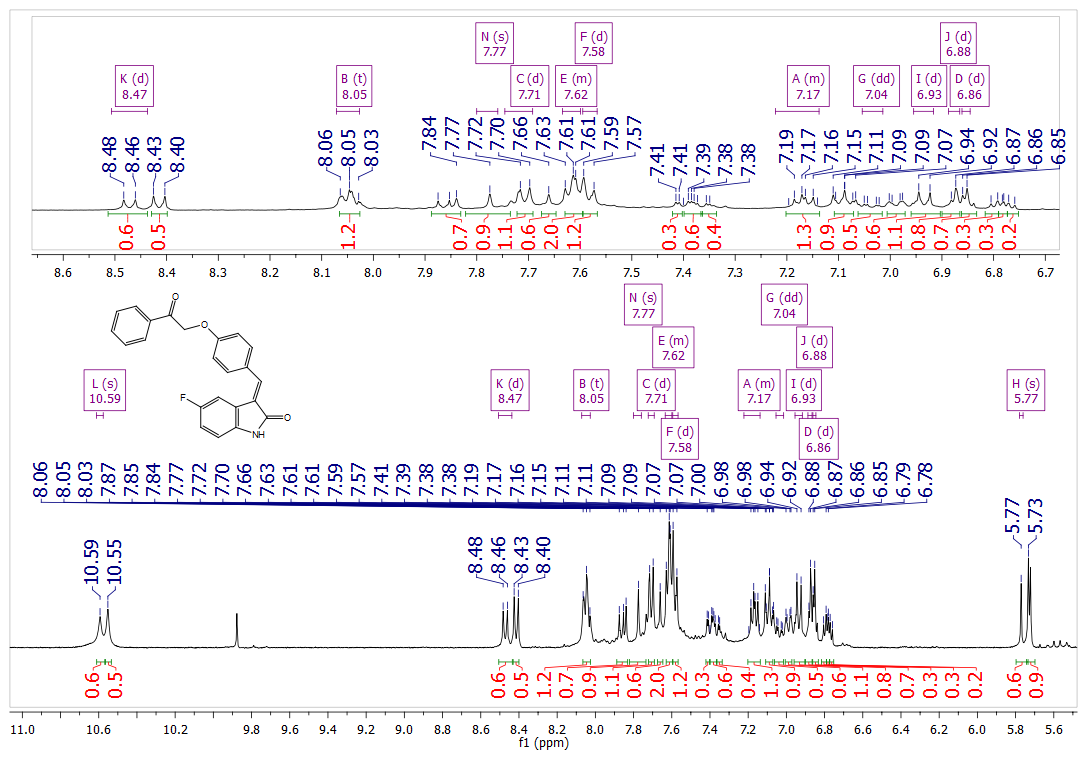


Figure S11. ^1^H NMR spectrum of compound **6d (*E/Z*)**.


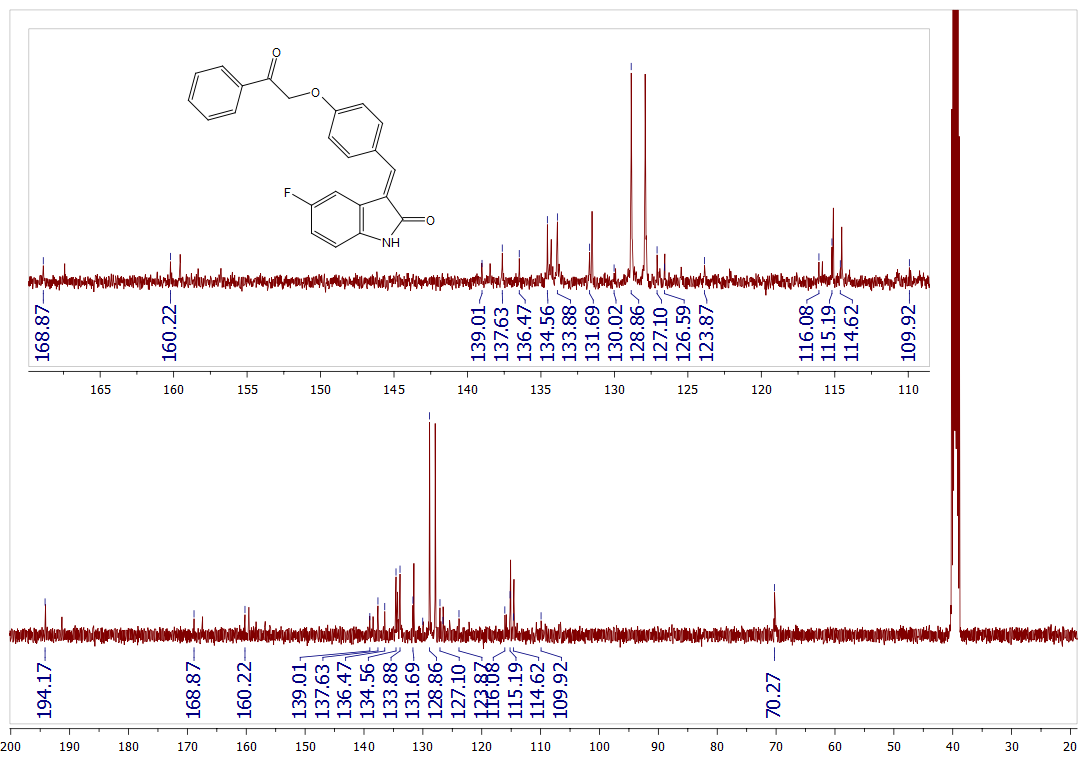


Figure S12. ^13^C NMR spectrum of compound **6d (*E* isomer)**.


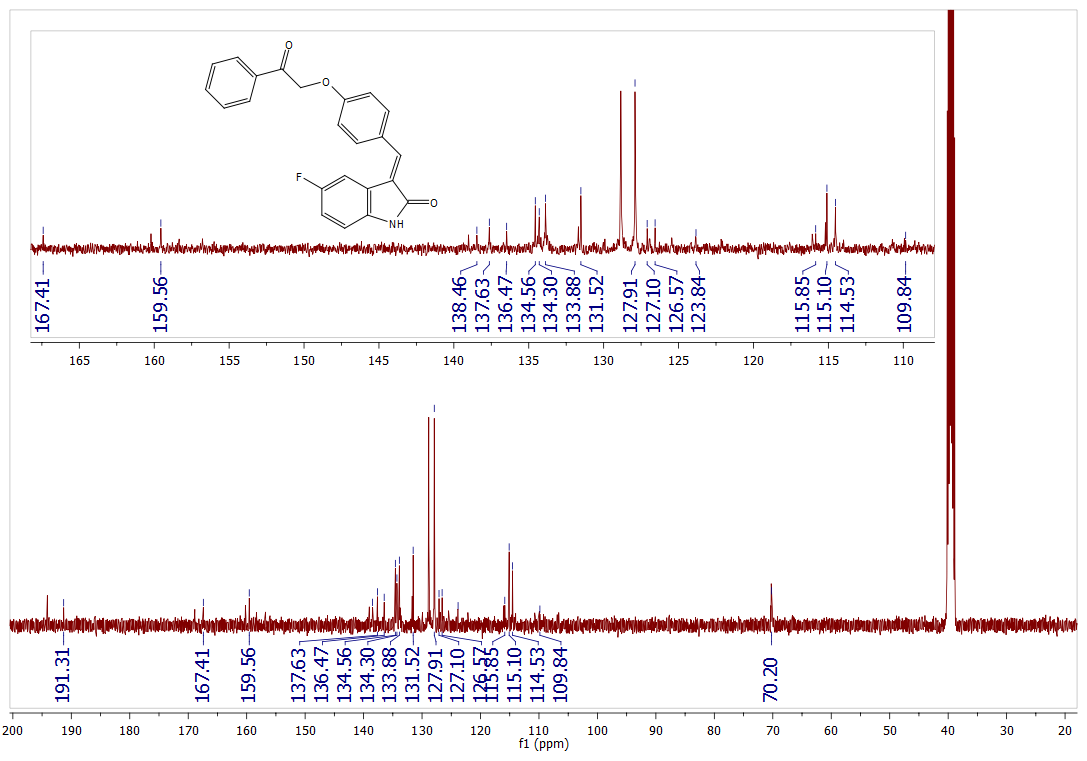


Figure S13. ^13^C NMR spectrum of compound **6d (*Z* isomer)**.

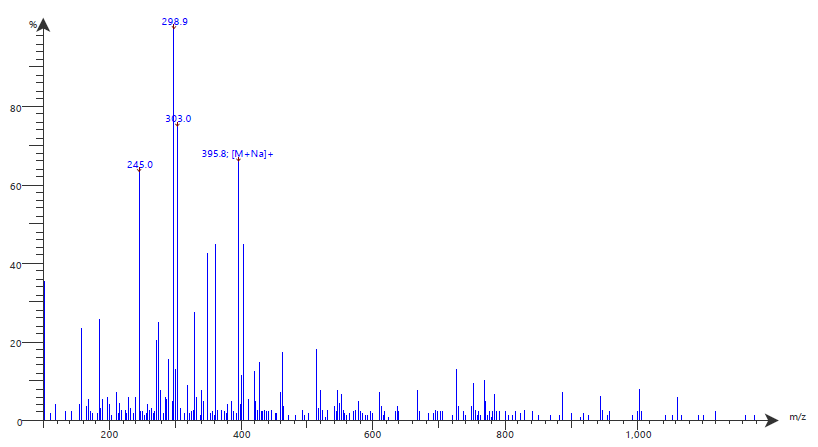

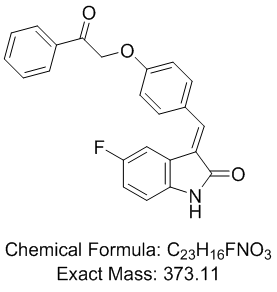


Figure S14. MS (ESI^+^) spectrum of compound **6d**.


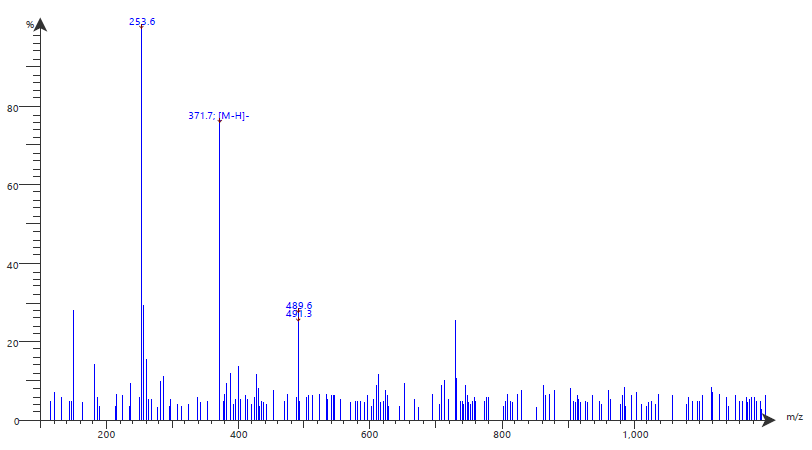


Figure S15. MS (ESI^-^) spectrum of compound **6d**.


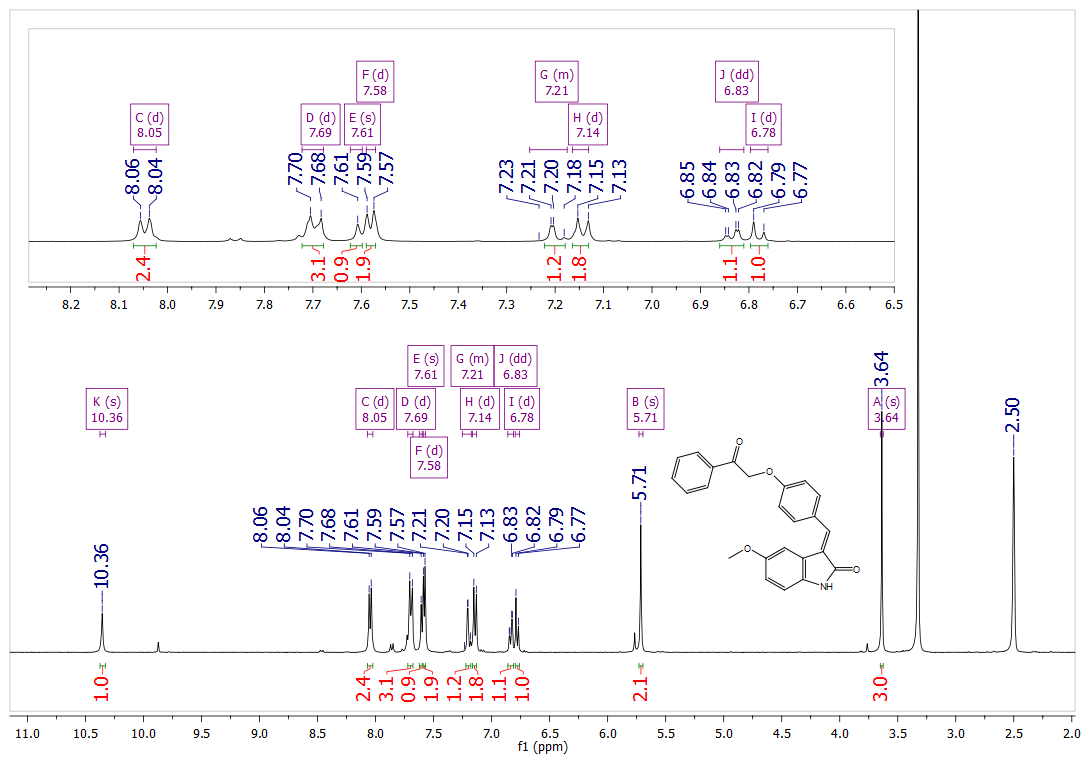


Figure S16. ^1^H NMR spectrum of compound **6e**.


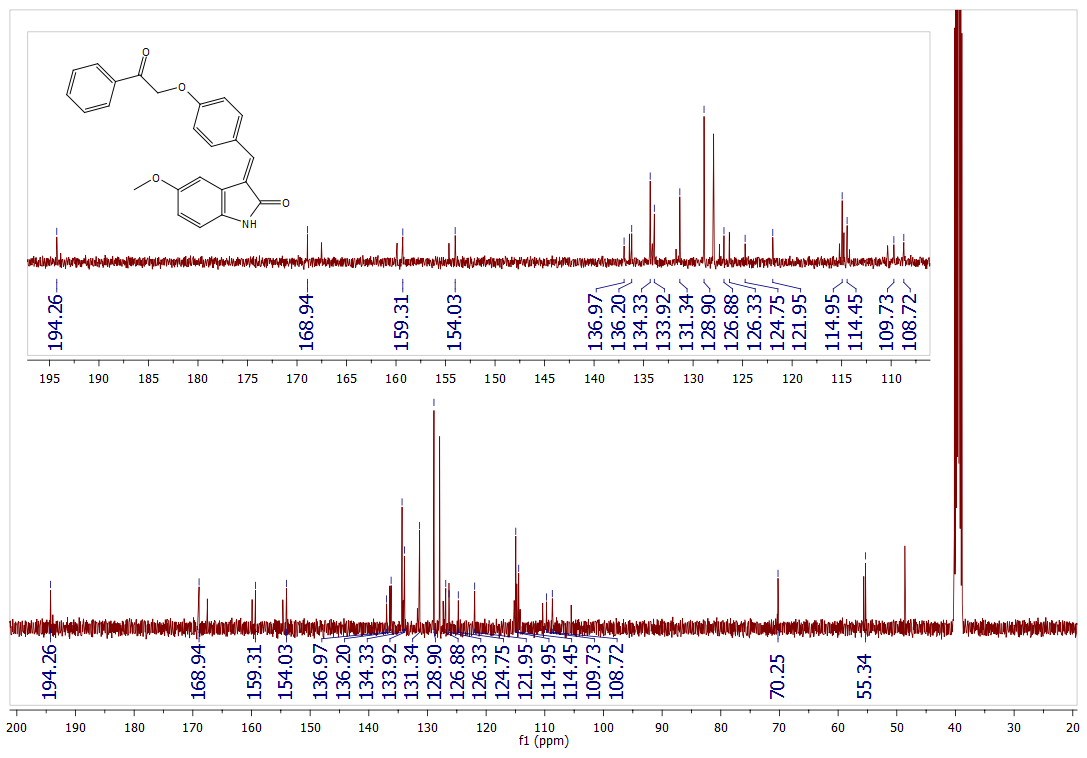


Figure S17. ^13^C NMR spectrum of compound **6e**.


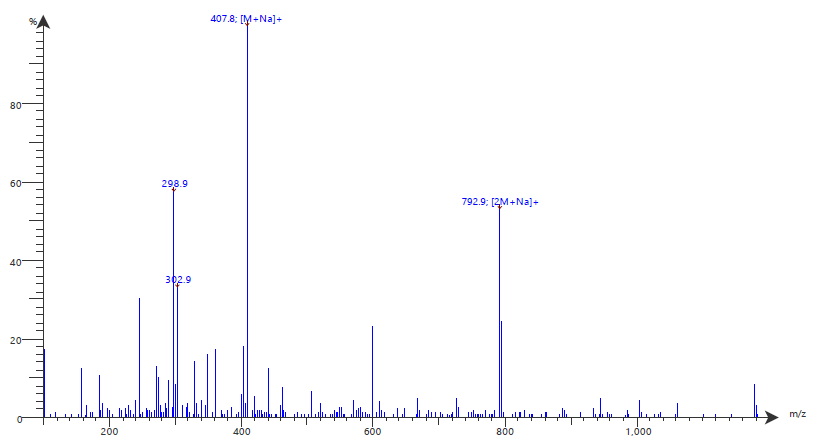

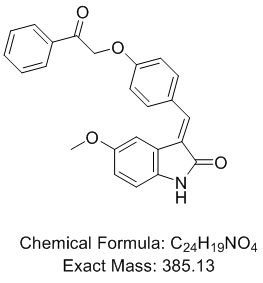


Figure S18. MS (ESI^+^) spectrum of compound **6e**.


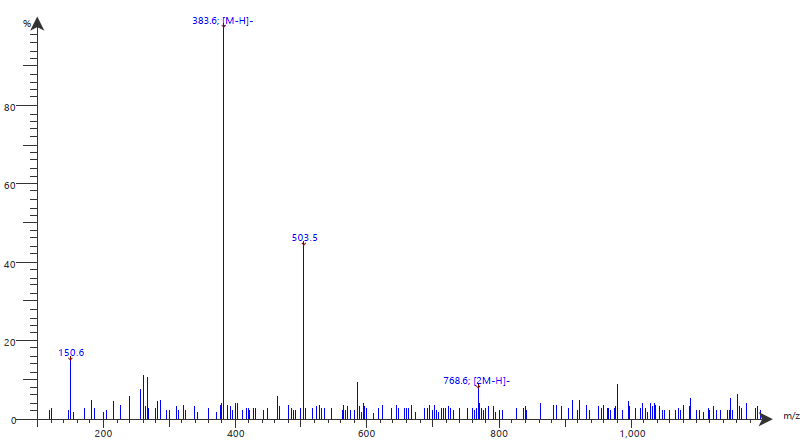


Figure S19. MS (ESI^-^) spectrum of compound **6e**.


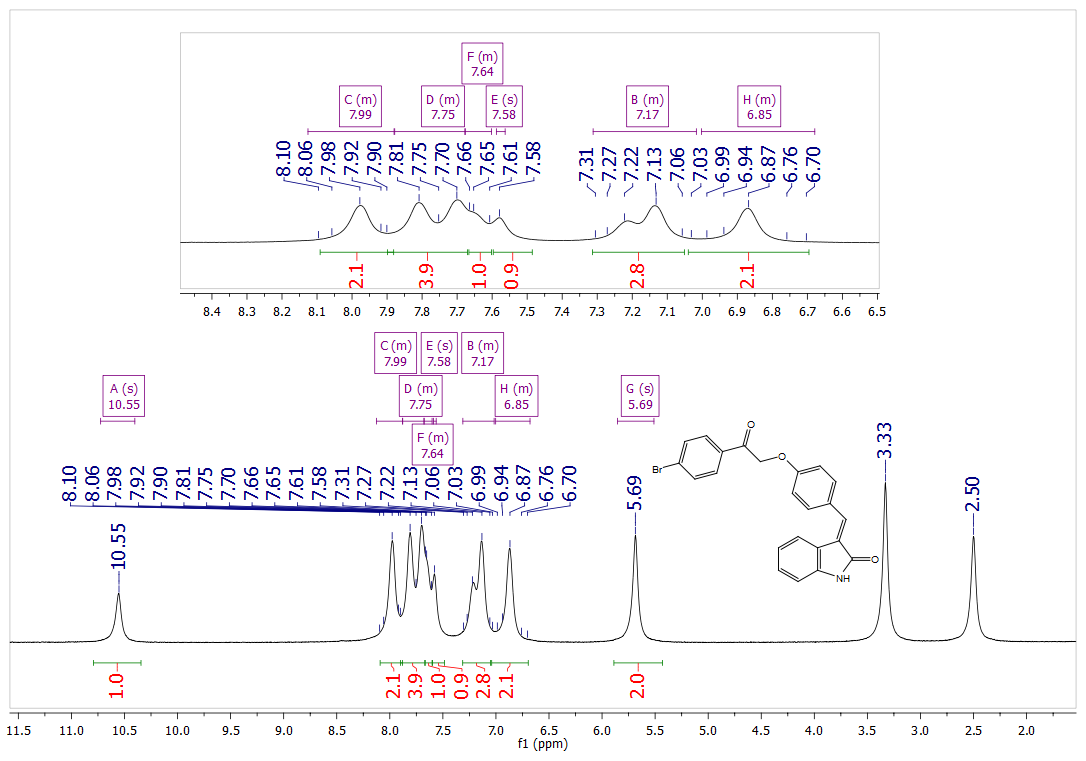


Figure S20. ^1^H NMR spectrum of compound **6f**.


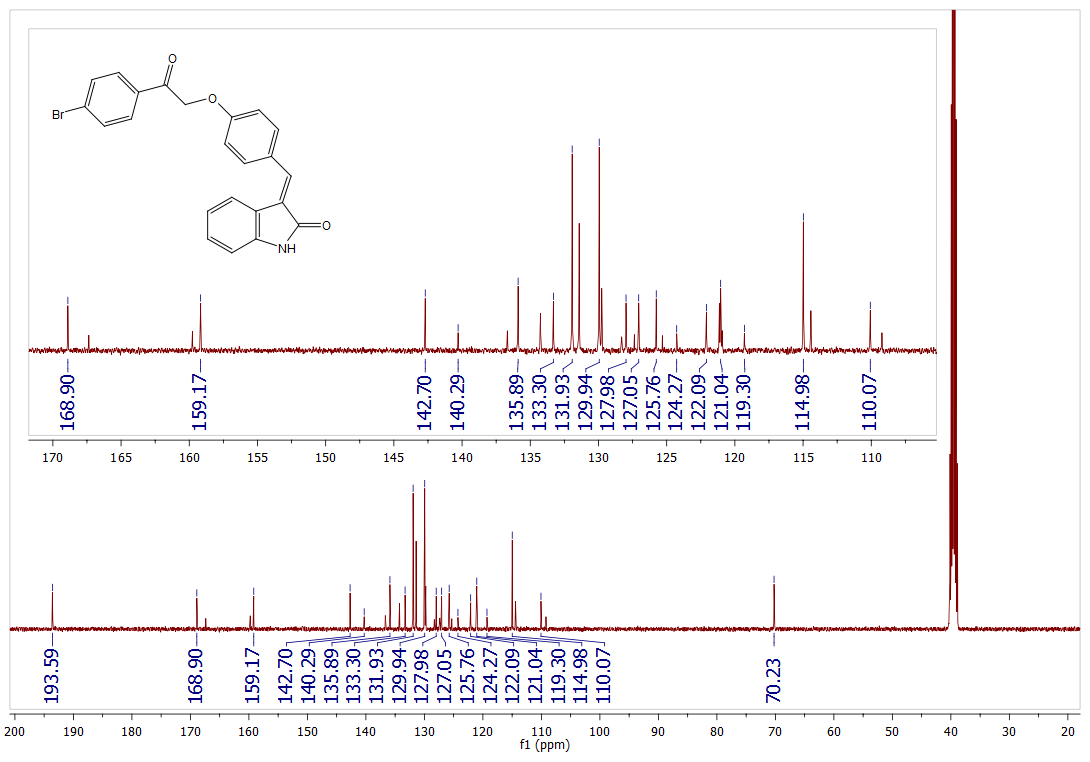


Figure S21. ^13^C NMR spectrum of compound **6f**.


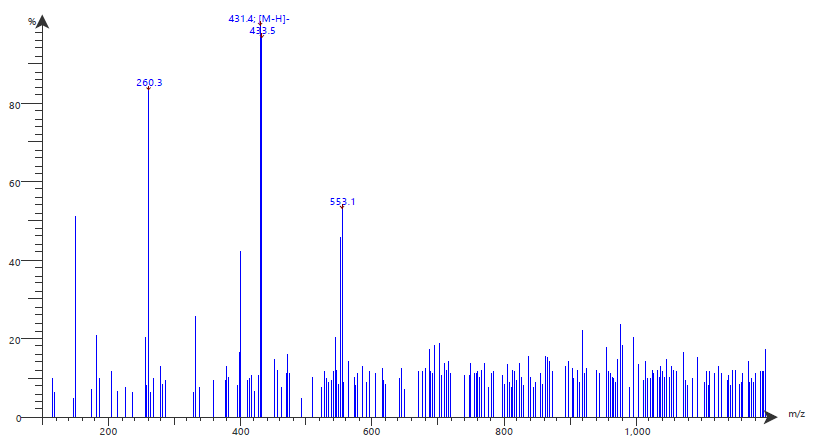


Figure S22. MS (ESI^-^) spectrum of compound **6f**.


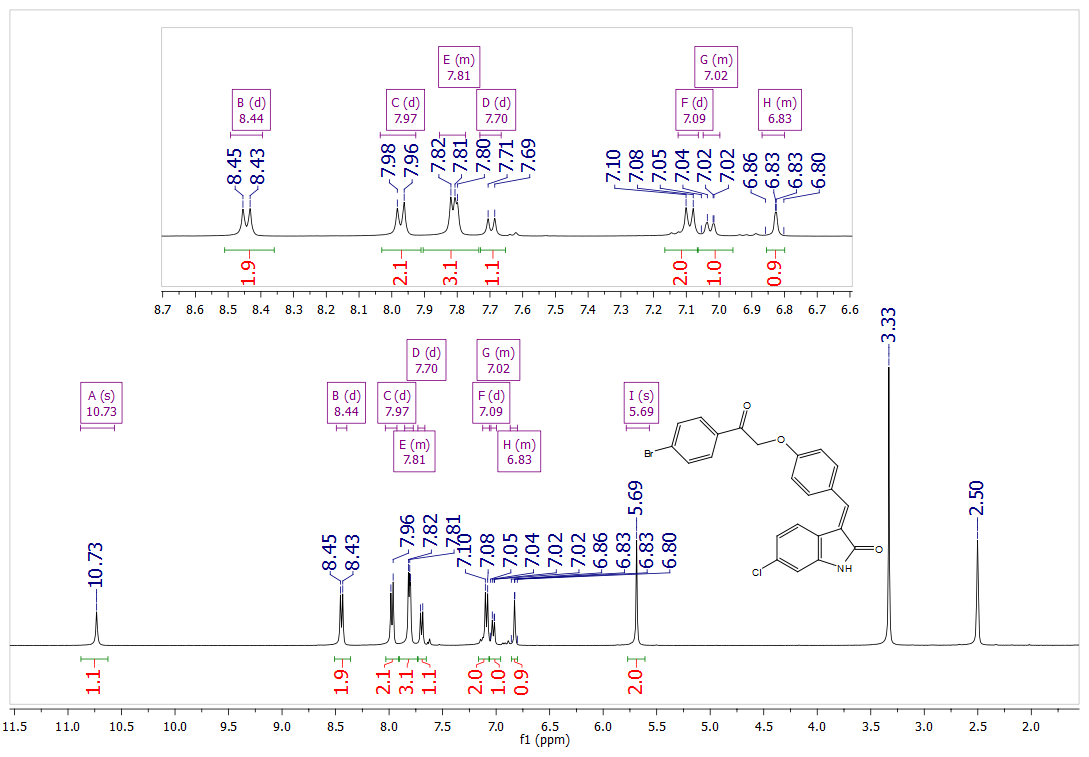


Figure S23. ^1^H NMR spectrum of compound **6g**.


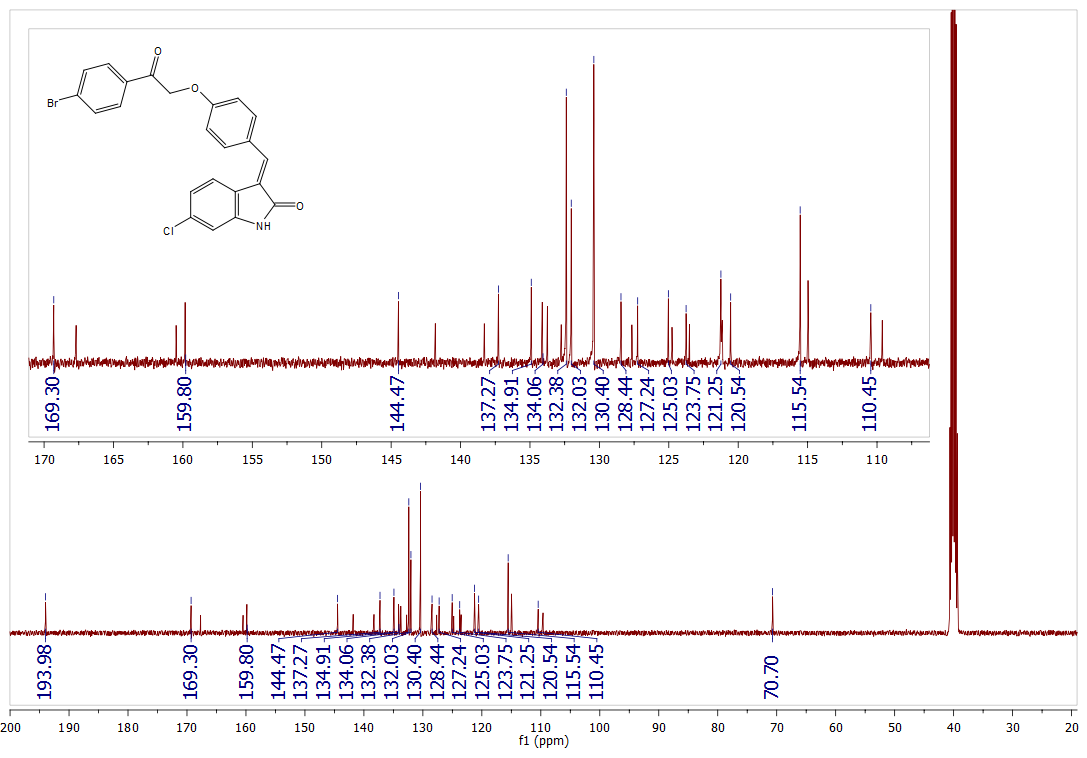


Figure S24. ^13^C NMR spectrum of compound **6g**.


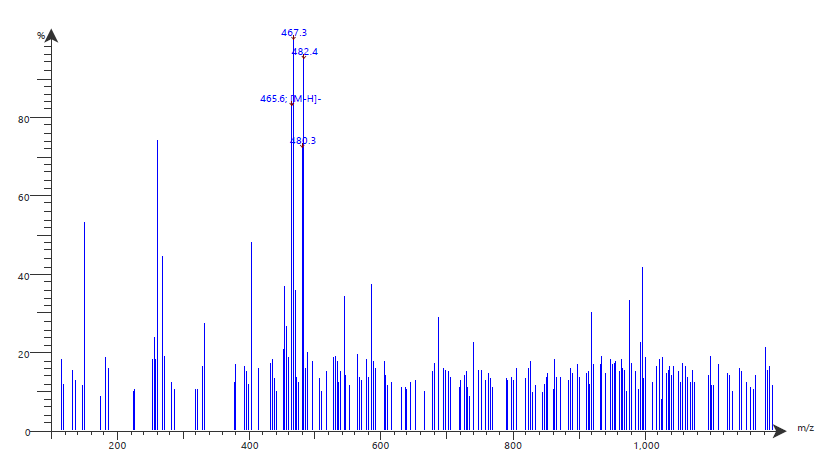


Figure S25. MS (ESI^-^) spectrum of compound **6g**.


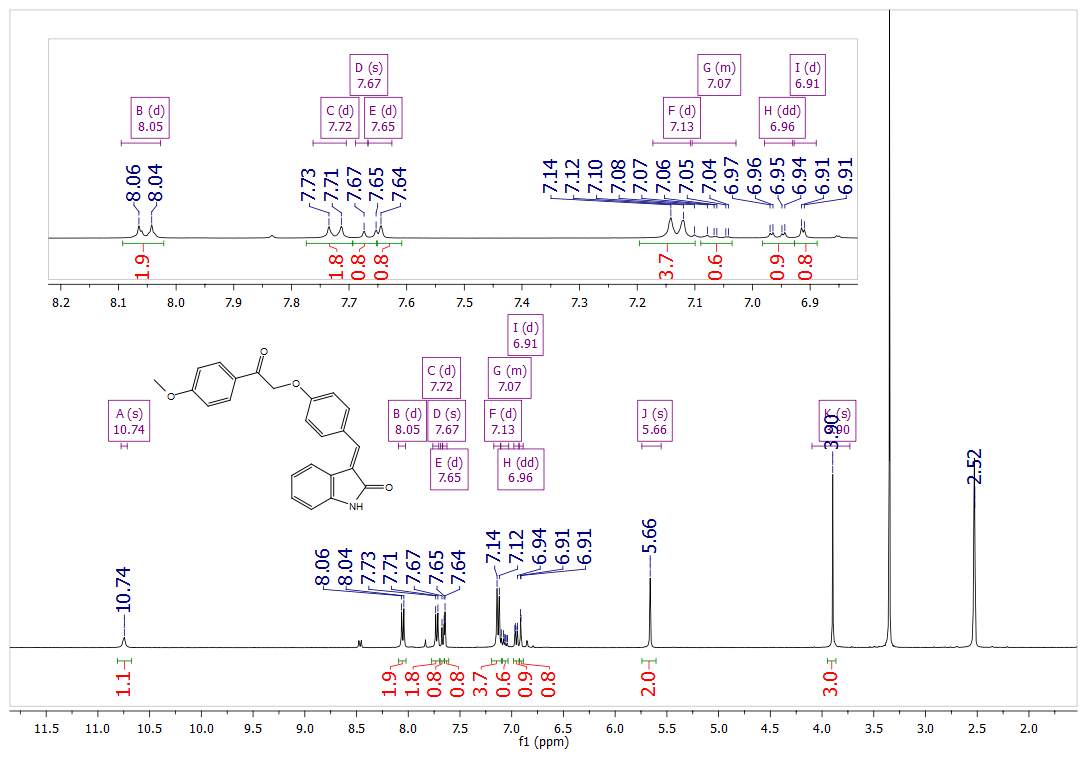


Figure S26. ^1^H NMR spectrum of compound **6h**.


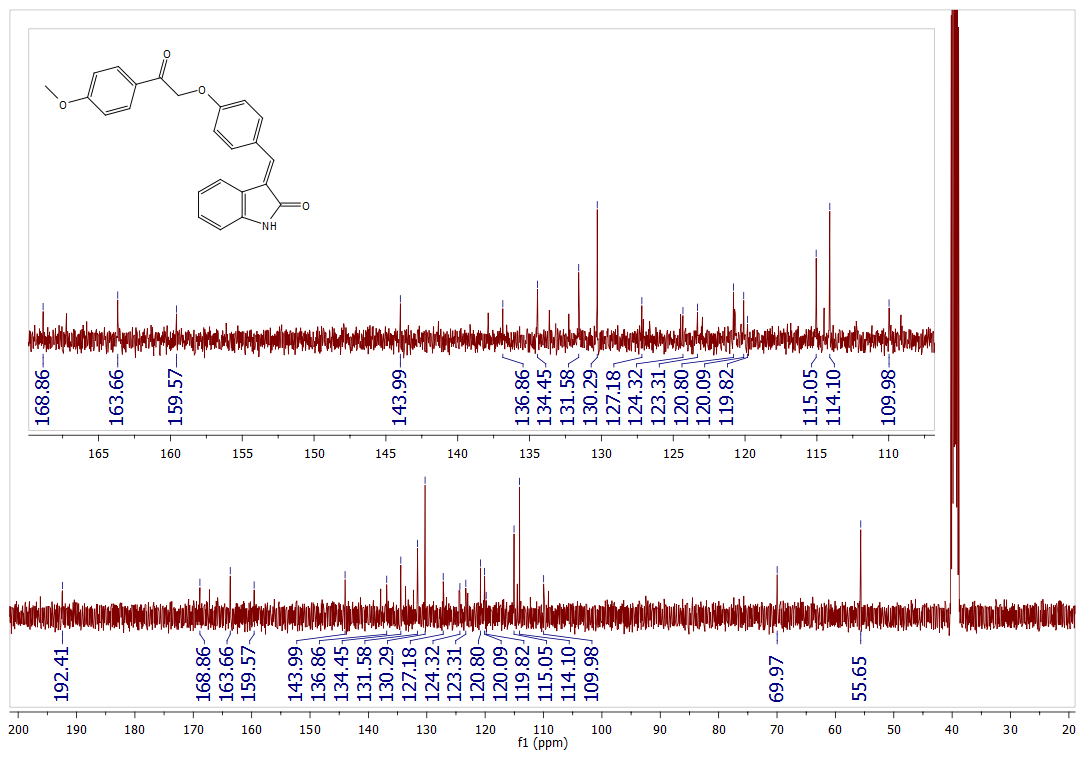


Figure S27. ^13^C NMR spectrum of compound **6h**.


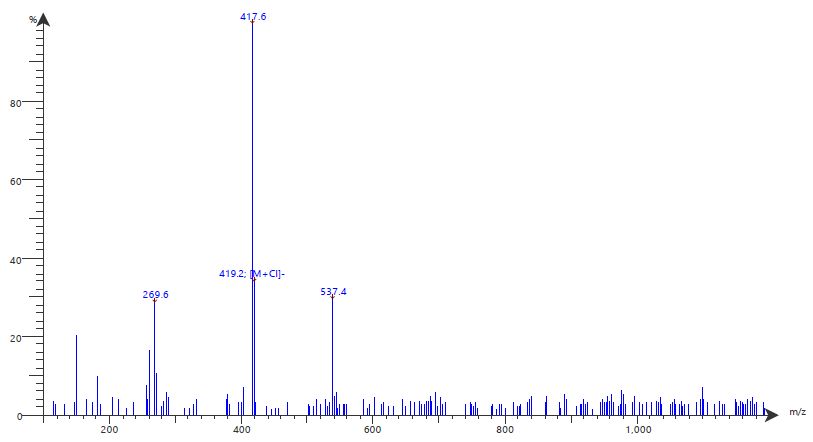


Figure S28. MS (ESI^-^) spectrum of compound **6h**.


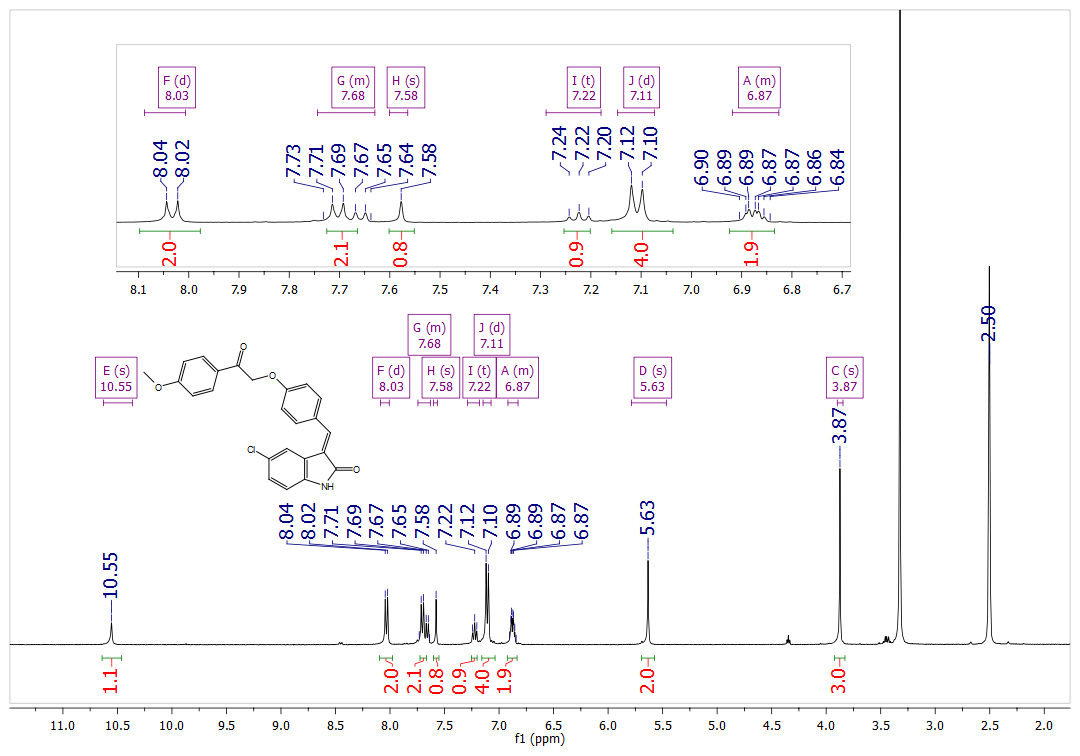


Figure S29. ^1^H NMR spectrum of compound **6i**.


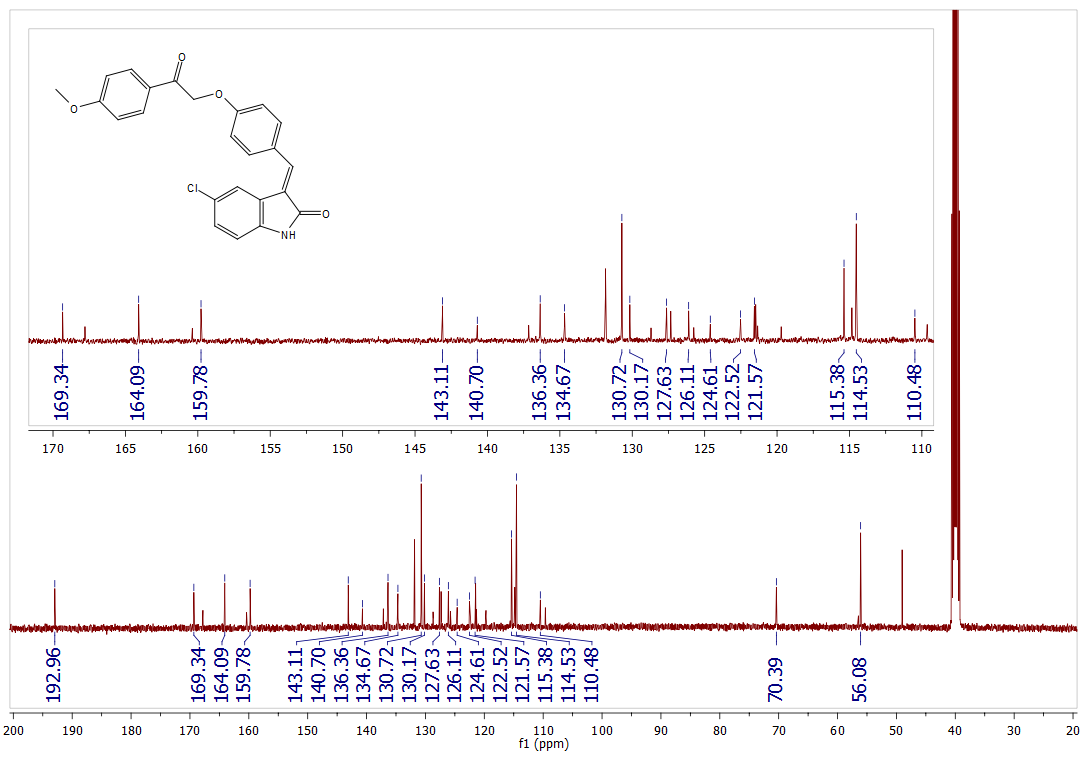


Figure S30. ^13^C NMR spectrum of compound **6i**.

Figure S31. MS (ESI^-^) spectrum of compound **6i**.


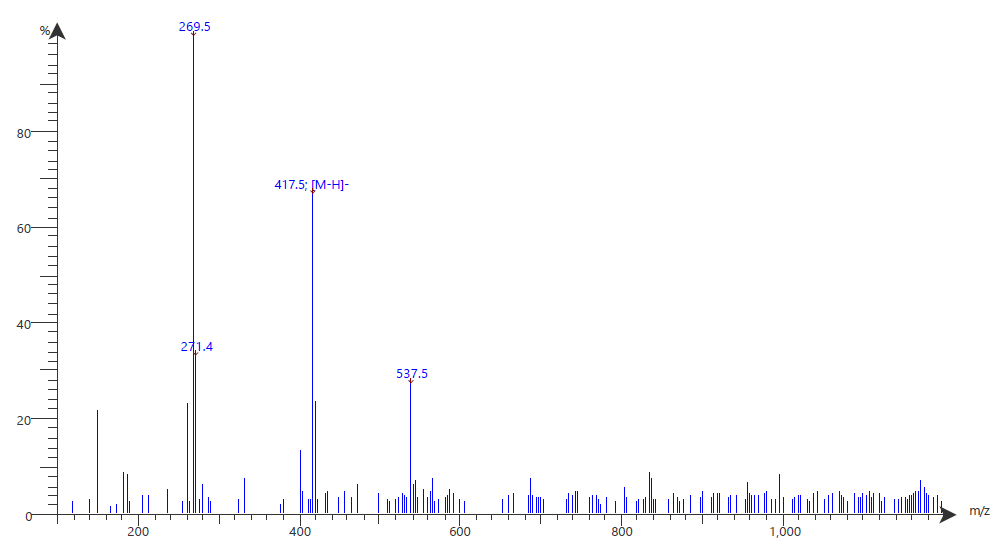


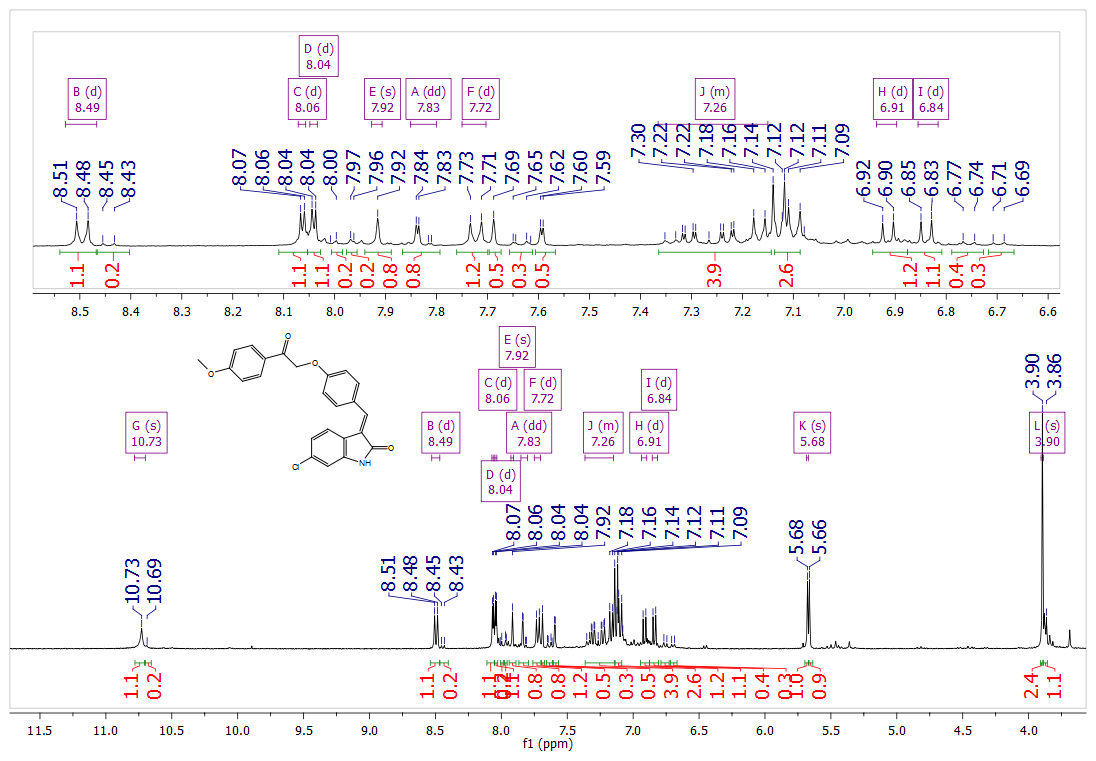


Figure S32. ^1^H NMR spectrum of compound **6j** (**E/Z**).


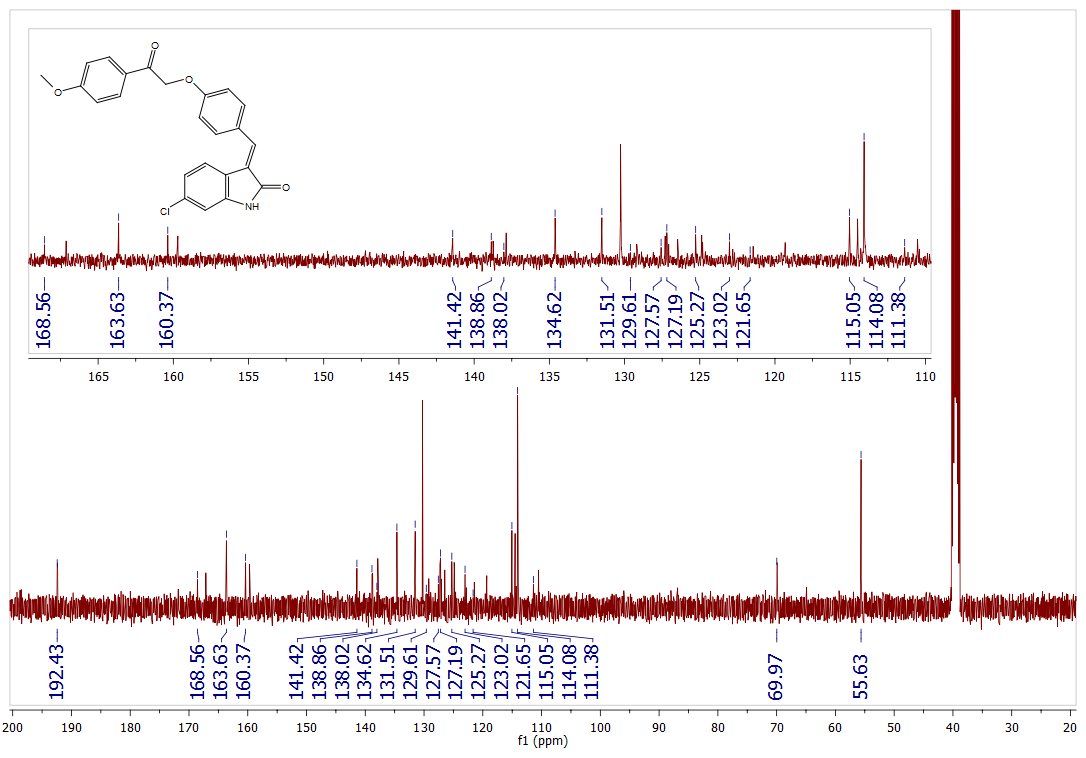


Figure S33. ^13^C NMR spectrum of compound **6j (*E* isomer)**.


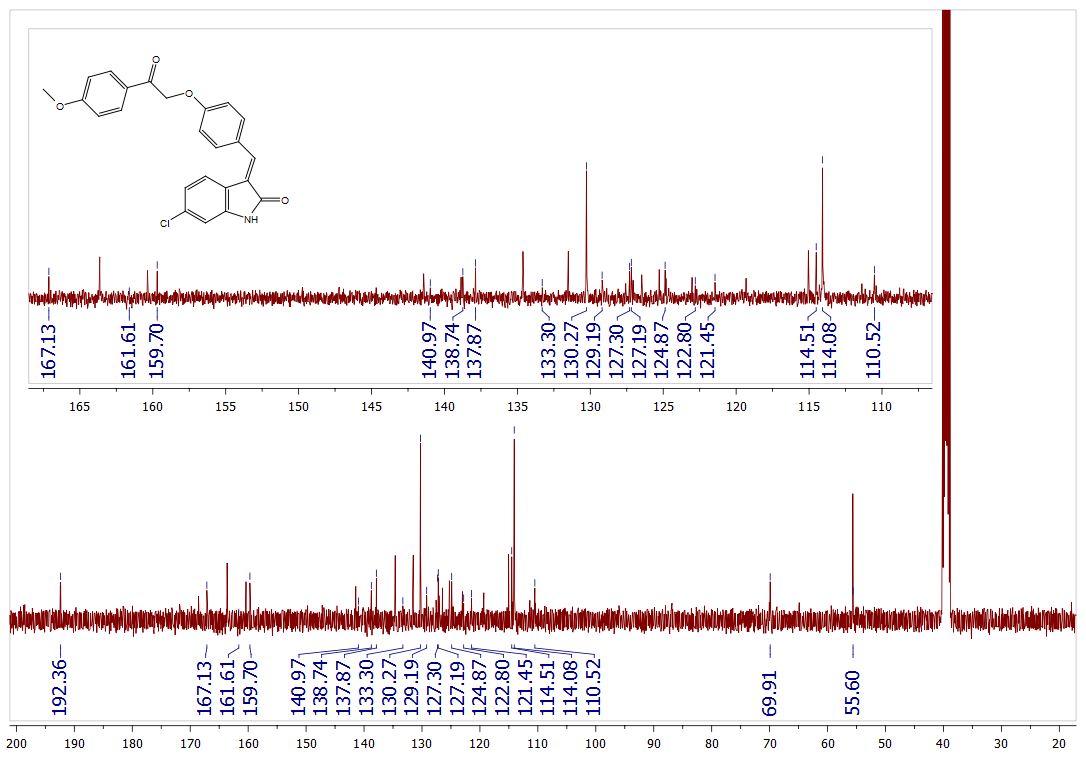


Figure S34. ^13^C NMR spectrum of compound **6j (*Z* isomer)**.


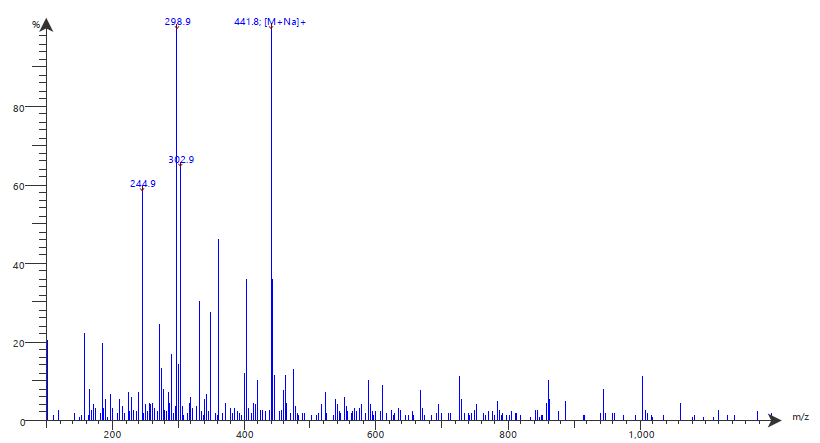


Figure S35. MS (ESI^+^) spectrum of compound **6j**.


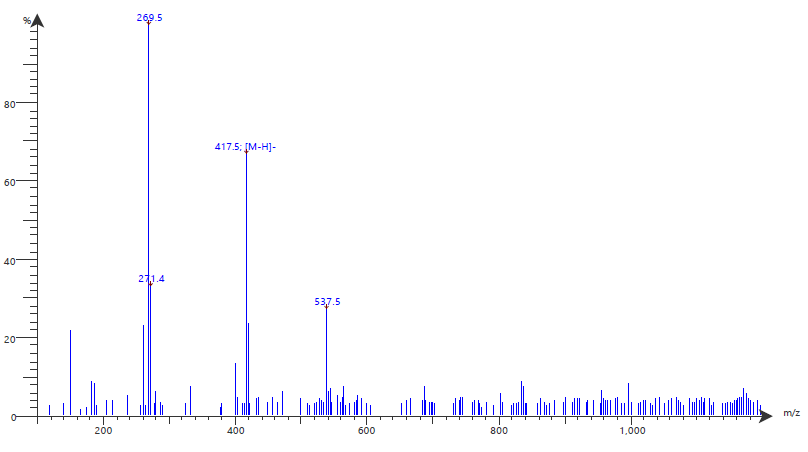

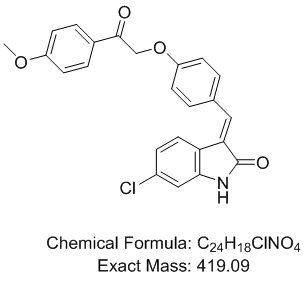


Figure S36. MS (ESI^-^) spectrum of compound **6j**.

**Detailed Results**

|  |  |  |  |  |  |  |  |  |  |  |  |  |
| --- | --- | --- | --- | --- | --- | --- | --- | --- | --- | --- | --- | --- |
| **EGFR** | |  |  |  |  |  |  |  |  |  |  |  |
| code | IC50 | conc | log | %inh | T2 | T1 | ∆T | RFU2 | RFU1 | ∆RFU | slope | K.Activity |
| 6F |  | 10 | 1 | 91 | 30 | 0 | 30 | 8.56 | 0 | 8.56 | 3.333 | 10.27201 |
| 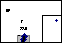   \|  \| \| --- \| |  | 1 | 0 | 72 | 30 | 0 | 30 | 27.79 | 0 | 27.79 | 3.333 | 33.34803 |
|  |  | 0.1 | -1 | 46 | 30 | 0 | 30 | 54.14 | 0 | 54.14 | 3.333 | 64.96806 |
|  |  | 0.01 | -2 | 24 | 30 | 0 | 30 | 76.36 | 0 | 76.36 | 3.333 | 91.63209 |
| EC |  |  |  | 0 | 30 | 0 | 30 | 100 | 0 | 100 | 3.333 | 120 |
|  |  |  |  |  |  |  |  |  |  |  |  |  |
| code | IC50 | conc | log | %inh | T2 | T1 | ∆T | RFU2 | RFU1 | ∆RFU | slope | K.Activity |
| Sunitinib |  | 10 | 1 | 92 | 30 | 0 | 30 | 7.81 | 0 | 7.81 | 3.333 | 9.372009 |
| 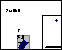   \|  \| \| --- \| |  | 1 | 0 | 79 | 30 | 0 | 30 | 21.02 | 0 | 21.02 | 3.333 | 25.22403 |
|  |  | 0.1 | -1 | 54 | 30 | 0 | 30 | 45.97 | 0 | 45.97 | 3.333 | 55.16406 |
|  |  | 0.01 | -2 | 27 | 30 | 0 | 30 | 72.64 | 0 | 72.64 | 3.333 | 87.16809 |
| EC |  |  |  | 0 | 30 | 0 | 30 | 100 | 0 | 100 | 3.333 | 120 |
|  |  |  |  |  |  |  |  |  |  |  |  |  |

| 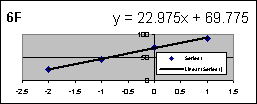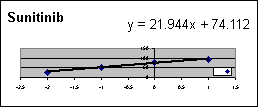   \|  \| \| --- \| |  |  |  |
| --- | --- | --- | --- | --- |
|  |  |  |  |
|  |  |  |  |
|  |  |  |  |
|  |  |  |  |
|  |  |  |  |
|  |  |  |  |
|  |  |  |  |
|  |  |  |  |

Assay : EGFR inhibition assay

Samples : 01 compounds

Date : 03-03-2022

Reader : Tecan Spark Reader

Kit used : ---.

Solvent : DMSO

**Lab Report**

| **ser** | **Compound** | | **EGFR** | **SD**  ± |
| --- | --- | --- | --- | --- |
|  | **code** | **MW**  **g/mol** | **IC50**  **uM** |  |
| 1 | **6F** | **433.03** | **1.380** | 0.008 |
| 2 | **Sunitinib** | **398.47** | **0.080** | 0.005 |

Figure S37. Epidermal growth factor receptor (EGFR) inhibition assay for compound **6f**.

**Detailed Results**

**VEGFR-2**

|  |  |  |  |  |  |  |  |  |  |  |  |  |
| --- | --- | --- | --- | --- | --- | --- | --- | --- | --- | --- | --- | --- |
| code | IC50 | conc | log | %inh | T2 | T1 | ∆T | RFU2 | RFU1 | ∆RFU | slope | K.Activity |
| 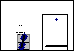   \| **6F** \| \| --- \| |  | 10 | 1 | 88.74 | 30 | 0 | 30 | 11.26 | 0 | 11.26 | 3.3333 | 13.5121 |
|  |  | 1 | 0 | 73.94 | 30 | 0 | 30 | 26.06 | 0 | 26.06 | 3.3333 | 31.2723 |
|  |  | 0.1 | -1 | 36.13 | 30 | 0 | 30 | 63.87 | 0 | 63.87 | 3.3333 | 76.6448 |
|  |  | 0.01 | -2 | 12.36 | 30 | 0 | 30 | 87.64 | 0 | 87.64 | 3.3333 | 105.169 |
| EC |  |  |  | 0 | 30 | 0 | 30 | 100 | 0 | 100 | 3.3333 | 120 |
|  |  |  |  |  |  |  |  |  |  |  |  |  |
| code | IC50 | conc | log | %inh | T2 | T1 | ∆T | RFU2 | RFU1 | ∆RFU | slope | K.Activity |
| 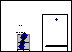   \| **Sunitinib** \| \| --- \| |  | 10 | 1 | 90.41 | 30 | 0 | 30 | 9.59 | 0 | 9.59 | 3.3333 | 11.5081 |
|  |  | 1 | 0 | 77.29 | 30 | 0 | 30 | 22.71 | 0 | 22.71 | 3.3333 | 27.2523 |
|  |  | 0.1 | -1 | 45.17 | 30 | 0 | 30 | 54.83 | 0 | 54.83 | 3.3333 | 65.7967 |
|  |  | 0.01 | -2 | 23.07 | 30 | 0 | 30 | 76.93 | 0 | 76.93 | 3.3333 | 92.3169 |
| EC |  |  |  | 0 | 30 | 0 | 30 | 100 | 0 | 100 | 3.3333 | 120 |
|  |  |  |  |  |  |  |  |  |  |  |  |  |

| 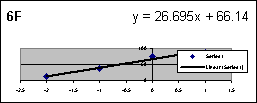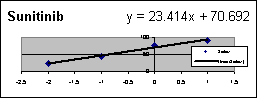   \|  \| \| --- \| |  |  |  |
| --- | --- | --- | --- | --- |
|  |  |  |  |
|  |  |  |  |
|  |  |  |  |
|  |  |  |  |
|  |  |  |  |
|  |  |  |  |
|  |  |  |  |
|  |  |  |  |

Assay : VEGFR2 enzyme assay

Samples : 01 compounds.

Date : 03/03/2022

Reader : Tecan –spark reader BIOLINE ELISA READER wl 450 nm

Solvent : DMSO

**Lab Report**

| **ser** | **Compound** | |  | **SD**  ± |
| --- | --- | --- | --- | --- |
|  | **code** | **MW**  **g/mol** | **IC50**  **uM** |  |
| 1 | **6F** | **433.03** | 5.750 | 0.011 |
| 2 | **Sunitinib** | **398.47** | 0.328 | 0.006 |

Figure S38. Vascular endothelial growth factor (VEGFR-2) inhibition assay for compound **6f**.

**Detailed Results**

| **PDGFRβ** | |  |  |  |  |  |  |  |  |  |  |  |
| --- | --- | --- | --- | --- | --- | --- | --- | --- | --- | --- | --- | --- |
|  |  |  |  |  |  |  |  |  |  |  |  |  |
| code | IC50 | conc | log | %inh | T2 | T1 | ∆T | RFU2 | RFU1 | ∆RFU | slope | K.Activity |
| 6F |  | 10 | 1 | 88.18 | 30 | 0 | 30 | 11.82 | 0 | 11.82 | 3.3333 | 14.1841 |
| 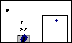   \|  \| \| --- \| |  | 1 | 0 | 70.36 | 30 | 0 | 30 | 29.64 | 0 | 29.64 | 3.3333 | 35.5684 |
|  |  | 0.1 | -1 | 40.59 | 30 | 0 | 30 | 59.41 | 0 | 59.41 | 3.3333 | 71.2927 |
|  |  | 0.01 | -2 | 25.63 | 30 | 0 | 30 | 74.37 | 0 | 74.37 | 3.3333 | 89.2449 |
| EC |  |  |  | 0 | 30 | 0 | 30 | 100 | 0 | 100 | 3.3333 | 120 |
|  |  |  |  |  |  |  |  |  |  |  |  |  |
| code | IC50 | conc | log | %inh | T2 | T1 | ∆T | RFU2 | RFU1 | ∆RFU | slope | K.Activity |
| Sunitinib |  | 10 | 1 | 91.45 | 30 | 0 | 30 | 8.55 | 0 | 8.55 | 3.3333 | 10.2601 |
| 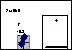   \|  \| \| --- \| |  | 1 | 0 | 76.93 | 30 | 0 | 30 | 23.07 | 0 | 23.07 | 3.3333 | 27.6843 |
|  |  | 0.1 | -1 | 47.51 | 30 | 0 | 30 | 52.49 | 0 | 52.49 | 3.3333 | 62.9886 |
|  |  | 0.01 | -2 | 35.11 | 30 | 0 | 30 | 64.89 | 0 | 64.89 | 3.3333 | 77.8688 |
| EC |  |  |  | 0 | 30 | 0 | 30 | 100 | 0 | 100 | 3.3333 | 120 |
|  |  |  |  |  |  |  |  |  |  |  |  |  |


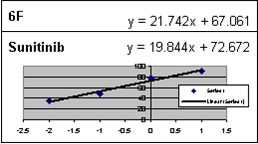


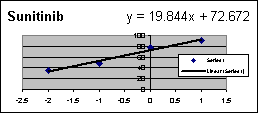

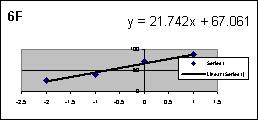

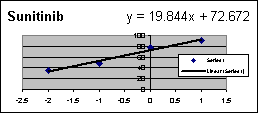

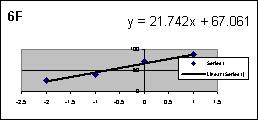


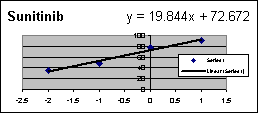

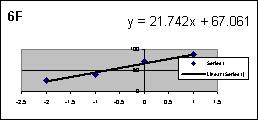


Assay : PDGFR- ẞ enzyme assay

Samples : 01 compounds.

Date : 03/03/2022

Reader : Tecan –spark reader BIOLINE ELISA READER wl 450 nm

Solvent : DMSO

**Lab Report**

| **ser** | **Compound** | |  | **SD**  ± |
| --- | --- | --- | --- | --- |
|  | **code** | **MW**  **g/mol** | **IC50**  **uM** |  |
| 1 | **6F** | **433.03** | 3.180 | 0.007 |
| 2 | **Sunitinib** | **398.47** | 0.180 | 0.003 |

Figure S39. platelet-derived growth factor-ẞ (PDGF-ẞ) inhibition assay for compound **6f**.

**Detailed Results**

|  |  |  |  |  |  |  |  |  |  |  |  |  |
| --- | --- | --- | --- | --- | --- | --- | --- | --- | --- | --- | --- | --- |
| Tub |  |  |  |  |  |  |  |  |  |  |  |  |
| code | IC50 | conc | log | %inh | T2 | T1 | ∆T | RFU2 | RFU1 | ∆RFU | slope | K.Activity |
| 6F |  | 100 | 2 | 80 | 30 | 0 | 30 | 0.087 | 0 | 0.087 | 0.0142 | 24.50704 |
| 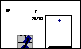   \|  \| \| --- \| |  | 10 | 1 | 56 | 30 | 0 | 30 | 0.186 | 0 | 0.186 | 0.0142 | 52.39437 |
|  |  | 1 | 0 | 15 | 30 | 0 | 30 | 0.361 | 0 | 0.361 | 0.0142 | 101.6901 |
|  |  | 0.1 | -1 | 7 | 30 | 0 | 30 | 0.395 | 0 | 0.395 | 0.0142 | 111.2676 |
| EC |  |  |  | 0 | 30 | 0 | 30 | 0.426 | 0 | 0.426 | 0.0142 | 120 |
|  |  |  |  |  |  |  |  |  |  |  |  |  |
| code | IC50 | conc.uM | log conc | %inh | T2 | T1 | ∆T | RFU2 | RFU1 | ∆RFU | slope | K.Activity |
| CA-4 |  | 100 | 2 | 85 | 30 | 0 | 30 | 0.064 | 0 | 0.064 | 0.0142 | 18.02817 |
| 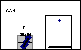   \|  \| \| --- \| |  | 10 | 1 | 58 | 30 | 0 | 30 | 0.177 | 0 | 0.177 | 0.0142 | 49.85915 |
|  |  | 1 | 0 | 38 | 30 | 0 | 30 | 0.262 | 0 | 0.262 | 0.0142 | 73.80282 |
|  |  | 0.1 | -1 | 24 | 30 | 0 | 30 | 0.322 | 0 | 0.322 | 0.0142 | 90.70423 |
| EC |  |  |  | 0 | 30 | 0 | 30 | 0.426 | 0 | 0.426 | 0.0142 | 120 |
|  |  |  |  |  |  |  |  |  |  |  |  |  |

| 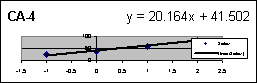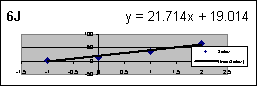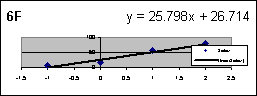 |  |  |  |
| --- | --- | --- | --- |
|  |  |  |  |
|  |  |  |  |
|  |  |  |  |
|  |  |  |  |
|  |  |  |  |
|  |  |  |  |
|  |  |  |  |
|  |  |  |  |

Assay : Tubulin enzyme assay

Samples : 01 compound.

Date : 03/03/2022

**Reader** : Tecan-Spark reader

Kit used : Cloud-clone corp) SEB870Hu EIA Kit For Tubulin Beta (TUBb).

**Solvent** : DMSO

**Lab Report**

**0**

**5**

**10**

**15**

**20**

**25**

**30**

**ug/ml**

**6**

**F**

**CA**

**-**

**4**

**Tub**

**.**

**polymerization**

**_**

**IC**

**50**

**Tub**

| **ser** | **Compound** | | | **Tubulin polymerization Inhibition** | **SD**  **±** |
| --- | --- | --- | --- | --- | --- |
|  | **ID** | **M.W**  **g/mol** | **conc** | **IC50**  **uM** |  |
| 1 | **6F** | **433.03** |  | 7.99 | 0.49 |
| 3 | **CA-4** | **334.36** |  | 2.64 | 0.16 |

Figure S40. Tubulin polymerization assay for compound **6f**.


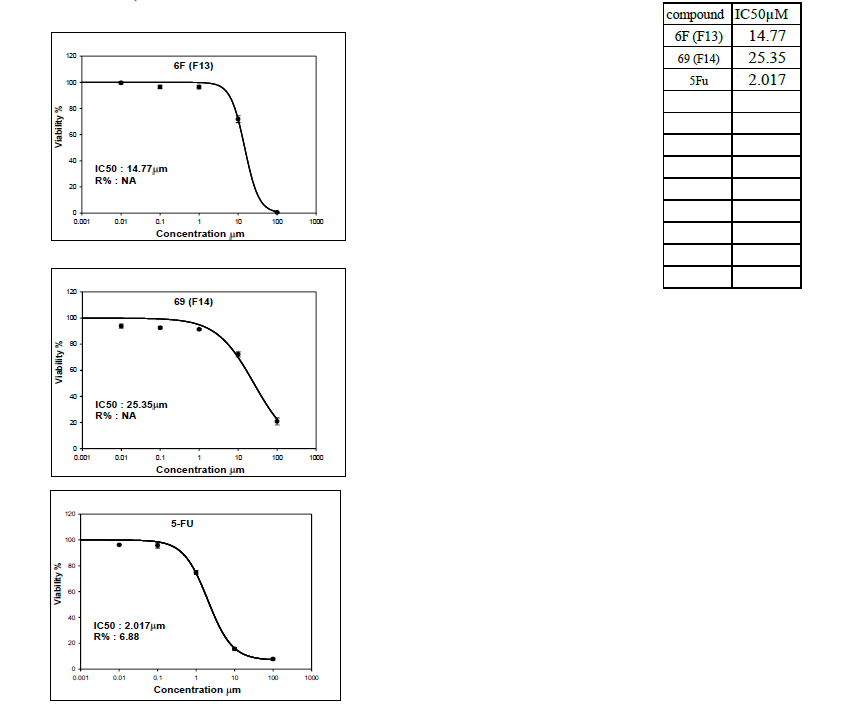


Figure S41. IC_50_ of compounds **6f ,6g,** and 5-FU against MCF-7 using SRB assay.


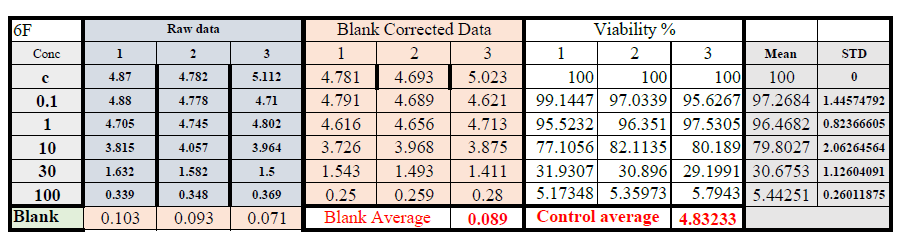

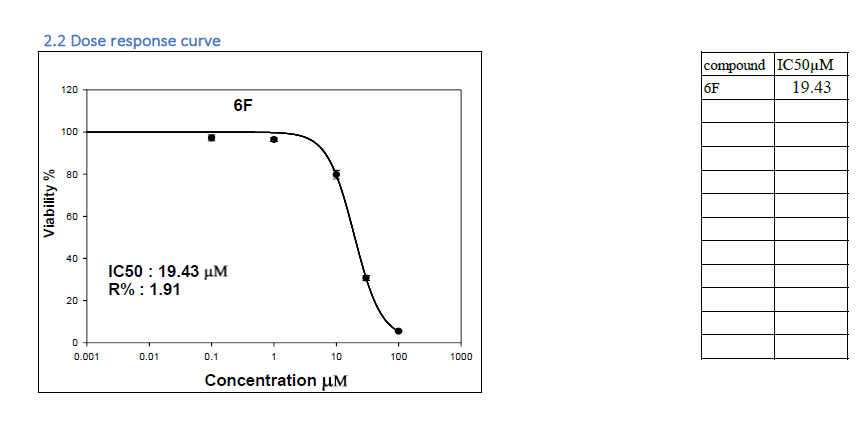


Figure S42. IC_50_ of compounds **6f** against HSF cells using SRB assay.


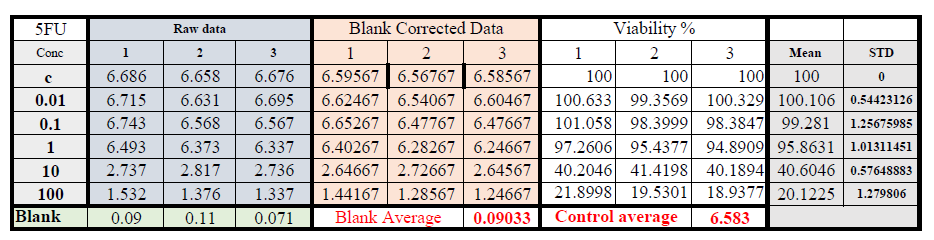

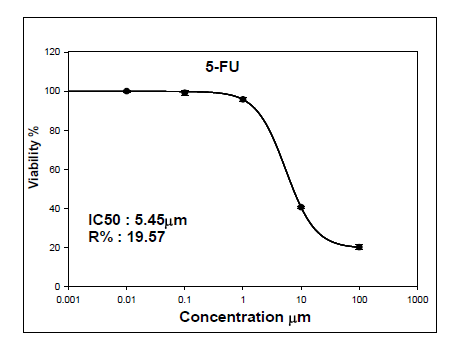


Figure S43. IC_50_ of compounds **5-Fluorouracil (5-FU)** against HSF cells using SRB assay.


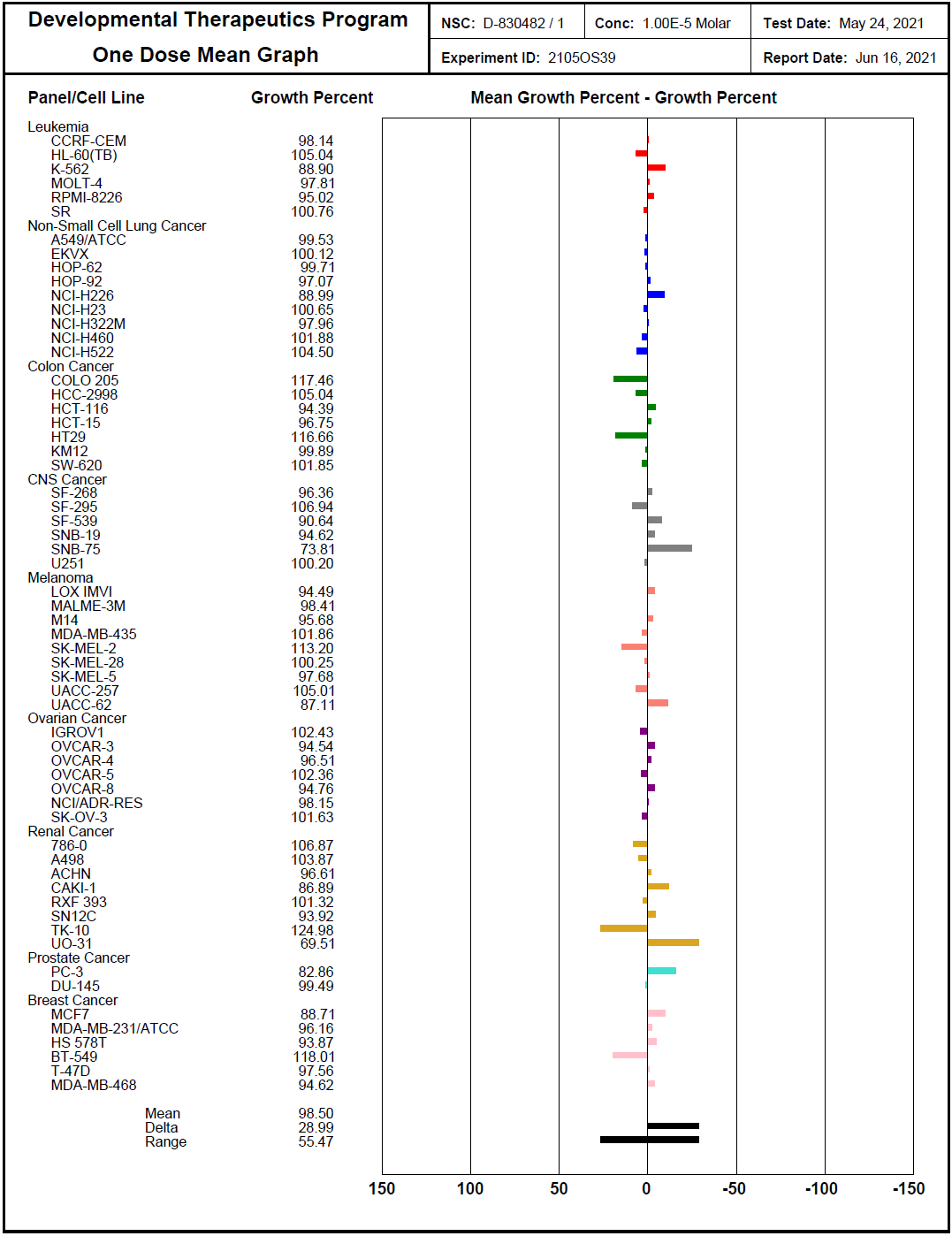


Figure S44. One- dose NCI growth (%) and mean graph of compound **6a**.


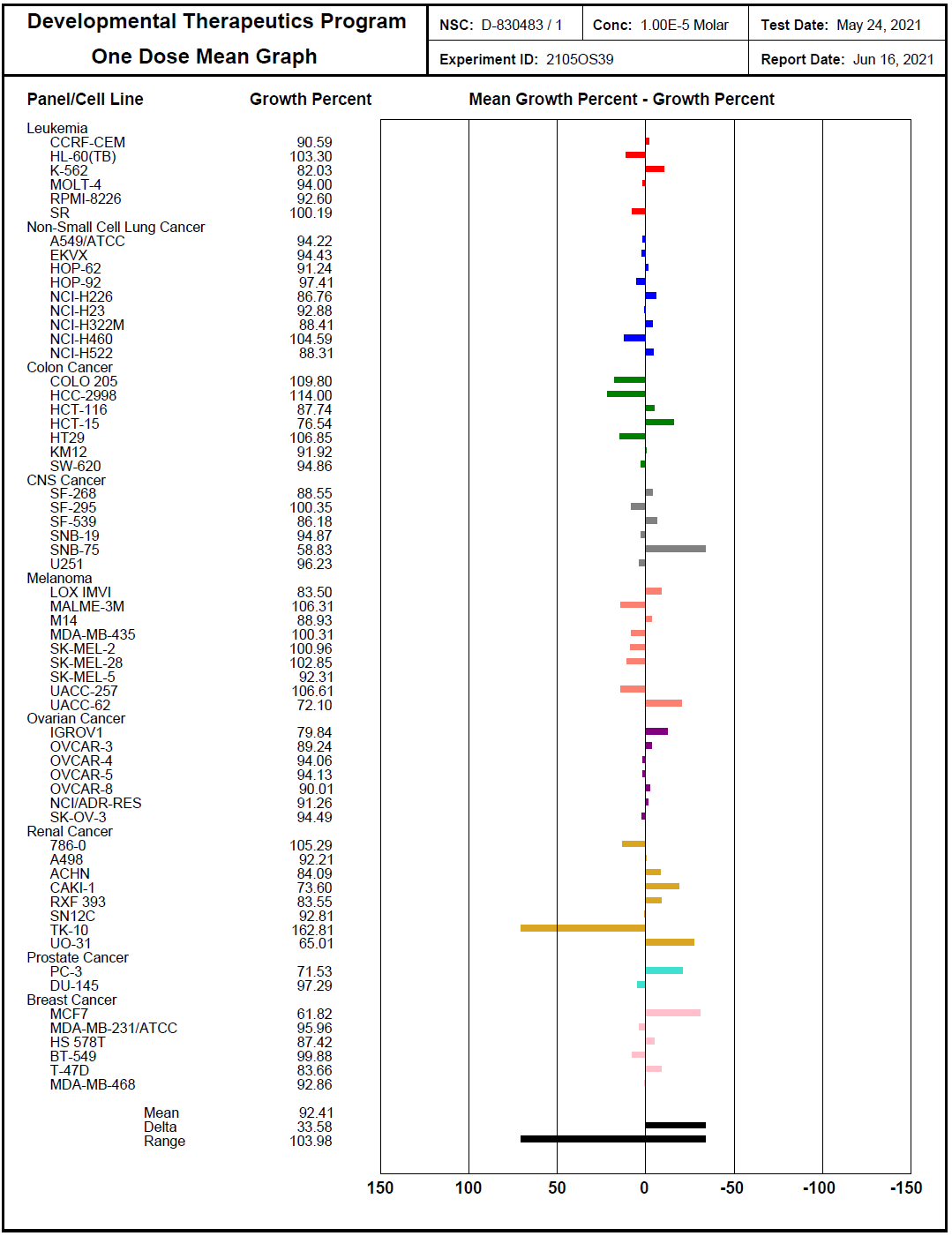


Figure S45. One- dose NCI growth (%) and mean graph of compound **6b**.


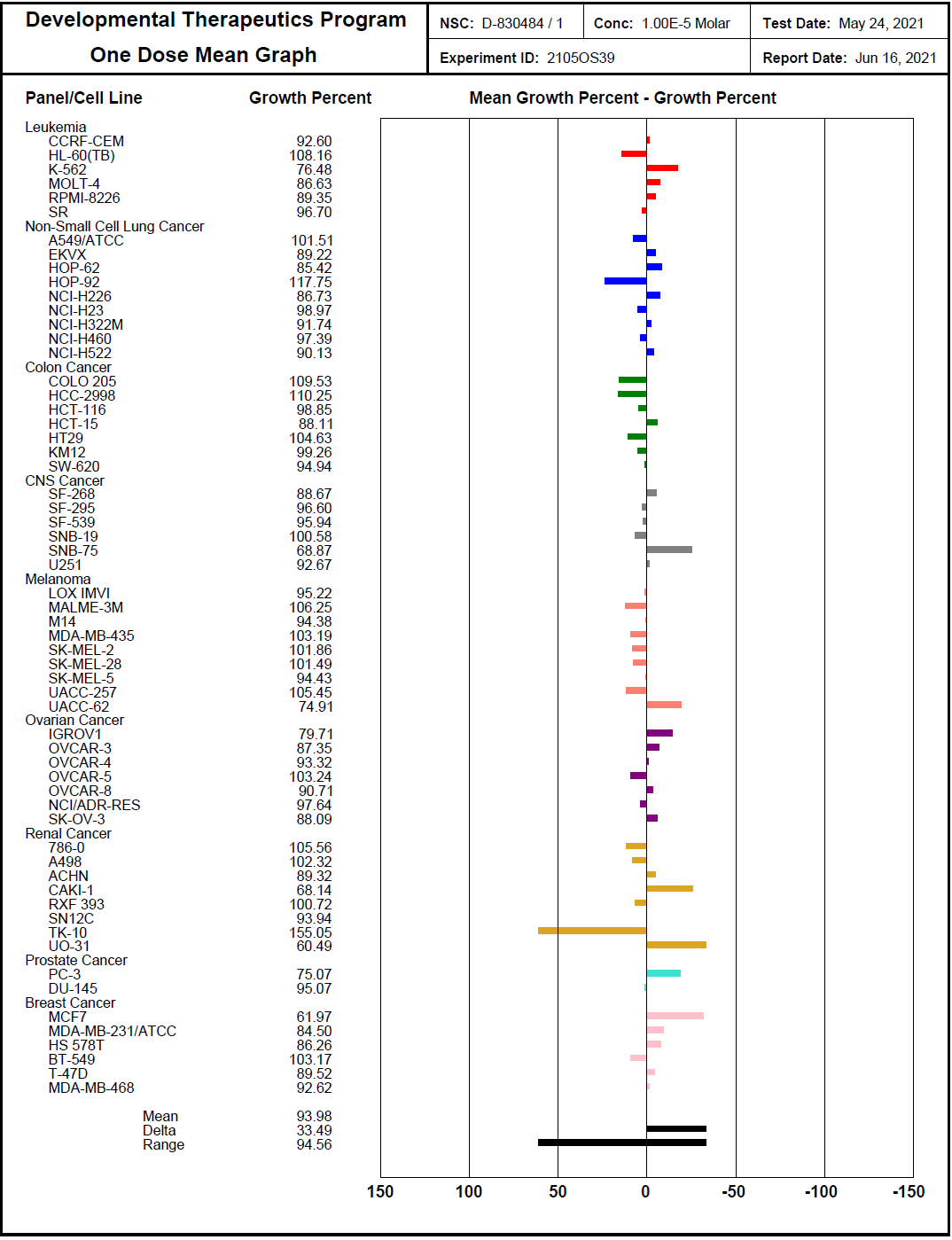


Figure S46. One- dose NCI growth (%) and mean graph of compound **6c**.


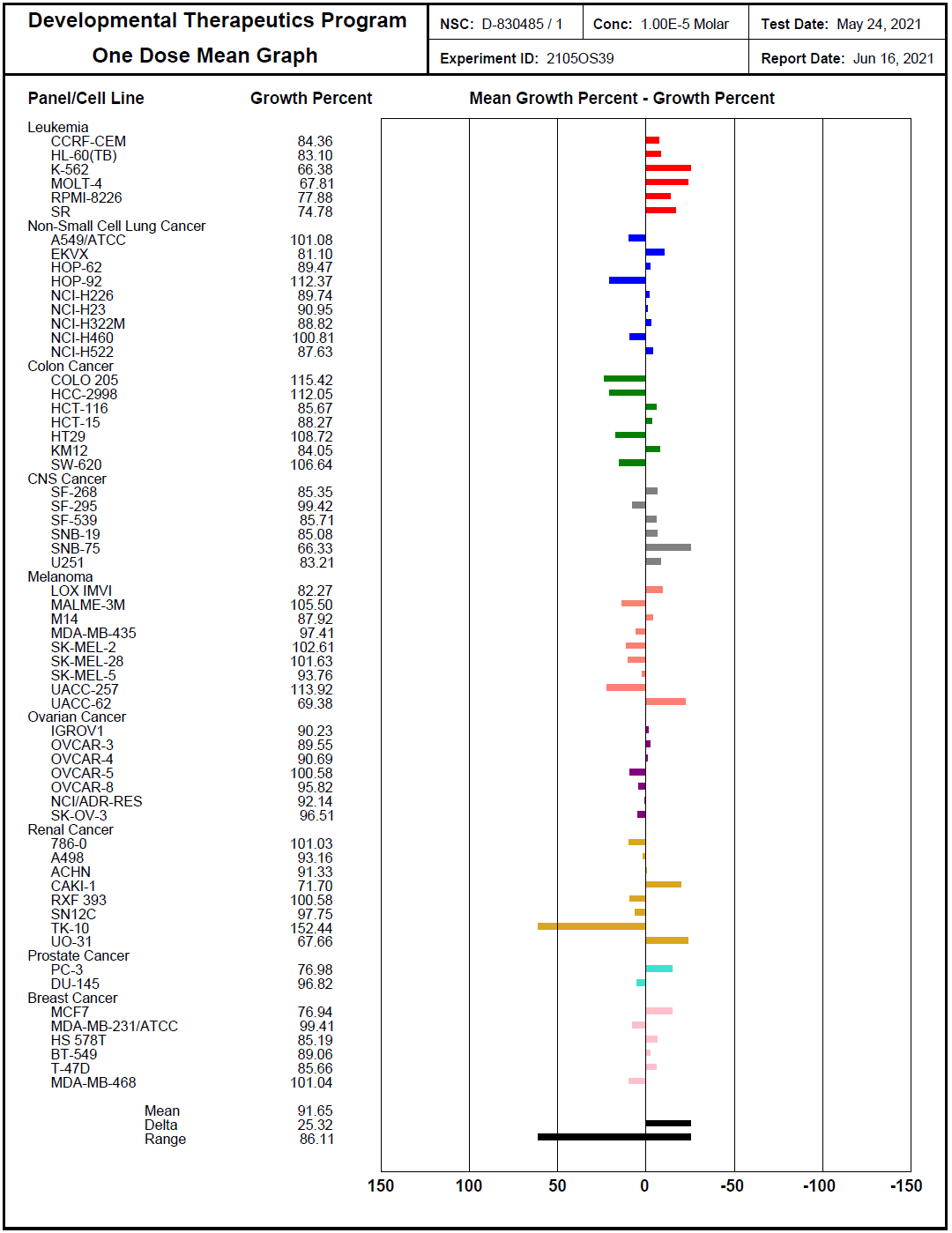


Figure S47. One- dose NCI growth (%) and mean graph of compound **6d**.


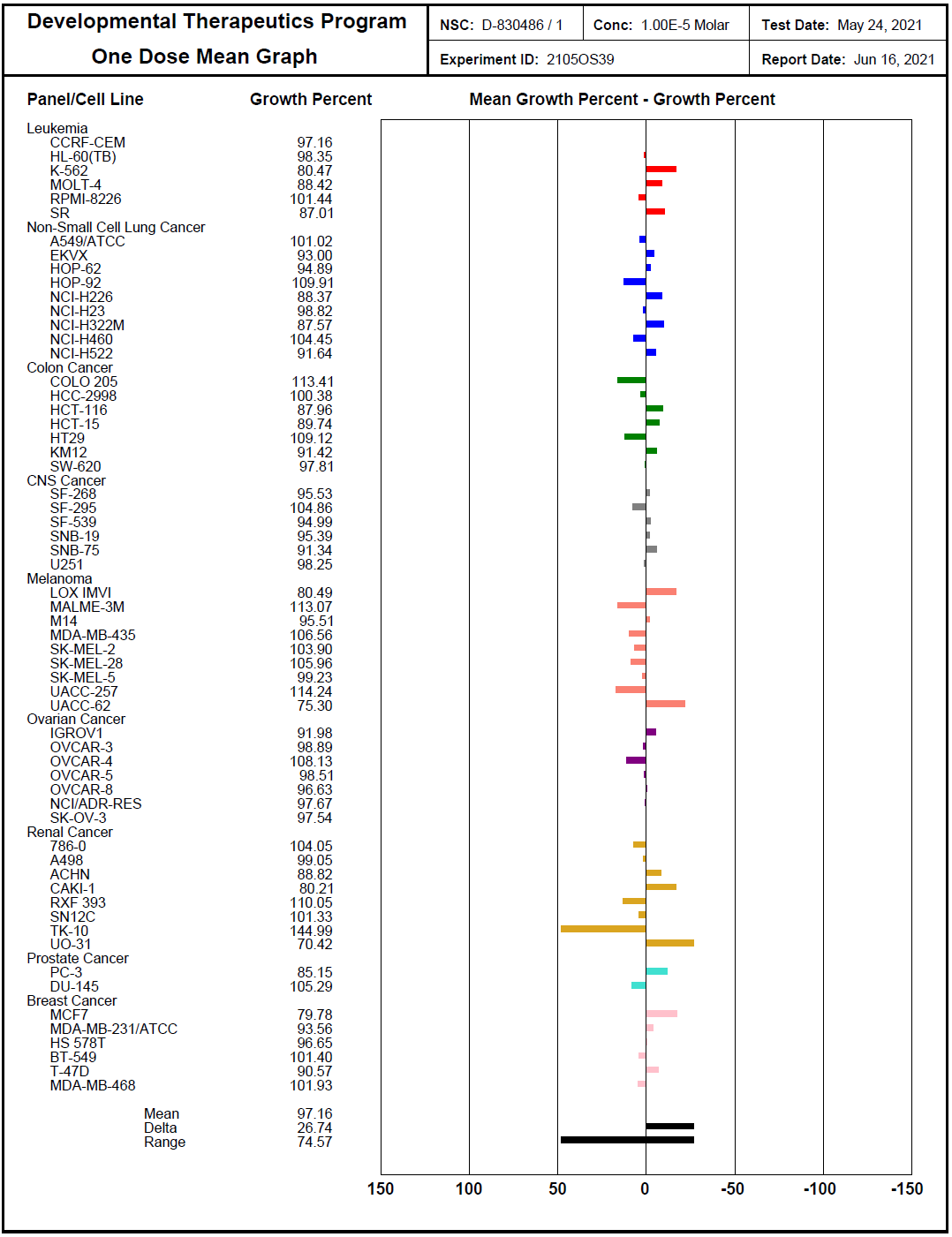


Figure S48. One- dose NCI growth (%) and mean graph of compound **6e**.


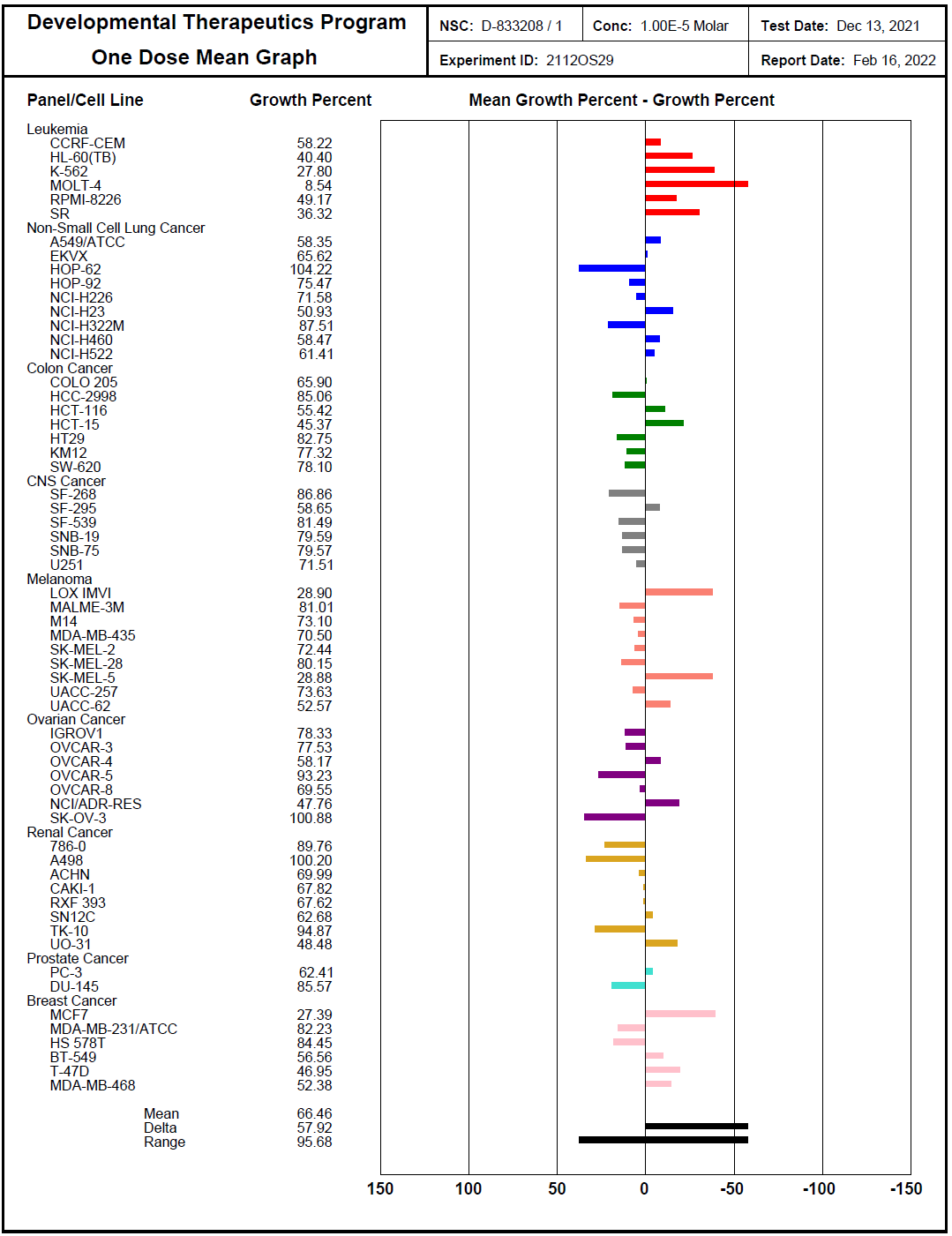


Figure S49. One- dose NCI growth (%) and mean graph of compound **6f**.


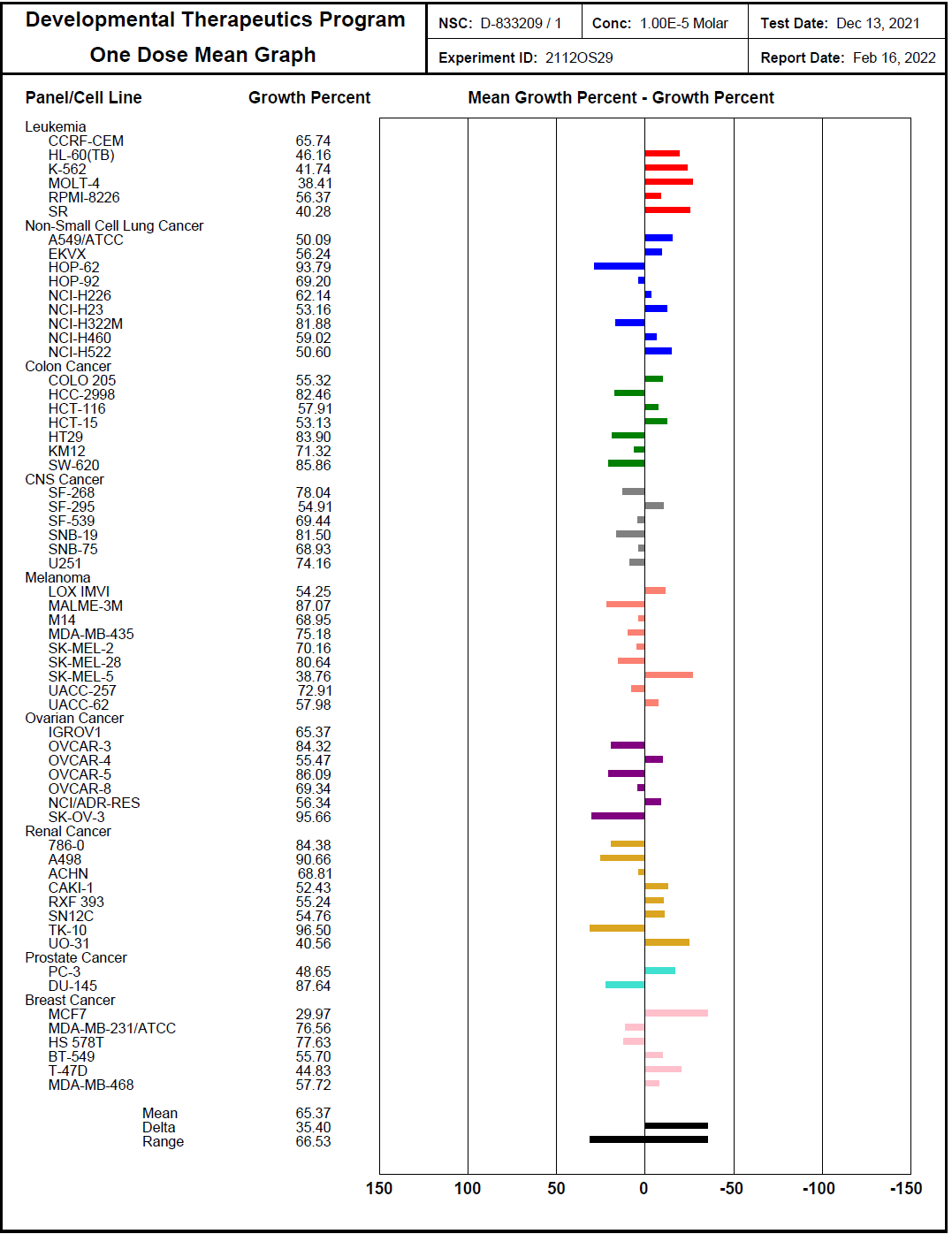


Figure S50. One- dose NCI growth (%) and mean graph of compound **6g**.


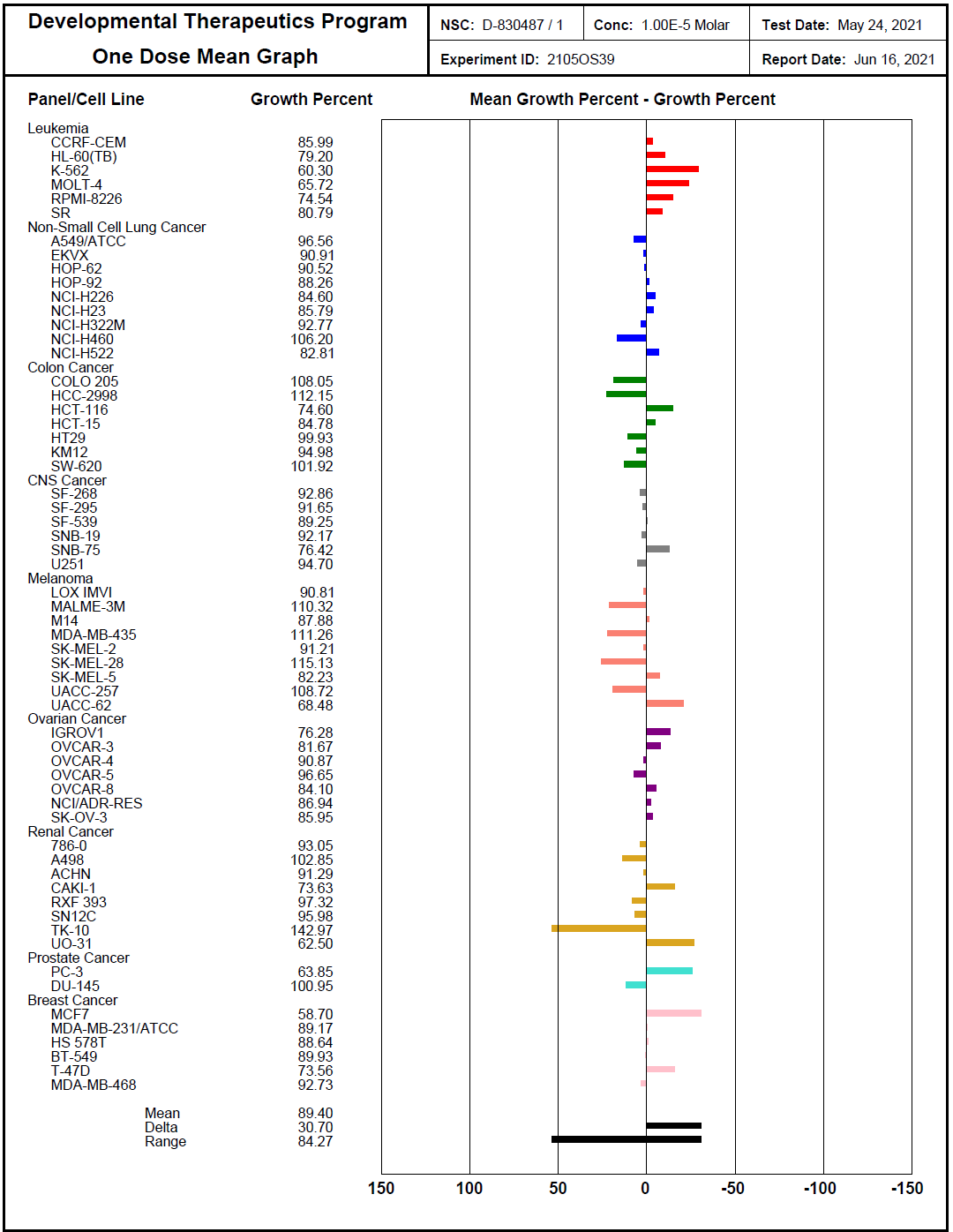


Figure S51. One- dose NCI growth (%) and mean graph of compound **6h**.


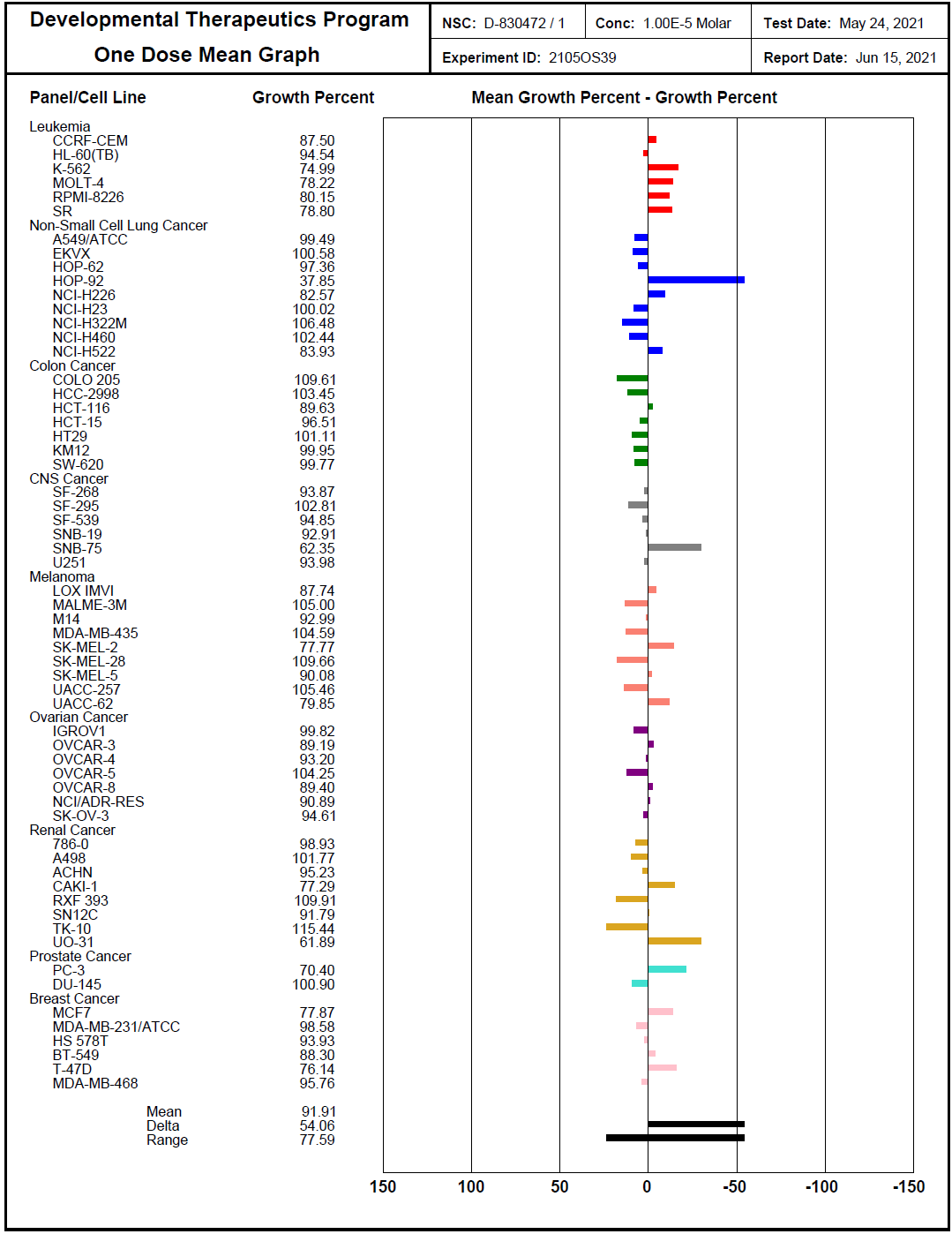


Figure S52. One- dose NCI growth (%) and mean graph of compound **6i**.


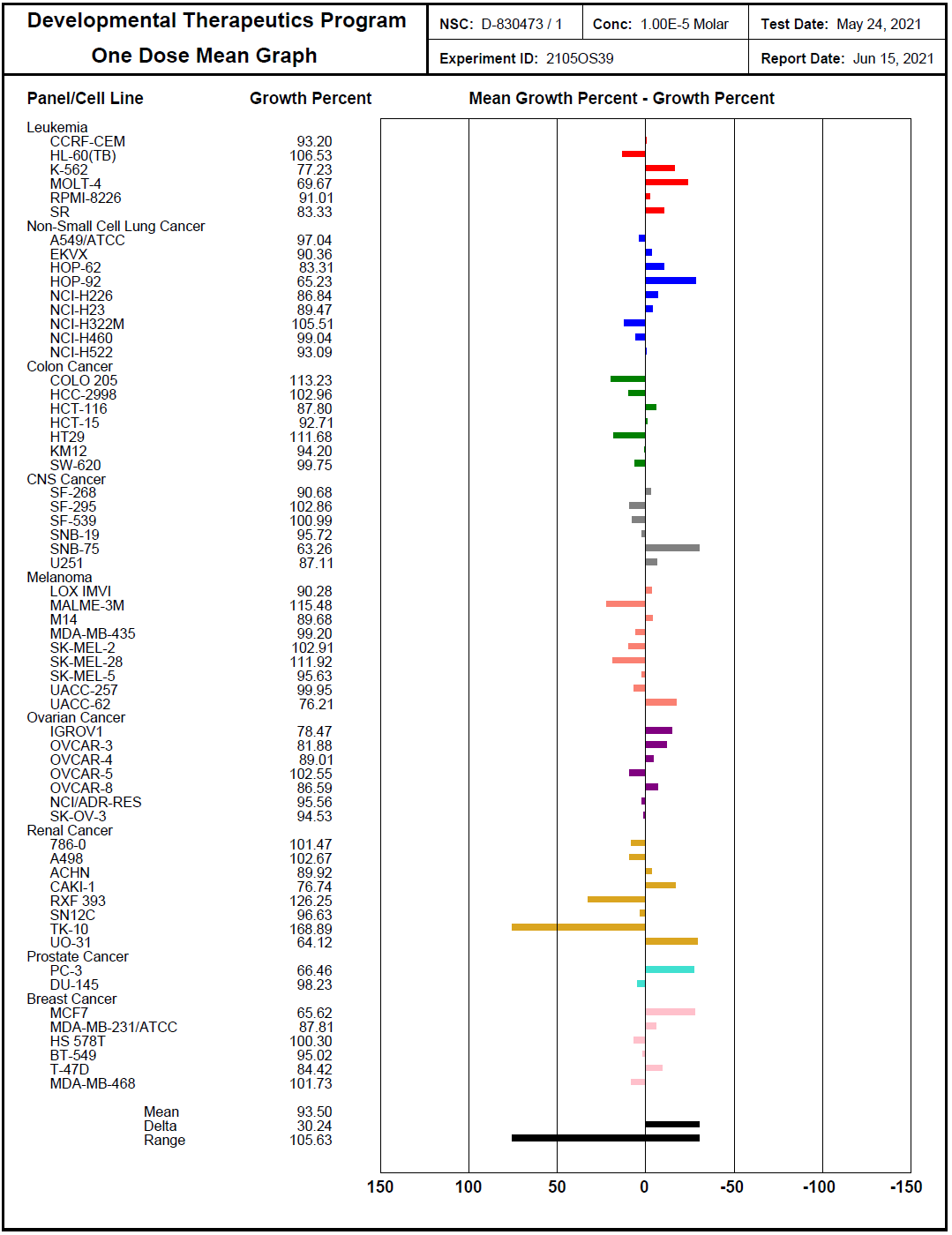


Figure S53. One- dose NCI growth (%) and mean graph of compound **6j**.


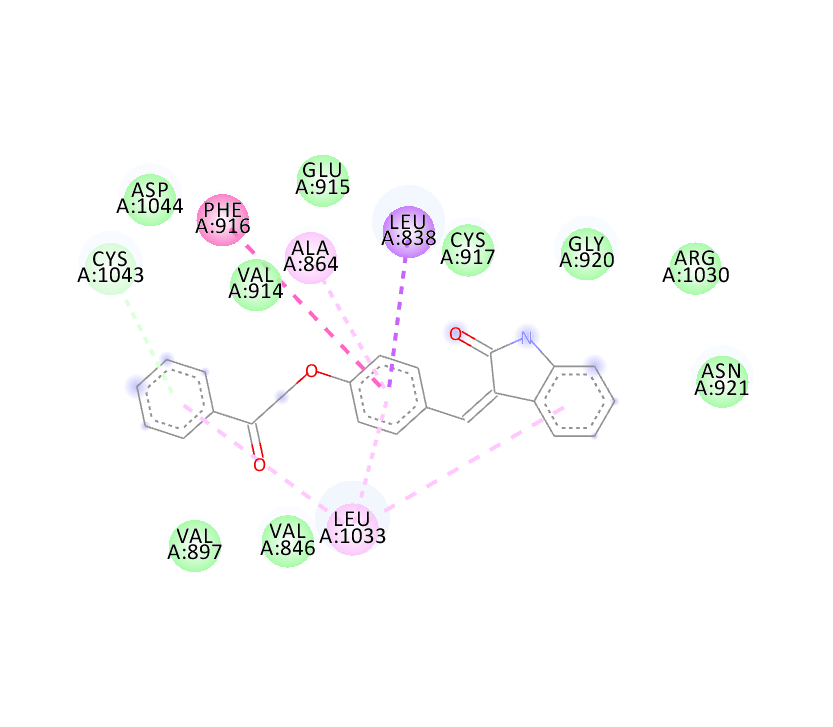


Figure S54. 2D diagram model showing compound **6a** interactions into the VEGFR-2 kinase binding site (PDB code: 1YWN).


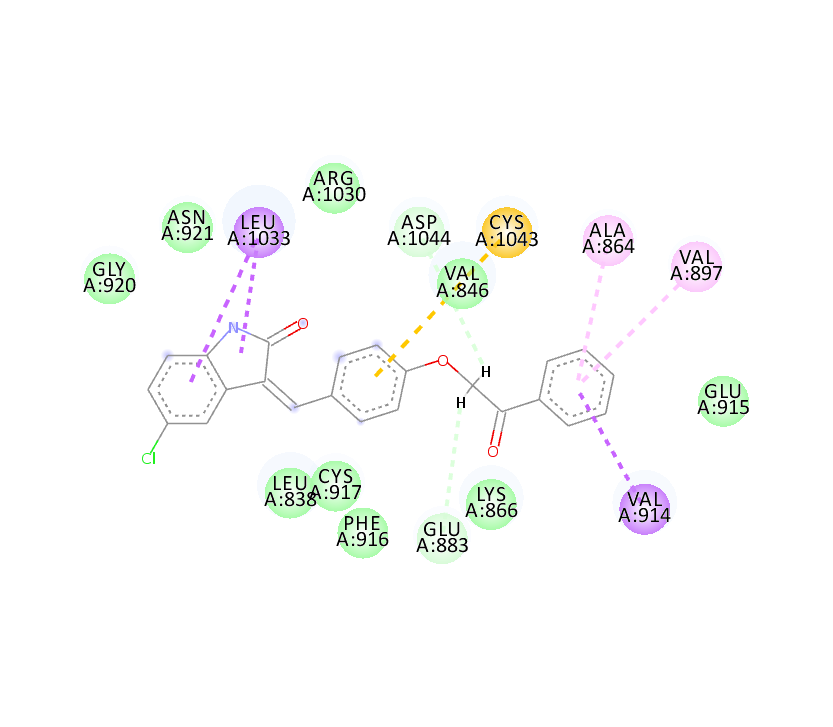


Figure S55. 2D diagram model showing compound **6b** interactions into the VEGFR-2 kinase binding site (PDB code: 1YWN).


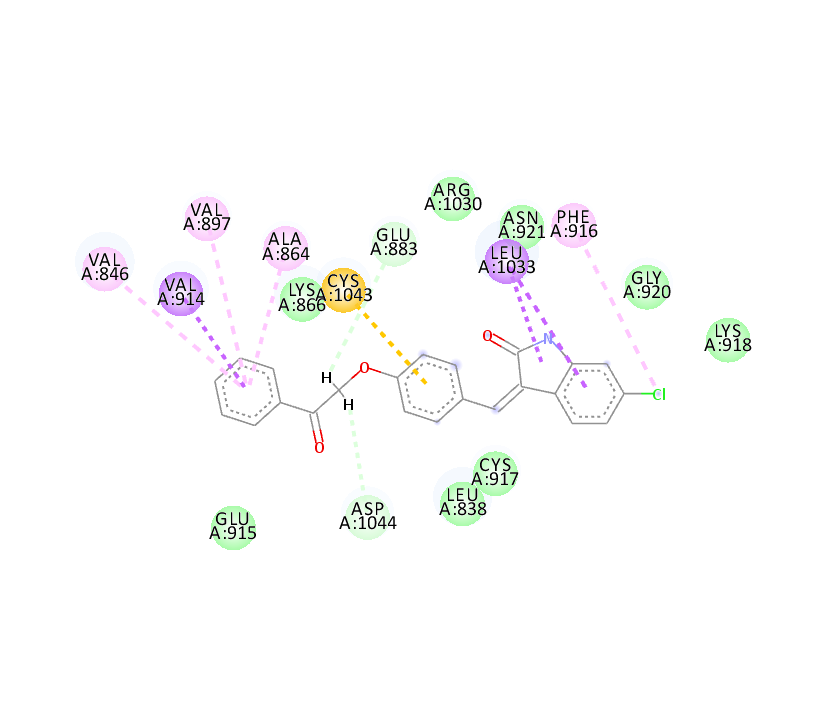


Figure S56. 2D diagram model showing compound **6c** interactions into the VEGFR-2 kinase binding site (PDB code: 1YWN).


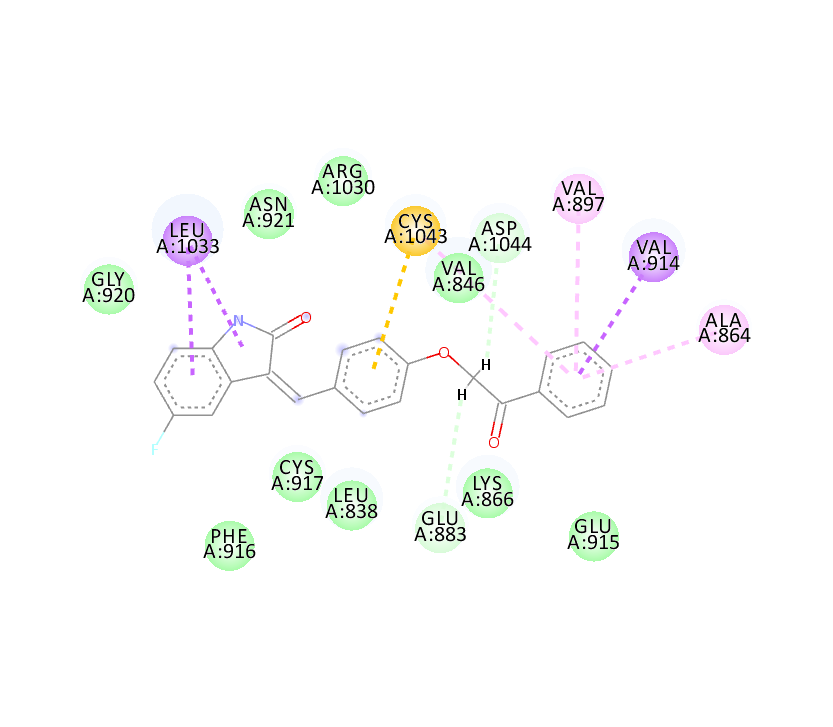


Figure S57. 2D diagram model showing compound **6d** interactions into the VEGFR-2 kinase binding site (PDB code: 1YWN).


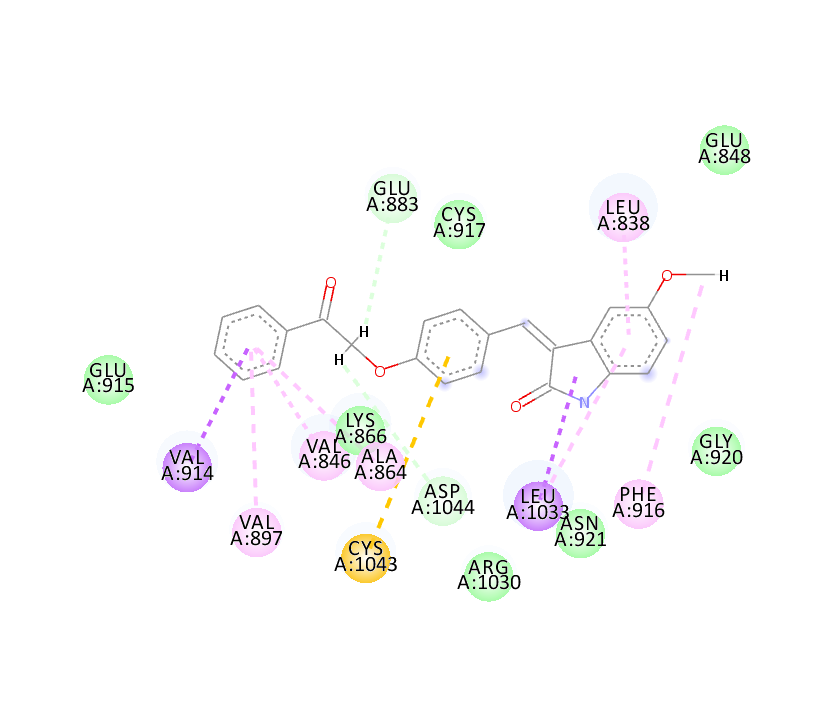


Figure S58. 2D diagram model showing compound **6e** interactions into the VEGFR-2 kinase binding site (PDB code: 1YWN).


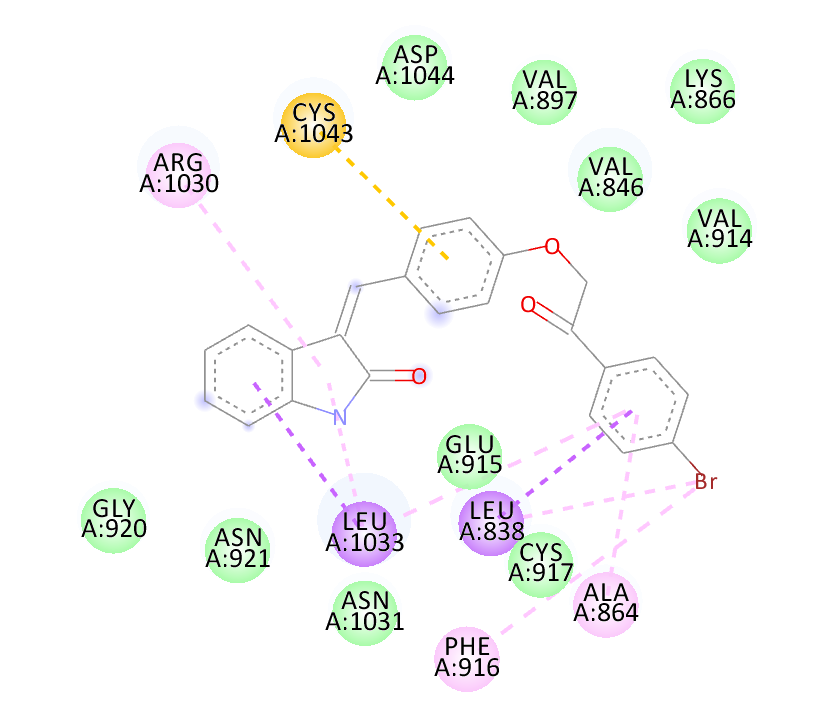


Figure S59. 2D diagram model showing compound **6f** interactions into the VEGFR-2 kinase binding site (PDB code: 1YWN).


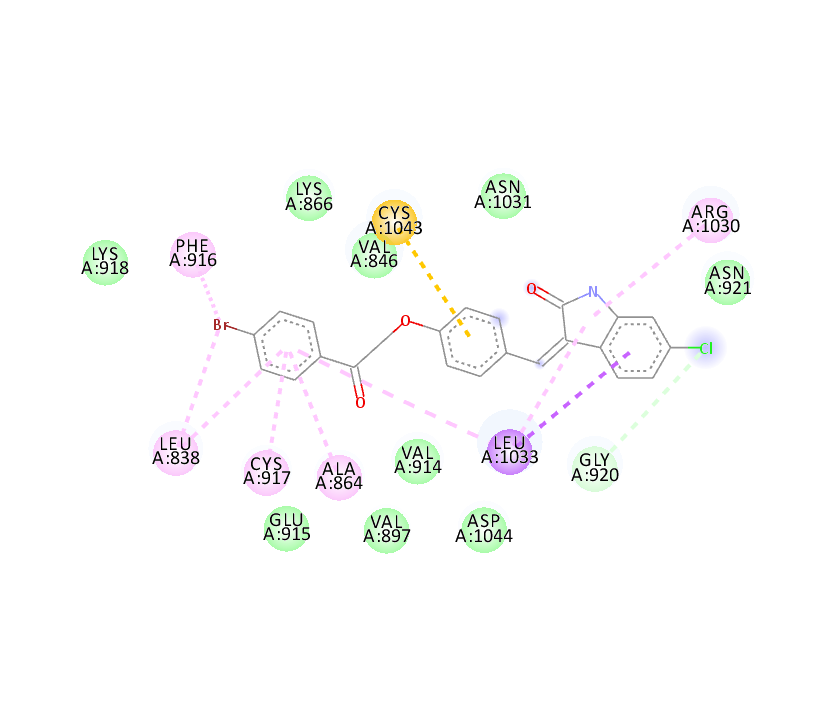


Figure S60. 2D diagram model showing compound **6g** interactions into the VEGFR-2 kinase binding site (PDB code: 1YWN).


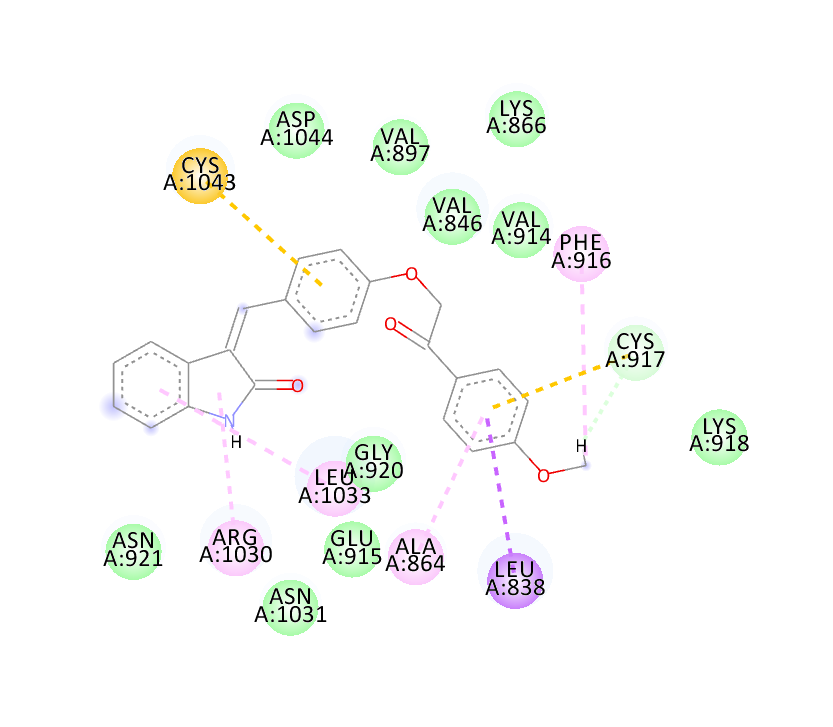


Figure S61. 2D diagram model showing compound **6h** interactions into the VEGFR-2 kinase binding site (PDB code: 1YWN).


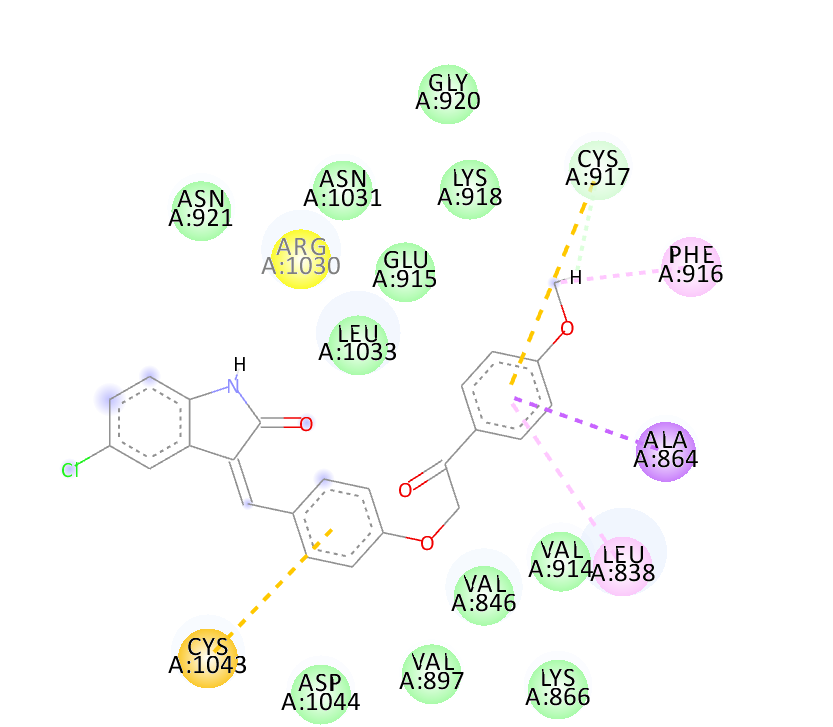


Figure S62. 2D diagram model showing compound **6i** interactions into the VEGFR-2 kinase binding site (PDB code: 1YWN).


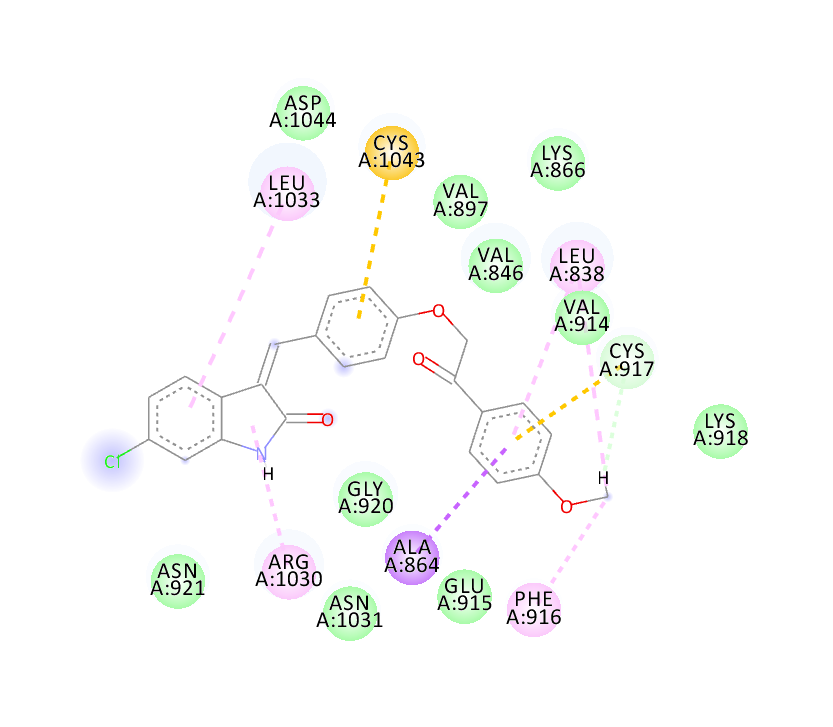


Figure S63. 2D diagram model showing compound **6j** interactions into the VEGFR-2 kinase binding site (PDB code: 1YWN).


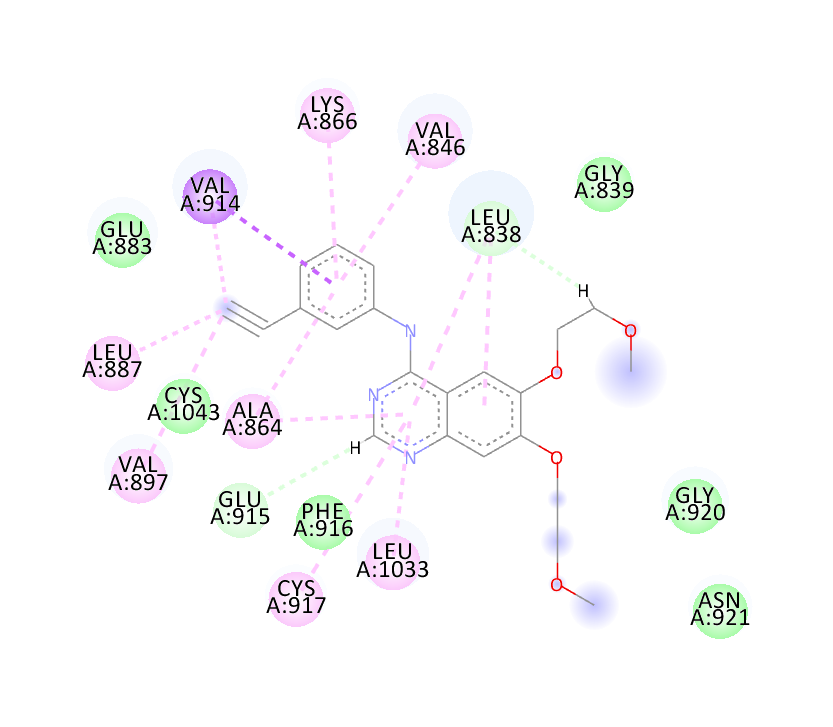


Figure S64. 2D diagram model showing **erlotinib** interactions into the VEGFR-2 kinase binding site (PDB code: 1YWN).


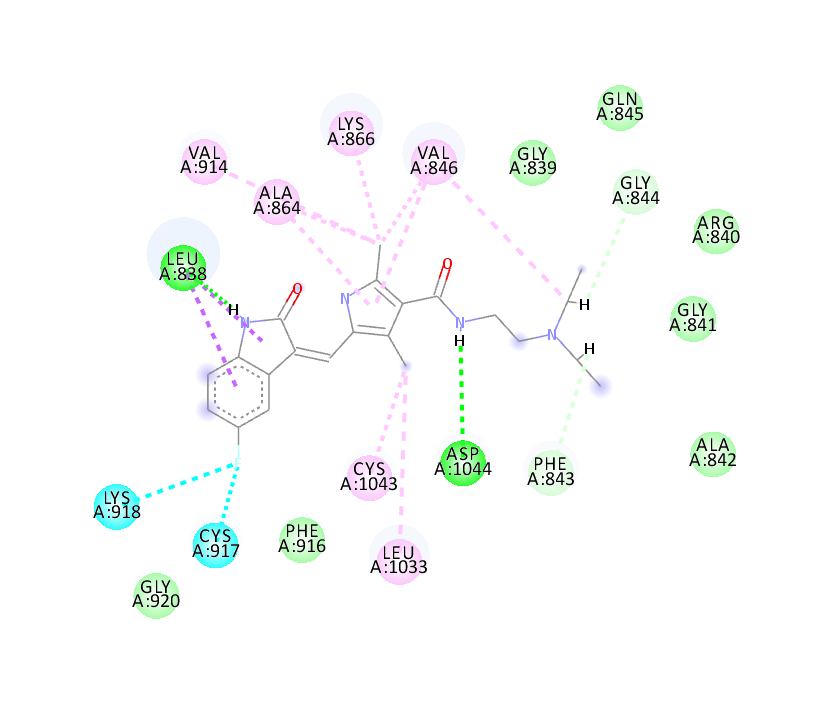


Figure S65. 2D diagram model showing **sunitinib** interactions into the VEGFR-2 kinase binding site (PDB code: 1YWN).


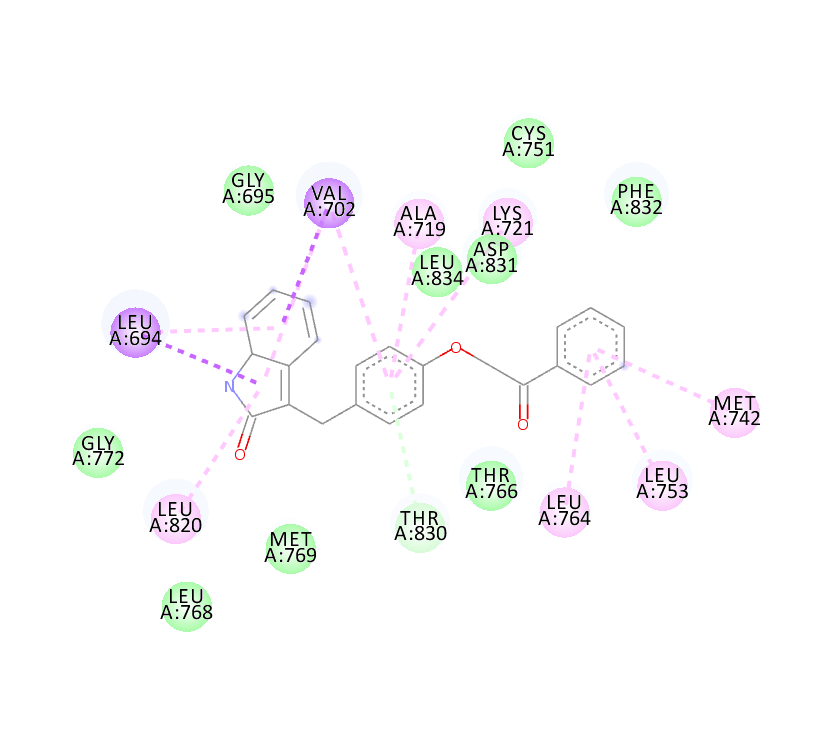


Figure S66. 2D diagram model showing compound **6a** interactions into the EGFR kinase binding site (PDB code: 4HJO).


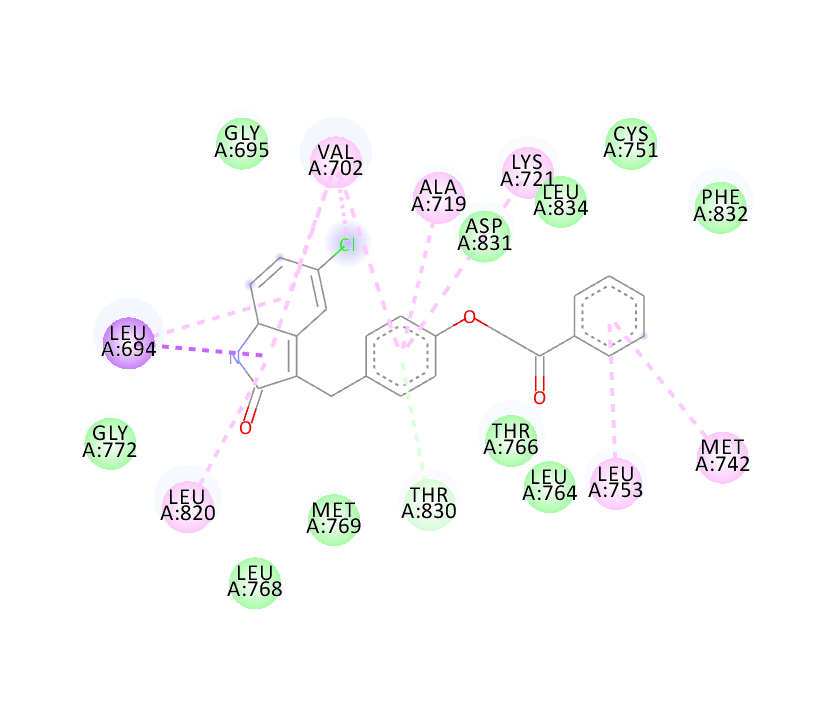


Figure S67. 2D diagram model showing compound **6b** interactions into the EGFR kinase binding site (PDB code: 4HJO).


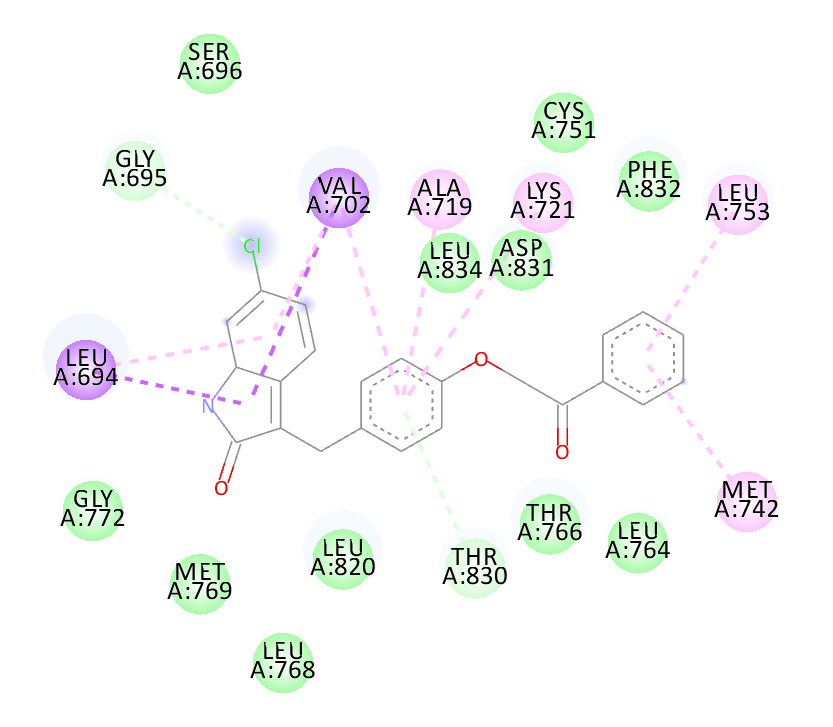


Figure S68. 2D diagram model showing compound **6c** interactions into the EGFR kinase binding site (PDB code: 4HJO).


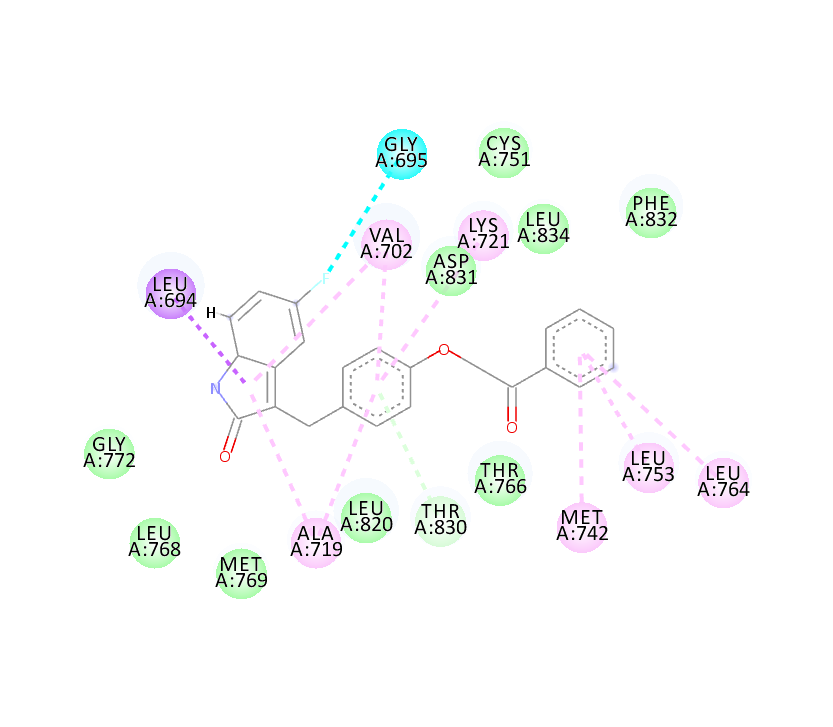


Figure S69. 2D diagram model showing compound **6d** interactions into the EGFR kinase binding site (PDB code: 4HJO).


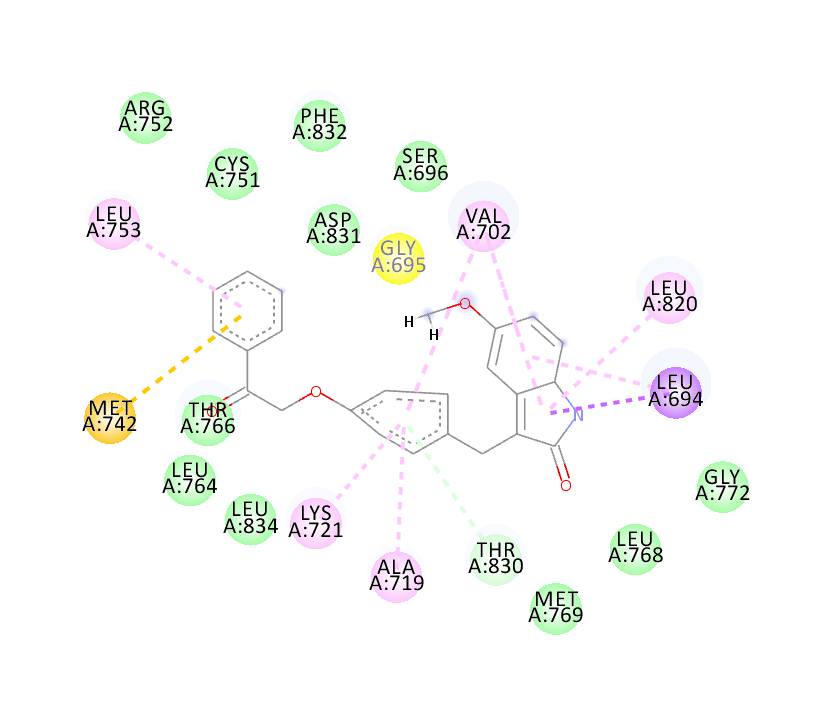


Figure S70. 2D diagram model showing compound **6e** interactions into the EGFR kinase binding site (PDB code: 4HJO).


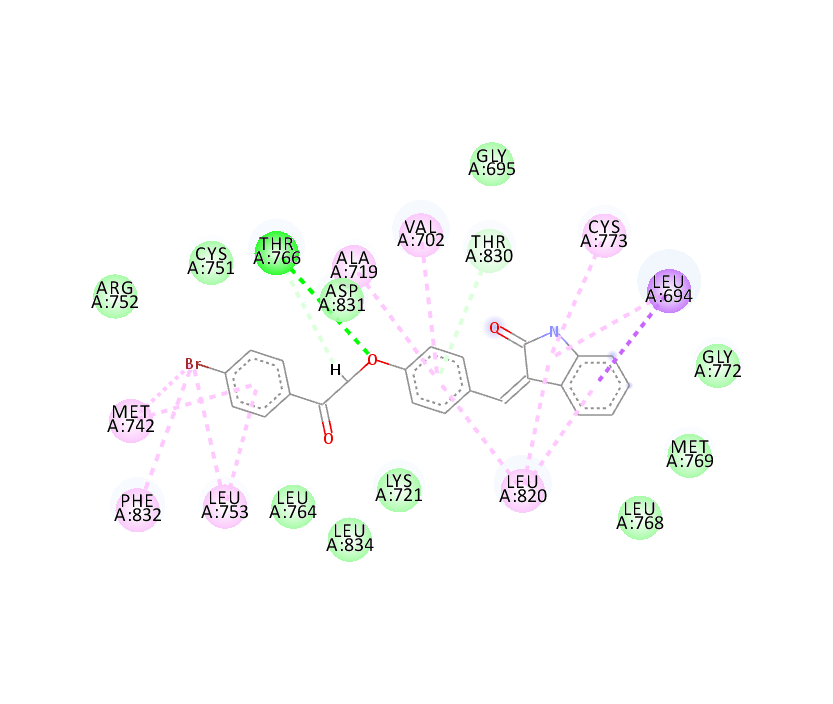


Figure S71. 2D diagram model showing compound **6f** interactions into the EGFR kinase binding site (PDB code: 4HJO).


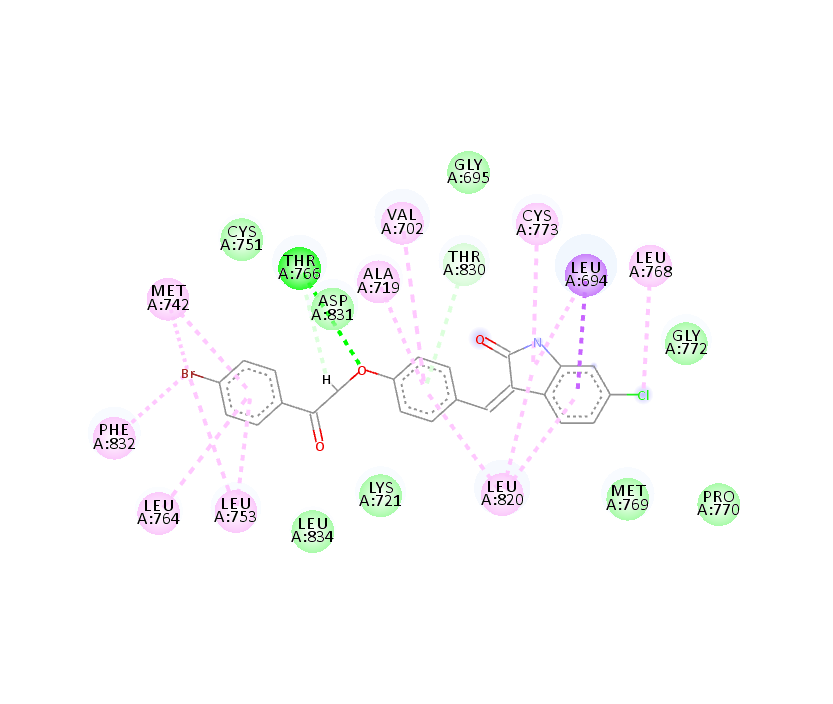


Figure S72. 2D diagram model showing compound **6g** interactions into the EGFR kinase binding site (PDB code: 4HJO).


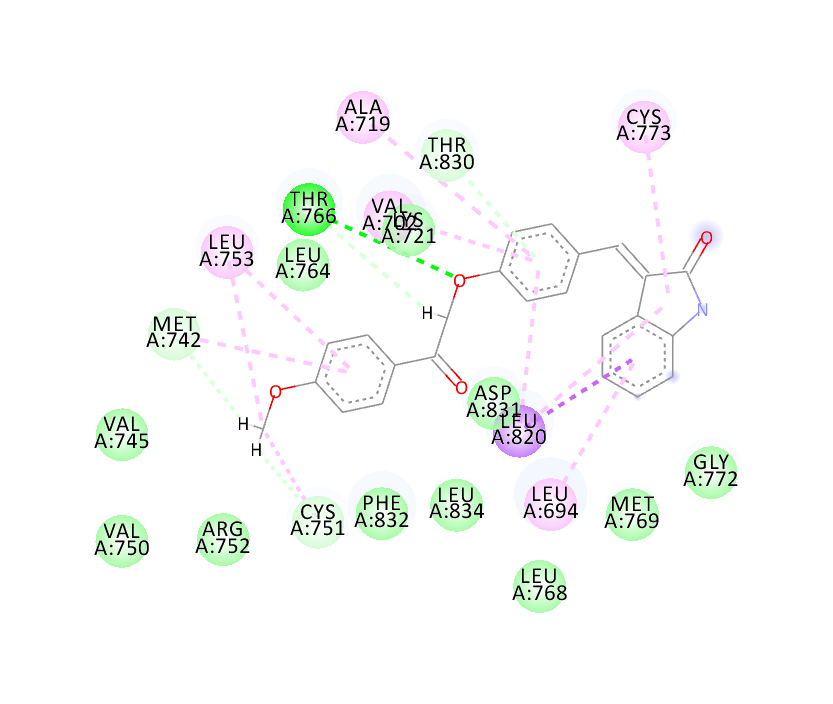


Figure S73. 2D diagram model showing compound **6h** interactions into the EGFR kinase binding site (PDB code: 4HJO).


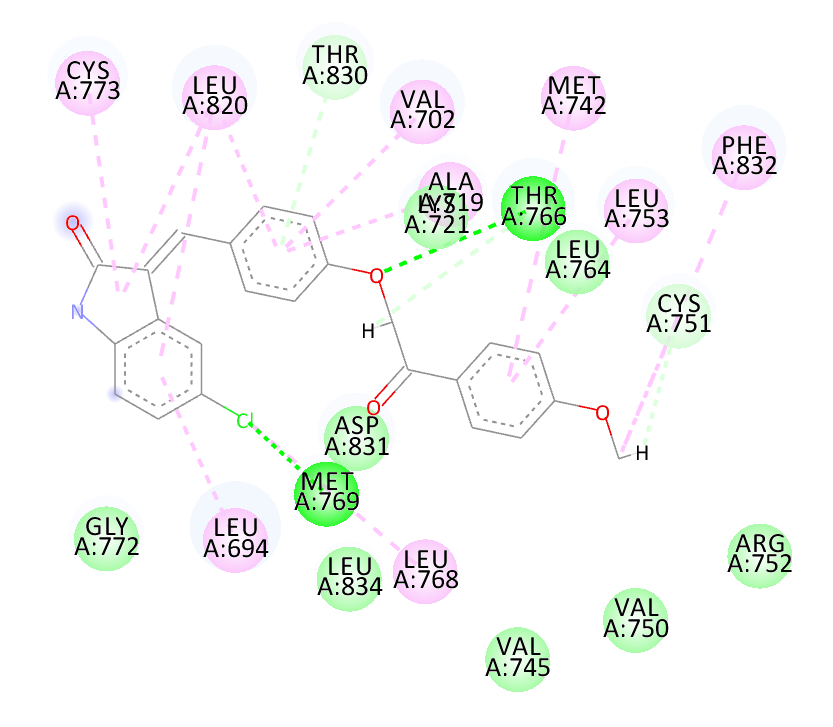


Figure S74. 2D diagram model showing compound **6i** interactions into the EGFR kinase binding site (PDB code: 4HJO).


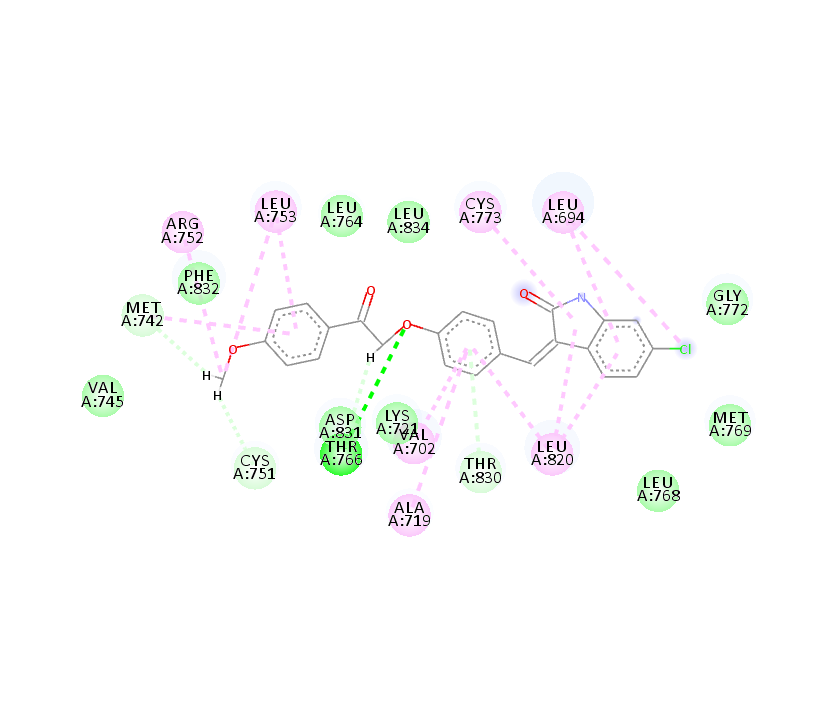


Figure S75. 2D diagram model showing compound **6j** interactions into the EGFR kinase binding site (PDB code: 4HJO).

Figure S76. 2D diagram model showing compound **erlotinib** interactions into the EGFR kinase binding site (PDB code: 4HJO).

Figure S77. 2D diagram model showing compound **sunitinib** interactions into the EGFR kinase binding site (PDB code: 4HJO).

Figure S78. 2D diagram model showing compound **6a** interactions into the CA-4 binding site of tubulin (PDB code: 5LYJ).

Figure S79. 2D diagram model showing compound **6b** interactions into the CA-4 binding site of tubulin (PDB code: 5LYJ).

Figure S80. 2D diagram model showing compound **6c** interactions into the CA-4 binding site of tubulin (PDB code: 5LYJ).

Figure S81. 2D diagram model showing compound **6d** interactions into the CA-4 binding site of tubulin (PDB code: 5LYJ).

Figure S82. 2D diagram model showing compound **6e** interactions into the CA-4 binding site of tubulin (PDB code: 5LYJ).

Figure S83. 2D diagram model showing compound **6f** interactions into the CA-4 binding site of tubulin (PDB code: 5LYJ).

Figure S84. 2D diagram model showing compound **6g** interactions into the CA-4 binding site of tubulin (PDB code: 5LYJ).

Figure S85. 2D diagram model showing compound **6h** interactions into the CA-4 binding site of tubulin (PDB code: 5LYJ).

Figure S86. 2D diagram model showing compound **6i** interactions into the CA-4 binding site of tubulin (PDB code: 5LYJ).

Figure S87. 2D diagram model showing compound **6j** interactions into the CA-4 binding site of tubulin (PDB code: 5LYJ).

Figure S88. 2D diagram model showing **combretastatin A-4 (CA-4)** interactions into the CA-4 binding site of tubulin (PDB code: 5LYJ).

| Table.1. Growth inhibition % of compounds 6a-j versus 60 NCI cancer cell lines using 10 uM as a single dose assay. | | | | | | | | | | | | |
| --- | --- | --- | --- | --- | --- | --- | --- | --- | --- | --- | --- | --- |
|  |  | **6a** | **6b** | | **6c** | **6d** | **6e** | **6f** | **6g** | **6h** | **6i** | **6j** |
| Leukemia | | | | | | | | | | | | |
| CCRF-CEM | | - | - | | - | 15.64 | - | 41.78 | 34.26 | 14.00 | 12.50 | - |
| HL-60(TB) | | - | - | | - | 16.89 | - | 59.6 | 53.84 | 20.79 | - | - |
| K-562 | | 11.09 | 23.51 | | 17.97 | 33.62 | 19.52 | 72.2 | 58.26 | 39.70 | 25.01 | 22.77 |
| MOLT-4 | | - | 13.37 | | - | 32.19 | 11.58 | **91.46** | 61.59 | 34.27 | 21.77 | 30.33 |
| RPMI-8226 | | - | - | | - | 22.12 | - | 50.83 | 43.63 | 25.45 | 19.85 | - |
| SR | | - | - | | - | 25.21 | - | 63.68 | 59.72 | 19.20 | 21.20 | 16.66 |
| Non-Small Cell Lung Cancer | | | | | | | | | | | | |
| A549/ATCC | | - | - | - | | - | - | 41.65 | 49.91 | - | - | - |
| EKVX | | - | - | - | | 18.89 | - | 34.38 | 43.76 | - | - | - |
| HOP-62 | | - | 14.57 | - | | 10.53 | - | - | - | - | - | 16.68 |
| HOP-92 | | - | - | - | | - | - | 24.53 | 30.80 | 11.73 | 62.14 | 34.77 |
| NCI-H226 | | 11.00 | 13.27 | 13.23 | | 10.26 | 11.63 | 28.42 | 37.86 | 15.39 | 17.43 | 13.16 |
| NCI-H23 | | - | - | - | | - | - | 49.07 | 46.84 | - | - | 10.52 |
| NCI-H322M | | - | - | 11.59 | | 11.18 | 12.43 | 12.49 | 18.12 | - | - | - |
| NCI-H460 | | - | - | - | | - | - | 41.53 | 40.98 | - | - | - |
| NCI-H522 | | - | - | 11.69 | | 12.36 | - | 38.59 | 49.40 | 17.19 | 16.06 | - |
| Colon Cancer | | | | | | | | | | | | |
| COLO 205 | | - | - | - | | - | - | 34.10 | 44.68 | - | - | - |
| HCC-2998 | | - | - | - | | - | - | 14.94 | 17.54 | - | - | - |
| HCT-116 | | - | - | 12.25 | | 14.33 | 12.03 | 44.58 | 42.09 | 25.39 | 10.36 | 12.19 |
| HCT-15 | | - | 11.88 | 23.46 | | 11.72 | 10.26 | 54.63 | 46.87 | 15.22 | - | - |
| HT29 | | - | - | - | | - | - | 17.25 | 16.10 | - | - | - |
| KM12 | | - | - | - | | 15.94 | - | 22.68 | 28.68 | - | - | - |
| SW-620 | | - | - | - | | - | - | 21.90 | 14.14 | - | - | - |
| CNS Cancer | | | | | | | | | | | | |
| SF-268 | | - | 11.32 | | 11.45 | 14.64 | - | 13.14 | 21.96 | - | - | - |
| SF-295 | | - | - | | - | - | - | 41.35 | 45.09 | - | - | - |
| SF-539 | | - | - | | 13.81 | 14.29 | - | 18.51 | 30.56 | 10.74 | - | - |
| SNB-19 | | - | - | | - | 14.92 | - | 20.41 | 18.50 | - | - | - |
| SNB-75 | | 26.18 | 31.13 | | 41.17 | 33.67 | - | 20.43 | 31.07 | 23.57 | 37.65 | 36.73 |
| U251 | | - | - | | - | 16.78 | - | 28.49 | 25.84 | - | - | 12.89 |
| Melanoma | | | | | | | | | | | | |
| LOX IMVI | | - | - | | 16.50 | 17.72 | 19.50 | **71.10** | 45.75 | - | 12.26 | - |
| MALME-3M | | - | - | | - | - | - | 18.99 | 12.93 | - | - | - |
| M14 | | - | - | | 11.07 | 12.07 | - | 26.90 | 31.05 | 12.11 | - | 10.31 |
| MDA-MB-435 | | - | - | | - | - | - | 29.50 | 24.82 | - | - | - |
| SK-MEL-2 | | - | - | | - | - | - | 27.56 | 29.84 | - | 22.22 | - |
| SK-MEL-28 | | - | - | | - | - | - | 19.85 | 19.36 | - | - | - |
| SK-MEL-5 | | - | - | | - | - | - | **71.12** | 61.24 | 17.76 | - | - |
| UACC-257 | | - | - | | - | - | - | 26.37 | 27.09 | - | - | - |
| UACC-62 | | 12.88 | 25.08 | | 27.90 | 30.61 | 24.70 | 47.43 | 42.02 | 31.52 | 20.14 | 23.79 |
| Ovarian Cancer | | | | | | | | | | | | |
|  | **IGROV1** | - | 20.29 | | 20.15 | - | - | 21.67 | 34.63 | 23.72 | - | 21.53 |
|  | **OVCAR-3** | - | 12.65 | | 10.75 | - | - | 22.47 | 15.68 | 18.33 | 10.81 | 18.11 |
|  | **OVCAR-4** | - | - | | - | - | - | 41.83 | 44.53 | - | - | 10.99 |
|  | **OVCAR-5** | - | - | | - | - | - | - | 13.91 | - | - | - |
|  | **OVCAR-8** | - | - | | - | - | - | 30.45 | 30.66 | 15.89 | 10.59 | 13.41 |
| NCI/ADR-RES | | 1.84 | - | | - | - | - | - | 52.24 | 43.66 | 13.06 | - |
|  | **SK-OV-3** | - | 11.90 | | - | - | - | - | - | 14.05 | - | - |
| Renal Cancer | | | | | | | | | | | | |
|  | **786-0** | - | - | - | | - | - | 10.24 | 15.62 | - | - | - |
|  | **A498** | - | - | - | | - | - | - | - | - | - | - |
|  | **ACHN** | - | - | 15.91 | | - | 11.17 | 30.01 | 31.19 | - | - | 10.07 |
|  | **CAKI-1** | 13.11 | 31.86 | 26.40 | | 28.29 | 19.79 | 32.18 | 47.57 | 26.37 | 22.71 | 23.25 |
|  | **RXF 393** | - | - | 16.45 | | - | - | 32.38 | 44.76 | - | - | - |
|  | **SN12C** | - | - | - | | - | - | 37.32 | 45.24 | - | - | - |
|  | **TK-10** | - | - | - | | - | - | - | - | - | - | - |
|  | **UO-31** | 30.48 | 39.51 | 34.99 | | 32.33 | 29.57 | 51.52 | 59.44 | 37.49 | 38.11 | 35.88 |
| Prostate Cancer | | | | | | | | | | | | |
|  | **PC-3** | 17.14 | 24.92 | 28.46 | | 23.01 | 14.85 | 37.59 | 51.35 | 36.14 | 29.59 | 33.53 |
|  | **DU-145** | - | - | - | | - | - | 14.43 | 12.36 | - | - | - |
| Breast Cancer | | | | | | | | | | | | |
|  | **MCF7** | 11.29 | 38.02 | 38.18 | | 23.05 | 20.22 | **72.61** | **70.03** | 41.29 | 22.12 | 34.38 |
|  | **MDA-MB-231/ATCC** | - | 15.49 | - | | - | - | 17.77 | 23.44 | 10.82 | - | 12.19 |
|  | **HS 578T** | - | 13.73 | 12.57 | | 14.81 | - | 15.55 | 22.37 | 11.35 | - | - |
|  | **BT-549** | - | - | - | | 10.94 | - | 43.44 | 44.30 | 10.06 | 11.70 | - |
|  | **T-47D** | - | - | 16.33 | | 14.33 | - | 53.05 | 55.17 | 26.44 | 23.85 | 15.57 |
|  | **MDA-MB-468** | - | - | - | | - | - | 47.62 | 42.28 | - | - | - |

| **Table.2.** Molecular modeling results for the synthesized compounds **6a-j**, **sunitinib,** and **erlotinib** at the active binding site of VEGFR-2 protein kinase (**PDB code: 1YWN**). | | | | |
| --- | --- | --- | --- | --- |
| **Compound** | **Docking** **Score (kcal/mol)** | **RMSD_Refine (Å)** | **Amino Acid/Bond** | **E (kcal/mol)** |
| **6a** | -7.6 | 1.2 | Glu883/H acceptor  Lys-866/ H acceptor  Val-914/ ᴫ-σ  Val-846/ ᴫ-σ | -5.5  -4.7  -1.4  -1.2 |
| **6b** | -8.9 | 1.3 | Glu883/H acceptor  Lys-866/ H acceptor  Leu1033/ ᴫ-σ  Val-914/ ᴫ-σ  Val-846/ ᴫ-σ  Leu-838/van der Waals | -5.8  -5.3  -1.5  -1.8  -1.1  -0.9 |
| **6c** | -8.3 | 1.1 | Glu883/H acceptor  Lys-866/ H acceptor  Leu1033/ ᴫ-σ  Val-914/ ᴫ-σ  Val-846/ ᴫ-σ | -5.6  -5.2  -1.8  -1.3  -1.1 |
| **6d** | -8.2 | 1.4 | Glu883/H acceptor  Lys-866/ H acceptor  Leu1033/ ᴫ-σ  Val-914/ ᴫ-σ  Val-846/ ᴫ-σ | -5.7  -5.3  -1.6  -1.6  -1.2 |
| **6e** | -8.1 | 1.3 | Glu883/H acceptor  Arg-1030/ H donor  Leu1033/ ᴫ-σ  Val-914/ ᴫ-σ  Val-846/ ᴫ-σ | -5.7  -5.3  -1.6  -1.6  -1.2 |
| **6f** | -8.6 | 1.5 | Asn-1031/H acceptor  Lys-866/ H acceptor Leu1033/ ᴫ-σ  Val-914/ ᴫ-σ  Leu-838/van der Waals | -5.6  -5.5  -1.6  -1.7  -1.1 |
| **6g** | -8.5 | 1.7 | Asn-1031/H acceptor  Lys-866/ H acceptor Leu1033/ ᴫ-σ  Val-914/ ᴫ-σ  Leu-838/van der Waals | -5.5  -5.6  -1.3  -1.5  -1.4 |
| **6h** | -8.0 | 1.3 | Asn-1031/H acceptor  Lys-866/ H acceptor Leu1033/ ᴫ-σ  Val-914/ ᴫ-σ  Leu-838/van der Waals | -5.5  -5.6  -1.1  -1.2  -1.6 |
| **6i** | -8.1 | 1.1 | Asn-1031/H acceptor  Lys-866/ H acceptor Leu1033/ ᴫ-σ  Val-914/ ᴫ-σ  Leu-838/van der Waals | -5.4  -5.2  -1.7  -1.6  -1.5 |
| **6j** | -7.8 | 1.3 | Asn-1031/H acceptor  Lys-866/ H acceptor Leu1033/ ᴫ-σ  Val-914/ ᴫ-σ  Leu-838/van der Waals | -5.5  -5.6  -1.2  -1.4  -1.4 |
| **Sunitinib** | -8.5 | 1.7 | Lys-866/ H acceptor  Leu-838/ H acceptor  Leu1033/ ᴫ-σ  Val-846/ ᴫ-σ | -1.9  -1.0  -2.6  1.5 |
| **Erlotinib** | -7.5 | 1.4 | Leu1033/ ᴫ-σ  Val-846/ ᴫ-σ  Lys-866/ ᴫ-σ  Leu-838/van der Waals | -4.4  -1.6  -1.1  -0.8 |

| **Table 3.** Molecular modeling results for the synthesized compounds **6a-j**, **sunitinib,** and **erlotinib** at the active binding site of EGFR protein kinase (**PDB code: 4HJO**). | | | | |
| --- | --- | --- | --- | --- |
| **Compound** | **Docking** **Score**  **kcal/mol** | **RMSD_Refine**  **(Å)** | **Amino Acid/Bond** | **E (kcal/mol)** |
| **6a** | -8.2 | 1.4 | Leu-834/ ᴫ-σ  Leu-820/ ᴫ-σ  Lys-721/ ᴫ-σ  Val-702/ ᴫ-σ | -1.4  -1.7  -1.7  -1.1 |
| **6b** | -8.8 | 1.1 | Leu-834/ ᴫ-σ  Leu-820/ ᴫ-σ  Lys-721/ ᴫ-σ  Val-702/ ᴫ-σ | -1.5  -1.3  -1.2  -1.6 |
| **6c** | -8.5 | 1.3 | Leu-834/ ᴫ-σ  Leu-820/ ᴫ-σ  Lys-721/ ᴫ-σ  Val-702/ ᴫ-σ | -1.3  -1.1  -1.3  -1.7 |
| **6d** | -8.0 | 1.2 | Leu-834/ ᴫ-σ  Leu-820/ ᴫ-σ  Lys-721/ ᴫ-σ  Val-702/ ᴫ-σ | -1.3  -1.6  -1.4  -1.1 |
| **6e** | -8.1 | 1.3 | Leu-834/ ᴫ-σ  Leu-820/ ᴫ-σ  Lys-721/ ᴫ-σ  Val-702/ ᴫ-σ | -5.7  -5.3  -1.6  -1.6 |
| **6f** | -10.6 | 1.2 | Thr-766/ H acceptor  Lys-721/ H donor  Leu-820/ ᴫ-σ  Cys-773/ alkyl  Leu-753/ ᴫ-σ  Met-742/ alkyl  Val-702/ ᴫ-σ  Leu-649/ ᴫ-σ | -5.2  -5.1  -1.6  -0.7  -1.1  -0.6  -1.1  -1.2 |
| **6g** | -10.1 | 1.3 | Thr-766/ H acceptor  Lys-721/ H donor  Leu-820/ ᴫ-σ  Cys-773/ alkyl  Leu-753/ ᴫ-σ  Met-742/ alkyl  Val-702/ ᴫ-σ  Leu-649/ ᴫ-σ | -5.4  -5.3  -1.1  -0.6  -1.2  -0.5  -1.3  -1.1 |
| **6h** | -9.3 | 1.6 | Thr-766/ H acceptor  Leu-820/ ᴫ-σ  Leu-753/ ᴫ-σ  Val-702/ ᴫ-σ  Leu-649/ ᴫ-σ | -5.7  -1.6  -1.3  -1.1  -1.4 |
| **6i** | -8.6 | 1.1 | Thr-766/ H acceptor  Leu-820/ ᴫ-σ  Leu-753/ ᴫ-σ  Val-702/ ᴫ-σ  Leu-649/ ᴫ-σ | -5.5  -1.2  -1.1  -1.6  -1.2 |
| **6j** | -8.8 | 1.4 | Thr-766/ H acceptor  Leu-820/ ᴫ-σ  Leu-753/ ᴫ-σ  Val-702/ ᴫ-σ  Leu-649/ ᴫ-σ | -5.2  -1.1  -1.5  -1.3  -1.1 |
| **Sunitinib** | -8.5 | 1.5 | Lys-721/ H acceptor  Leu-820/ ᴫ-σ  Val-702/ ᴫ-σ  Cys-773/ alkyl  Thr-830/ van der Waals | -5.6  -1.0  -1.6  -0.5  -0.9 |
| **Erlotinib** | -7.5 | 1.2 | Met-769/ H acceptor  Lys-721/ ᴫ-σ  Leu-820/ ᴫ-σ  Val-702/ ᴫ-σ  Leu-694/ ᴫ-σ  Cys-773/ alkyl | -4.9  -1.5  -1.2  -1.3  -1.1  -0.7 |

| **Table 4.** Molecular modeling results for the synthesized compounds **6a-j,** and **CA-4** at the colchicine binding site of tubulin (**PDB code: 5LYJ**). | | | | |
| --- | --- | --- | --- | --- |
| **Compound** | **Docking** **Score**  **kcal/mol** | **RMSD_Refine**  **(Å)** | **Amino Acid/Bond** | **E (kcal/mol)** |
| **6a** | -7.6 | 1.1 | Val-238/H donor  Leu-255/ ᴫ-σ  Asn-258/ ᴫ-σ  Ala-316/ ᴫ-alkyl  Cys-241/ van der Waals | -4.7  -1.7  -1.5  -0.5  -0.7 |
| **6b** | -7.9 | 1.6 | Val-238/H donor  Leu-255/ ᴫ-σ  Asn-258/ ᴫ-σ  Ala-316/ ᴫ-alkyl  Cys-241/ van der Waals | -5.1  -1.2  -1.2  -0.6  -0.5 |
| **6c** | -7.1 | 1.0 | Val-238/H donor  Leu-255/ ᴫ-σ  Asn-258/ ᴫ-σ  Ala-316/ ᴫ-alkyl  Cys-241/ van der Waals | -4.9  -1.5  -1.1  -0.5  -0.4 |
| **6d** | -7.3 | 1.7 | Val-238/H donor  Leu-255/ ᴫ-σ  Asn-258/ ᴫ-σ  Ala-316/ ᴫ-alkyl  Cys-241/ van der Waals | -4.7  -1.1  -1.6  -0.8  -0.5 |
| **6e** | -7.6 | 1.6 | Val-238/H donor  Leu-255/ ᴫ-σ  Asn-258/ ᴫ-σ  Ala-316/ ᴫ-alkyl  Cys-241/ van der Waals | -4.8  -1.3  -1.3  -0.6  -0.6 |
| **6f** | -10.2 | 1.1 | Val-238/H donor  Asn-258/ H acceptor  Lys-352/ ᴫ-σ  Ile-318/ ᴫ-σ  Leu-248/ ᴫ-σ  Ala-250/ ᴫ-alkyl  Cys-241/ van der Waals | -4.8  -5.2  -1.3  -1.4  -1.5  -0.9  -0.7 |
| **6g** | -8.9 | 1.7 | Val-238/H donor  Lys-352/ ᴫ-σ  Ile-318/ ᴫ-σ  Ala-250/ ᴫ-alkyl  Cys-241/ van der Waals | -4.9  -1.2  -1.5  -0.7  -0.6 |
| **6h** | -9.5 | 1.6 | Val-238/H donor  Lys-352/ ᴫ-σ  Ile-318/ ᴫ-σ  Ala-250/ ᴫ-alkyl  Cys-241/ van der Waals | -4.5  -1.5  -1.3  -0.8  -0.7 |
| **6i** | -9.1 | 1.8 | Val-238/H donor  Lys-352/ ᴫ-σ  Ile-318/ ᴫ-σ  Ala-250/ ᴫ-alkyl  Cys-241/ van der Waals | -4.5  -1.0  -1.4  -0.8  -0.5 |
| **6j** | -9.6 | 1.6 | Val-238/H donor  Lys-352/ ᴫ-σ  Ile-318/ ᴫ-σ  Ala-250/ ᴫ-alkyl  Cys-241/ van der Waals | -4.7  -1.5  -1.5  -0.7  -0.6 |
| **Combretastatin A4** | -11.3 | 1.3 | Lys-352/ ᴫ-σ  Ile-318/ ᴫ-σ  Leu-248/ ᴫ-σ  Ala-250/ ᴫ-alkyl  Cys-241/ van der Waals | -1.6  -1.5  -1.4  -0.7  -0.4 |
